# Supplementary material for: The genomes of 204 Vitis vinifera accessions reveal the origin of European wine grapes
Source: Nat Commun. 2021 Dec 21;12:7240. doi: 10.1038/s41467-021-27487-y (PMC8692429; doi:10.1038/s41467-021-27487-y)
Supplement: Supplementary file 1 — Supplementary Information [file 41467_2021_27487_MOESM1_ESM.pdf]

The genomes of 204 *Vitis vinifera* accessions reveal the origin of European wine grapes

Magris *et al*.

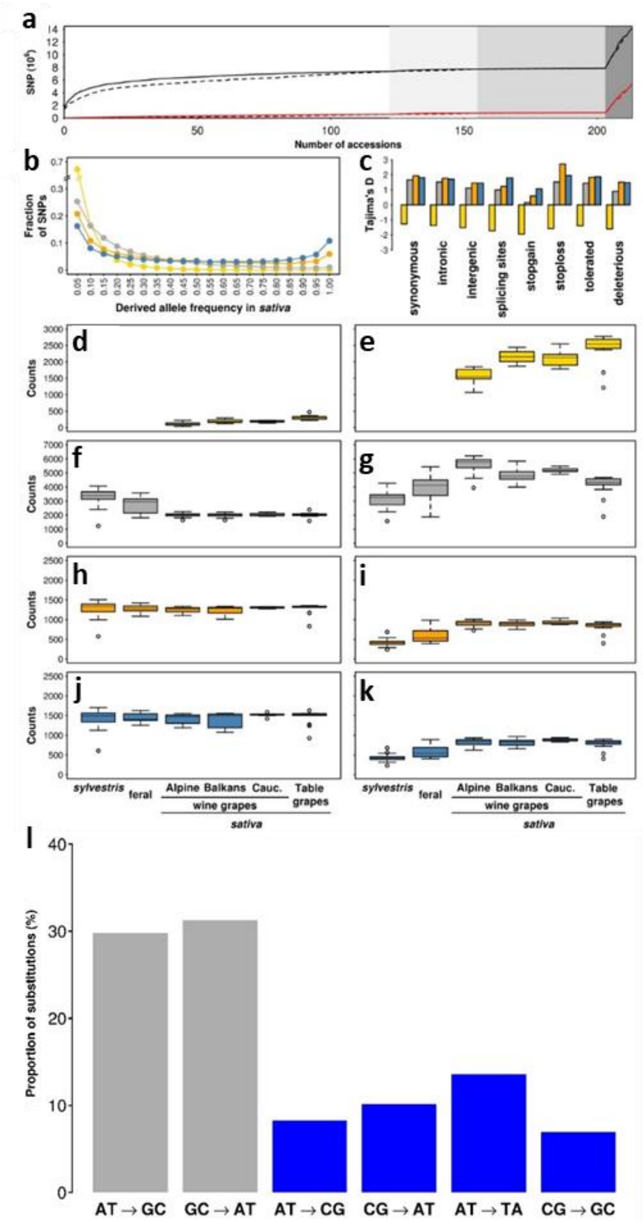

Supplementary Figure 1. Total SNPs in *vinifera* and outgroups (a), site frequency spectrum (b) and strength/direction of selective pressure in cultivated varieties (*sativa*) by SNP age and by mutation type (c), range of variation in the number of deleterious mutations per individual in different groups of *vinifera* by SNP age (d). a Total number of SNPs with respect to the reference grapevine genome in cultivated varieties (white background), feral (light grey background) and wild (grey background) *vinifera* and other grapes species (dark grey background). Black lines indicate all SNPs. Red lines indicate private SNPs. Accessions are ordered by decreasing number of SNPs compared to the reference (solid lines) and in reverse order (dashed lines). **b** Derived allele frequency spectrum in *sativa*. **c** Tajima's *D* by age and by genomic context or type of the mutation in *sativa*. **d**–**k**: Number of homozygous (**d**, **f**, **h**, **j**) and heterozygous (**e**, **g**, **i**, **k**) deleterious mutations per individual. Individuals were grouped by type. SNPs were sorted in all panels by evolutionary age: ■ predating Eurasian–American split, ■ predating Asian–European split, ■ $_{Vv}$SNPs predating domestication, ■ $_{sat}$SNPs. Boxes indicate

the first and third quartiles, the horizontal line within the boxes indicates the median and the whiskers indicate ± 1.5 × interquartile range. Boxes illustrate *n* = 48 (*sylvestris*), *n* = 33 (feral), *n* = 25 (Alpine), *n* = 16 (Balkans), *n* = 10 (Cauc.) and *n* = 14 (Table) accession of *sativa*, respectively. Abbreviations for the *sativa* groups stand for Alpine: Alpine wine grapes; Balkans: Balkans and *Magna Graecia*; Cauc.: Caucasian wine grapes; Table: Table grapes. Group composition is defined as reported in the source data of Supplementary Fig. 10. **l** Percentage of six types of SNP mutations. Transitions are indicated by grey histograms, transversions by blue histograms.

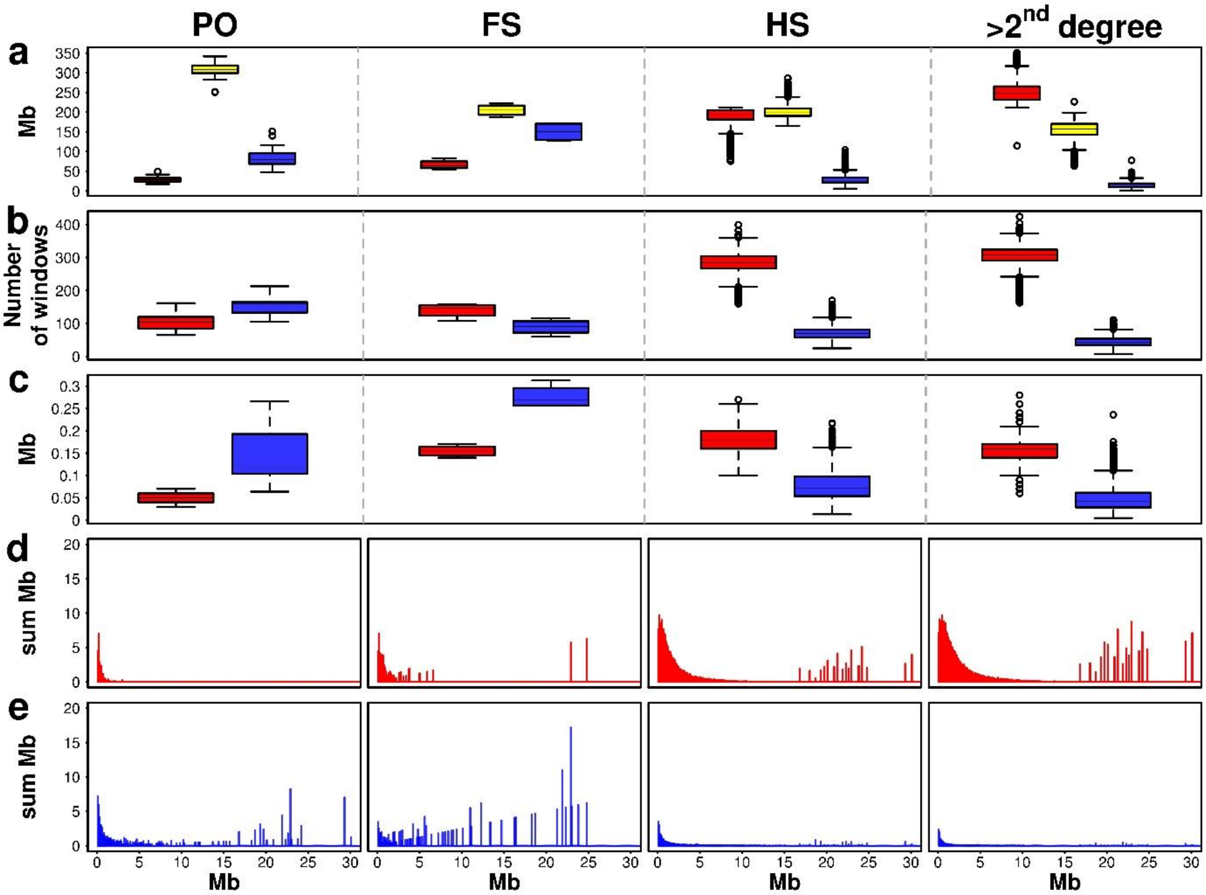

Supplementary Figure 2. Distribution of IBD parameters in pairwise comparisons between genotypes with different levels of consanguinity. a Box plots of aggregate lengths of IBD=0 (red, no haplotype shared), IBD=1 (yellow, one haplotype shared), IBD=2 (blue, two haplotype shared) genomic windows. **b** Box plots of the number of IBD=0 and IBD=2 genomic windows. **c** Box plots of the length of IBD=0 and IBD=2 segments. **a-c** Boxes indicate the first and third quartiles, the horizontal line within the boxes indicates the median and the whiskers indicate ± 1.5 × interquartile range. Boxes illustrate *n* = 25 (PO), *n* = 4 (FS), *n* = 2,003 (HS), *n* = 7,149 (>2nd degree) pairwise comparisons, respectively. **d** Cumulative length (sum Mb) of IBD=0 segment classes. **e** Cumulative length (sum Mb) of IBD=2 segment classes. PO: parent–offspring; FS: full–siblings; HS half–siblings.

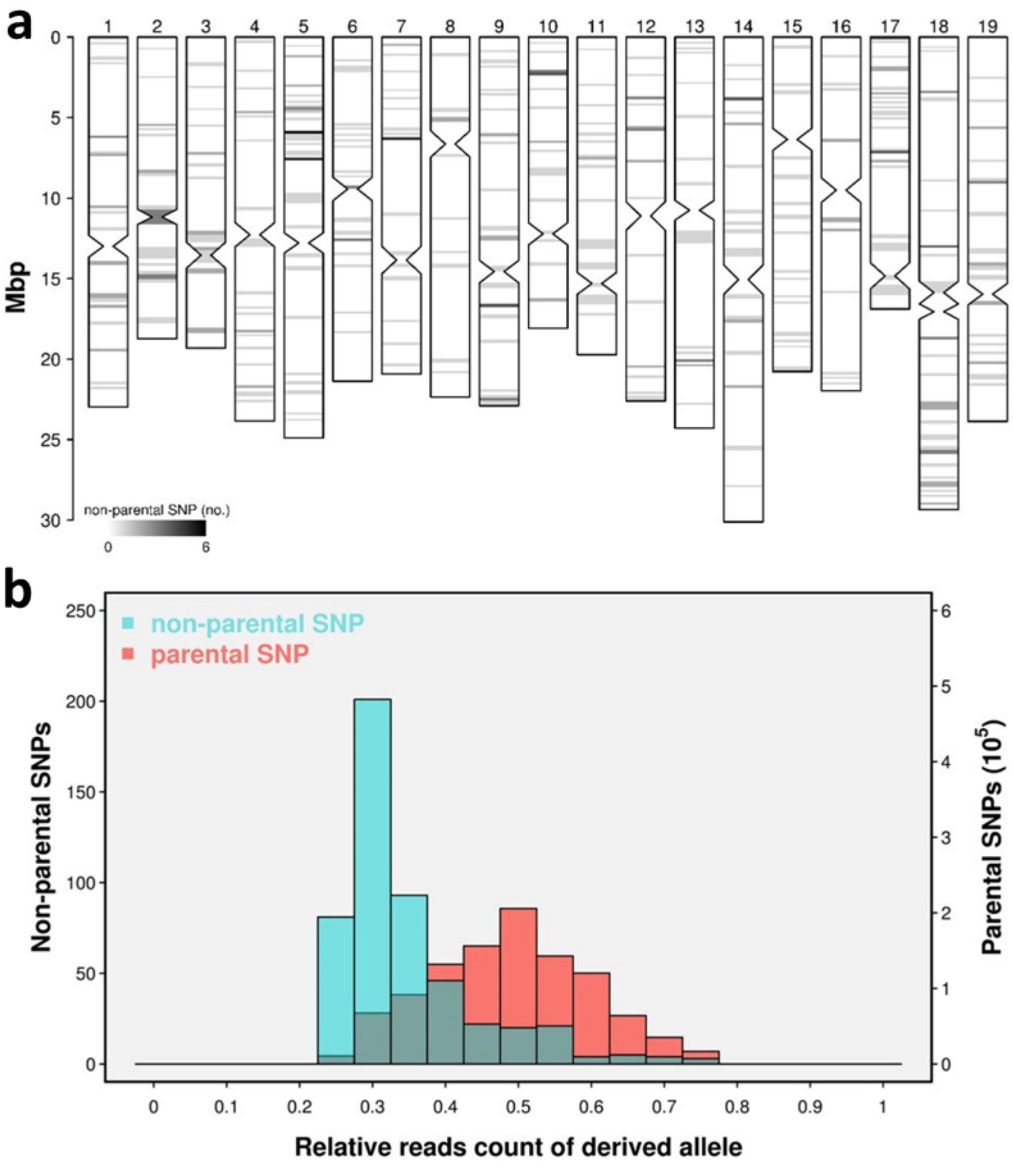

Supplementary Figure 3. Non–parental SNPs in Cabernet Sauvignon. a Chromosomal distribution of Mendelian inconsistencies. Constricted regions indicate the position of centromeric repeats. The color scale indicates the number of non–parental SNPs in each 100–Kb window of non–repetitive DNA. **b** Relative read counts of derived alleles in Cabernet Sauvignon for 1.6 million germline SNPs compared to the reference genome and 507 Mendelian inconsistencies reported in (**a**). The primary *y*–axis refers to the number of non–parental SNPs. The secondary *y*–axis refers to the number of germline SNP. The shadowed area of the histogram represents overlap between the two distributions.

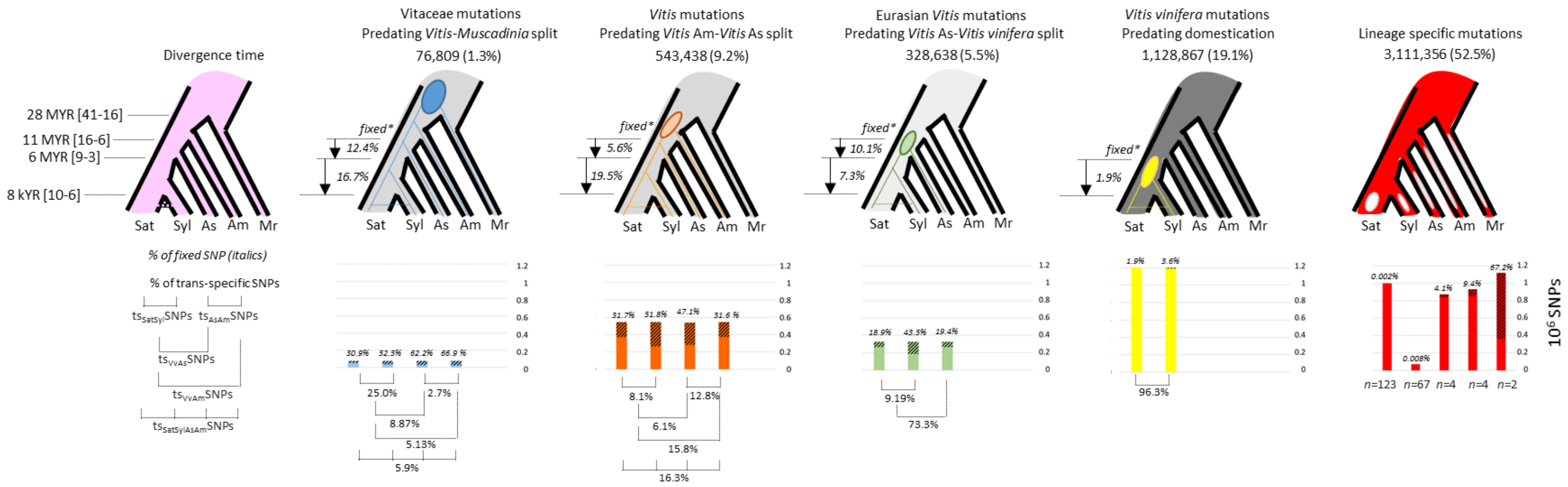

Supplementary Figure 4. Evolutionary age of 5,925,766 polymorphic sites in the genus *Vitis* and fixation rate in cultivated grapevines, intraspecific and interspecific wild relatives. Shaded areas in the bar charts indicate the percentage of fixed derived alleles. The percentages below the bar charts report trans–specific tsSNPs between different taxa. Divergence times as estimated according to Wan and coworkers[1]. Abbreviations: Am, American; As, Asian; Mr, *Muscadinia rotundifolia*; Sat, *sativa*; Syl, *sylvestris*; Vv, *Vitis vinifera*.

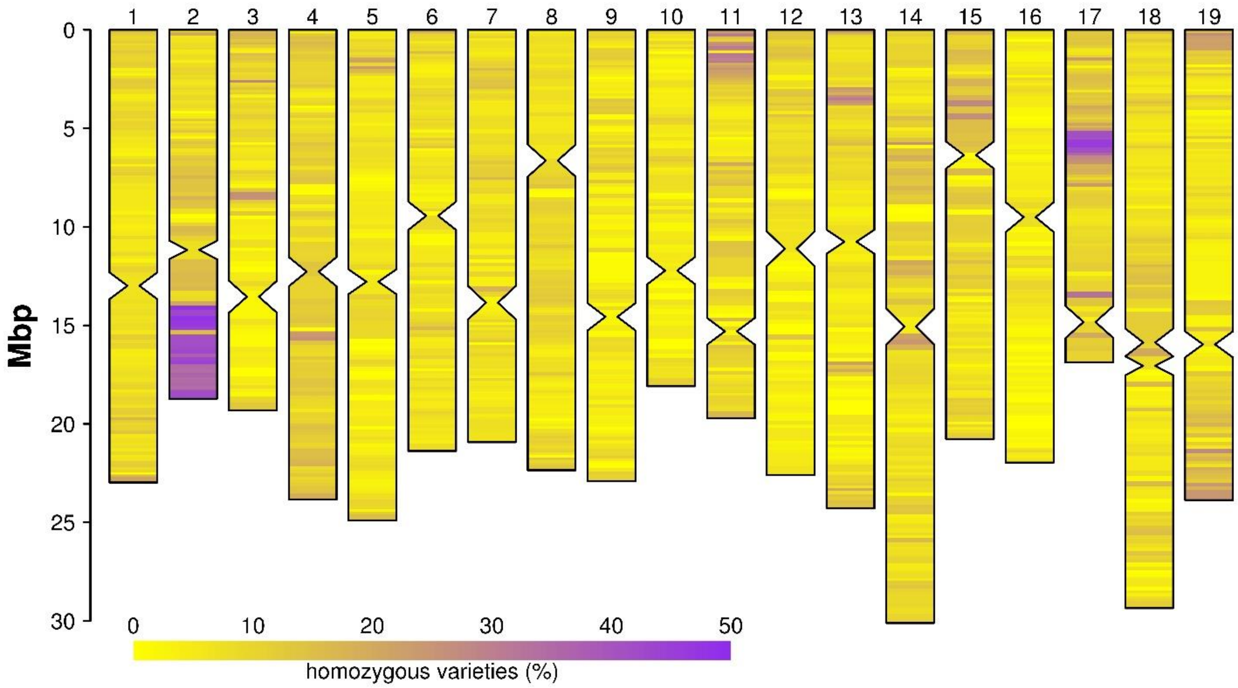

Supplementary Figure 5. Chromosomal patterns of homozygosity in cultivated varieties. The colour scale indicates the percentage of homozygous varieties in each 100–Kb window of non–repetitive DNA. Constricted regions indicate the location of centromeric repeats. Source data are provided as a Source Data file.

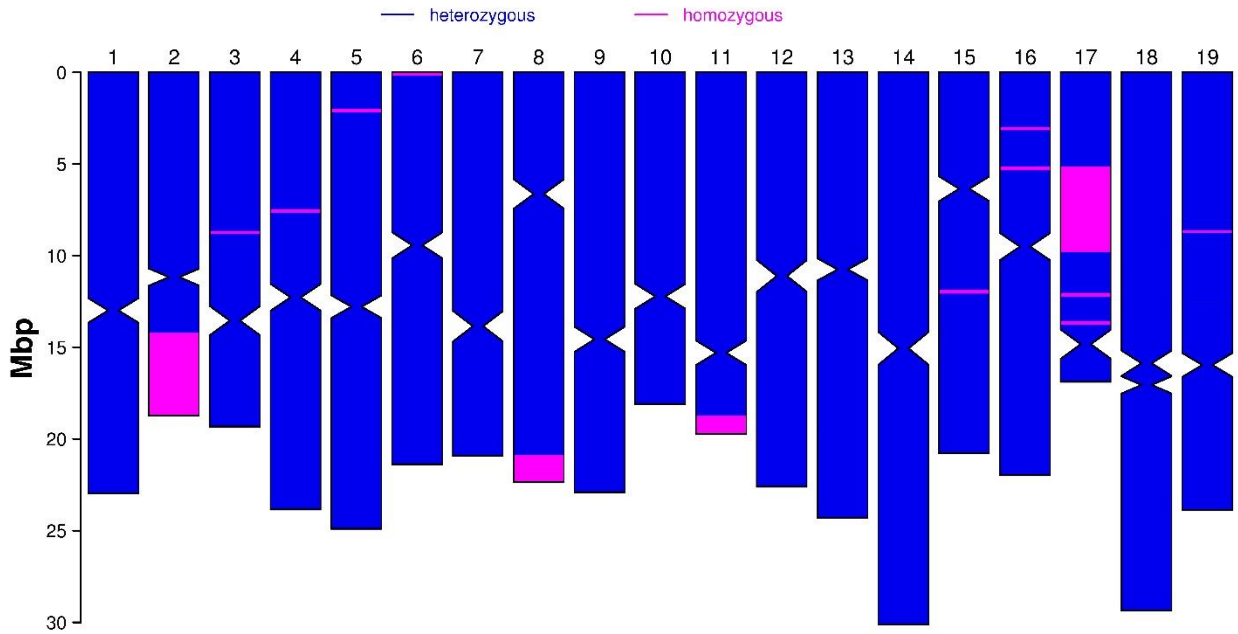

Supplementary Figure 6. Chromosomal pattern of heterozygosity in Sauvignon Blanc. Heterozygous genomic windows are indicated in blue. Homozygous genomic windows are indicated in pink. Each genomic window contains 100–Kb of non–repetitive DNA. Constricted regions indicate the location of centromeric repeats. Source data are provided as a Source Data file.

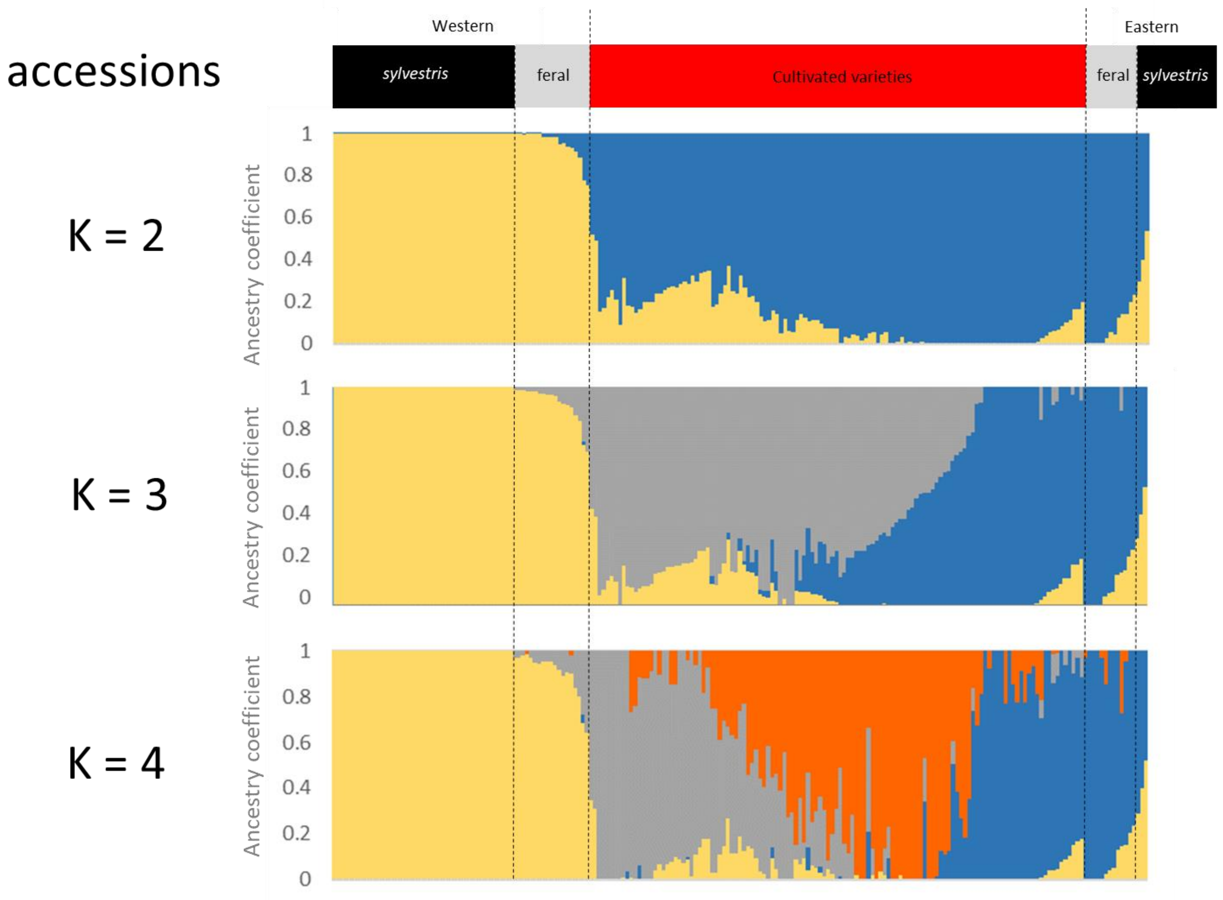

Supplementary Figure 7. Individual ancestry estimates in the species *V. vinifera* (*n* = 203). Vertical dash lines indicate the boundaries between western *sylvestris* and western feral (0.99 yellow ancestry component with K = 4), between eastern *sylvestris* and eastern feral (0.25 yellow ancestry component), and between feral and cultivated accessions.

Group composition (in the order depicted in the figure, from the left to the right):

Western *sylvestris*: TA-6267, TA-6266, TA-6265, TA-6262, TA-6258, TA-6257, TA-6256, TA-6255, TA-6254, TA-6250, TA-6248, TA-6247, TA-6244, TA-6243, TA-6242, TA-6237, TA-6236, TA-6235, TA-6234, TA-6233, TA-6230, TA-6228, TA-6224, TA-6223, TA-6222, TA-6220, TA-6219, TA-6216, TA-6212, TA-6211, TA-6210, TA-6208, TA-6207, TA-6204, TA-6199, TA-6198, TA-6194, TA-6190, TA-6187, TA-5901, TA-5607, RM02, K22, K2, TA-6263

Western feral: TA-6253, TA-5905, TA-6213, TA-6239, TA-6209, K26, K27, TA-6197, TA-6259, TA-6246, TA-6195, TA-6260, TA-6245, TA-6201, TA-6225, KE_23, GZ1, PK14, PK15

Cultivated varieties: Enantio, Lambrusco di Sorbara, Semillon, Schiava Gentile, Sauvignon Blanc, Chasselas Blanc, Helfensteiner, Schiava Grossa, Savagnin Blanc, Petit Rouge, Barbera, Berzamino, Nosiola, Gamay Noir, Chardonnay, Tocai Friulano, Refosco P.R., Picolit, Lambrusco Grasparossa, Tannat, Cabernet Sauvignon, Merlot Noir, Mauzac Blanc, Nebbiolo, Terrano, Pinot Noir, Touriga National, Raboso Piave, Greco di Tufo, Fumat, Grignolino, Corvina Veronese, Pecorino, Welschriesling, Verduzzo Friulano, Riesling Weiss, Fiano, Cabernet Franc, Moscato di Scanzo, Aglianico, Cesanese d'Affile, Falanghina, Disecka, Vermentino, Kölner Blau, Clairette Blanche, Sagrantino, Ribolla Gialla, Tibouren, Heunisch Weiss, Pinela, Malvasia Istriana, Sangiovese, Pignoletto, Malvasia di Lipari, Grechetto Bianco, Verdicchio Bianco, Graciano, Schioppettino, Montepulciano, Negro Amaro, Zelen, Muscat a

Petits Grains B., Trebbiano Toscano, Nero d'Avola, Glera, Catarratto B.C., Bombino Bianco, Uva di Troia, Italia, Vernaccia S.G., Tribidrag, Malvasia del Lazio, Ansonica, Nasco, Harslevelue, Nieddu Mannu, Garnacha, Garganega, Carignan, Gordin Verde, Mavrodaphni, Tempranillo Tinto, Muscat of Alexandria, Limnio, Kadarka, Airen, Listan Negro, Assyrtiko, Malvasia Bianca Lunga, Red Globe, Daphnia, Chaouch Blanc, Coarna Alba, Plechistik, Autumn Royal, Henab Turki, Marandi Shemakhinskii, Terbash, Taifi Rozovyi, Tagobi, Sultanina, Kandahari Siah, Narma, Kishmish Vatkana, Tebrizi, Gyulyabi Dagestanskii, Bayan Shirei, Ararati, Agadai, Tavkveri, Khop Khalat, Asyl Kara, Sirgula, Gorula, Sciavtsitska, Rkatsiteli, Alexandroouli, Tschvediansis Tetra, Mtsvane Kachuri, Ojaleshi, Mgaloblishvili, Adjaruli Tetri
Eastern feral: Pakistan3, Pakistan1, V410, V294, V292, V385, Azerbaijan2, Azerbaijan1, Turkmenistan2, V267, V389, V278, V411
Western *sylvestris*: armenia, georgia, V395
Source data are provided as a Source Data file.

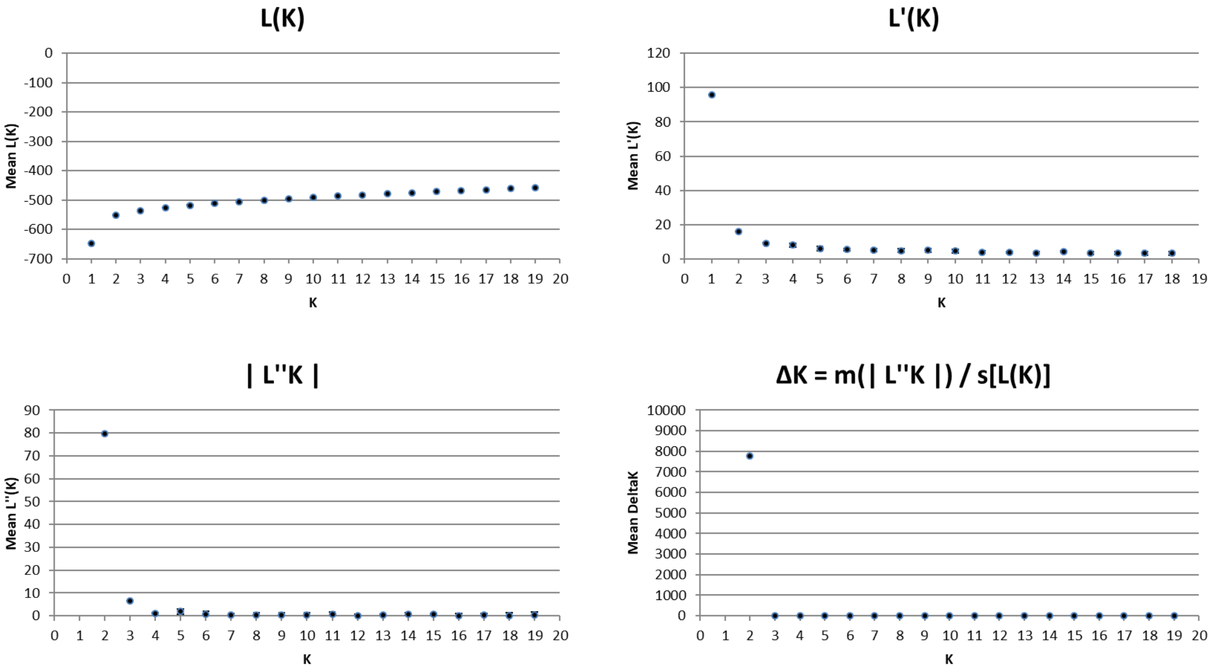

Supplementary Figure 8. ΔK plot of the Evanno's test based on ADMIXTURE analysis in the species germplasm (*n* = 203). Values in *y*–axis are reported in millions.

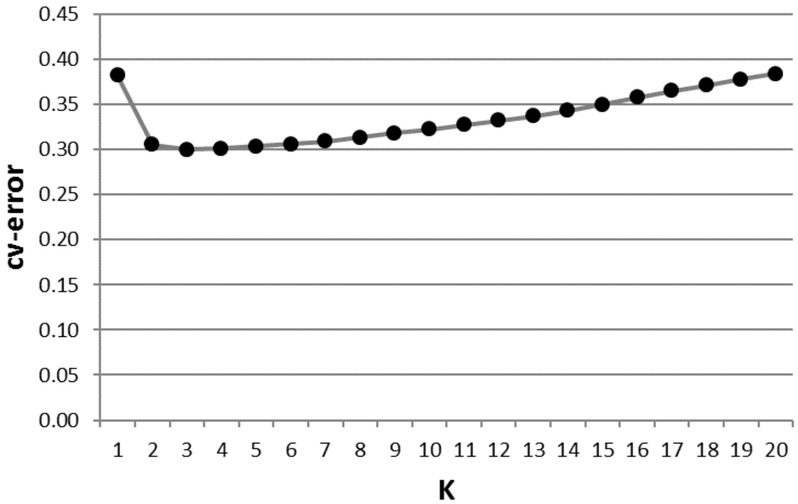

Supplementary Figure 9. Cross–validation error (cv–error) plot of K ancestry components in the species germplasm (*n* = 203). The mean cv–error value was calculated using 20 independent runs. Each run was performed using a random generated seed.

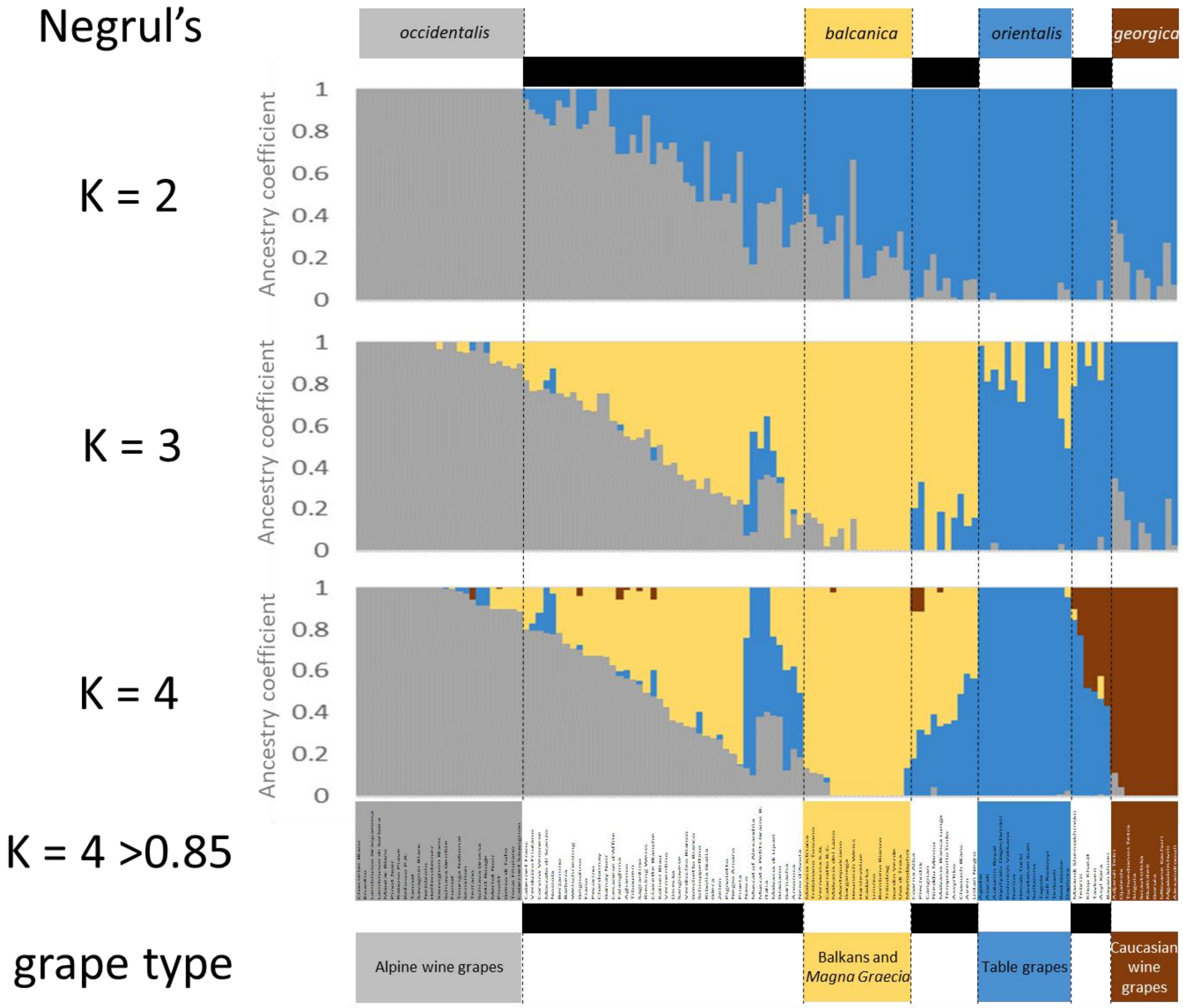

Supplementary Figure 10. Individual ancestry estimates in the cultivated germplasm (*n* = 123). Vertical dashed lines indicate the boundaries between >0.85 and <0.85 membership proportion with K = 4. Groups including varieties with >0.85 membership proportion were associated with Negrul's ecogeographical groups and with grape types.

K = 4 group composition (in the order depicted in the figure, from the left to the right):

occidentalis: Chasselas Blanc, Enantio, Lambrusco Grasparossa, Lambrusco di Sorbara, Mauzac Blanc, Pinot Noir, Raboso Piave, Refosco P.R., Tannat, Savagnin Blanc, Nebbiolo, Helfensteiner, Sauvignon Blanc, Schiava Gentile, Fumat, Touriga National, Semillon, Terrano, Schiava Grossa, Petit Rouge, Merlot Noir, Picolit, Greco di Tufo, Tocai Friulano, Cabernet Sauvignon

balcanica: Malvasia Istriana, Trebbiano Toscano, Vernaccia S.G., Catarratto B.C., Malvasia del Lazio, Montepulciano, Garganega, Heunisch Weiss, Harslevelue, Kadarka, Limnio, Bombino Bianco, Tribidrag, Gordin Verde, Uva di Troia, Mavrodaphni

orientalis: Agadai, Ararati, Autumn Royal, Gyulyabi Dagestanskii, Kishmish Vatkana, Narma, Henab Turki, Kandahari Siah, Sultanina, Tagobi, Taifi Rozovyi, Terbash, Red Globe, Daphnia

georgica: Adjaruli Tetri, Ojaleshi, Tschvediansis Tetra, Sirgula, Sciavtsitska, Rkatsiteli, Gorula, Mtsvane Kachuri, Mgaloblishvili, Alexandroouli

Admixed varieties (in the order depicted in the figure):

occidentalis / *balcanica* / *orientalis*: Cabernet Franc, Verduzzo Friulano, Corvina Veronese, Moscato di Scanzo, Nosiola, Berzamino, Barbera, Welschriesling, Grignolino, Fiano, Pecorino, Chardonnay, Gamay Noir, Cesanese d'Affile, Falanghina, Aglianico, Tibouren, Sagrantino, Riesling Weiss, Clairette Blanche, Kölner Blau, Vermentino, Disecka, Sangiovese, Verdicchio Bianco, Grechetto Bianco, Schioppettino, Ribolla Gialla, Glera, Zelen, Pignoletto, Negro Amaro, Pinela, Nasco, Muscat of Alexandria, Muscat a Petits Grains B., Italia, Malvasia di Lipari, Graciano, Garnacha, Ansonica, Nero d'Avola
balcanica / *orientalis*: Coarna Alba, Plechistik, Carignan, Nieddu Mannu, Malvasia Bianca Lunga, Tempranillo Tinto, Assyrtiko, Chaouch Blanc, Airen, Listan Negro
orientalis / *georgica*: Marandi Shemakhinskii, Tebrizi, Khop Khalat, Tavkveri, Asyl Kara, Bayan Shirei
Source data are provided as a Source Data file.

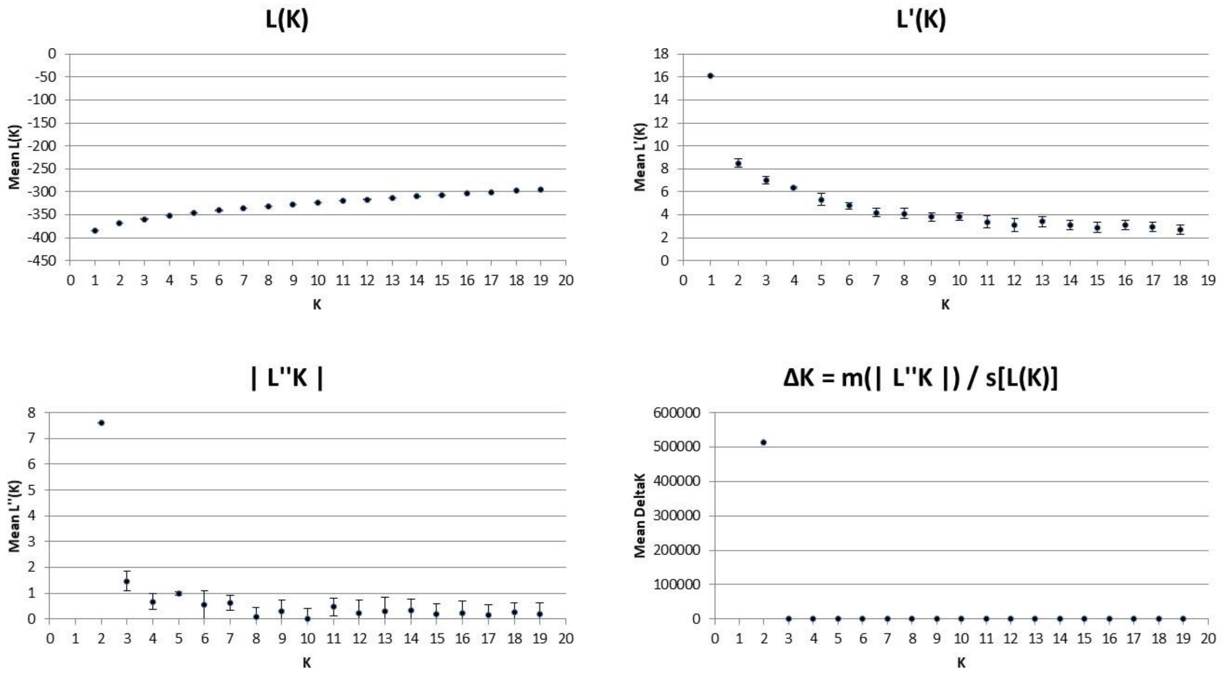

Supplementary Figure 11. ΔK plot of the Evanno's test based on ADMIXTURE analysis in the cultivated germplasm (*n* = 123).

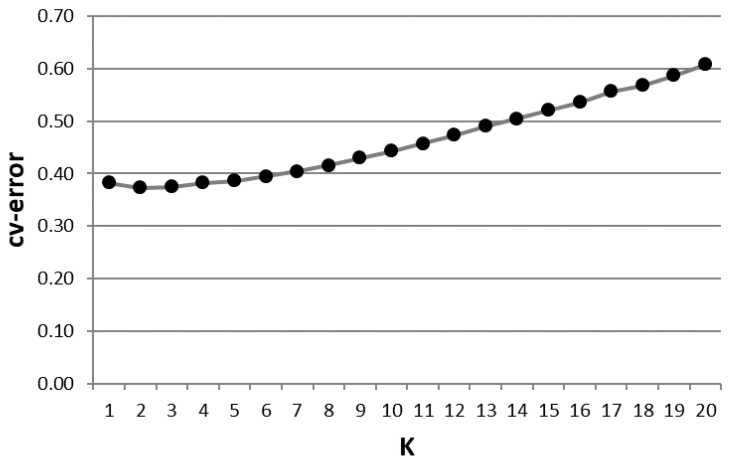

Supplementary Figure 12. Cross validation error (cv–error) plot of K ancestry components in the cultivated germplasm (*n* = 123). The mean cv–error value was calculated using 20 independent runs. Each run was performed using a random generated seed.

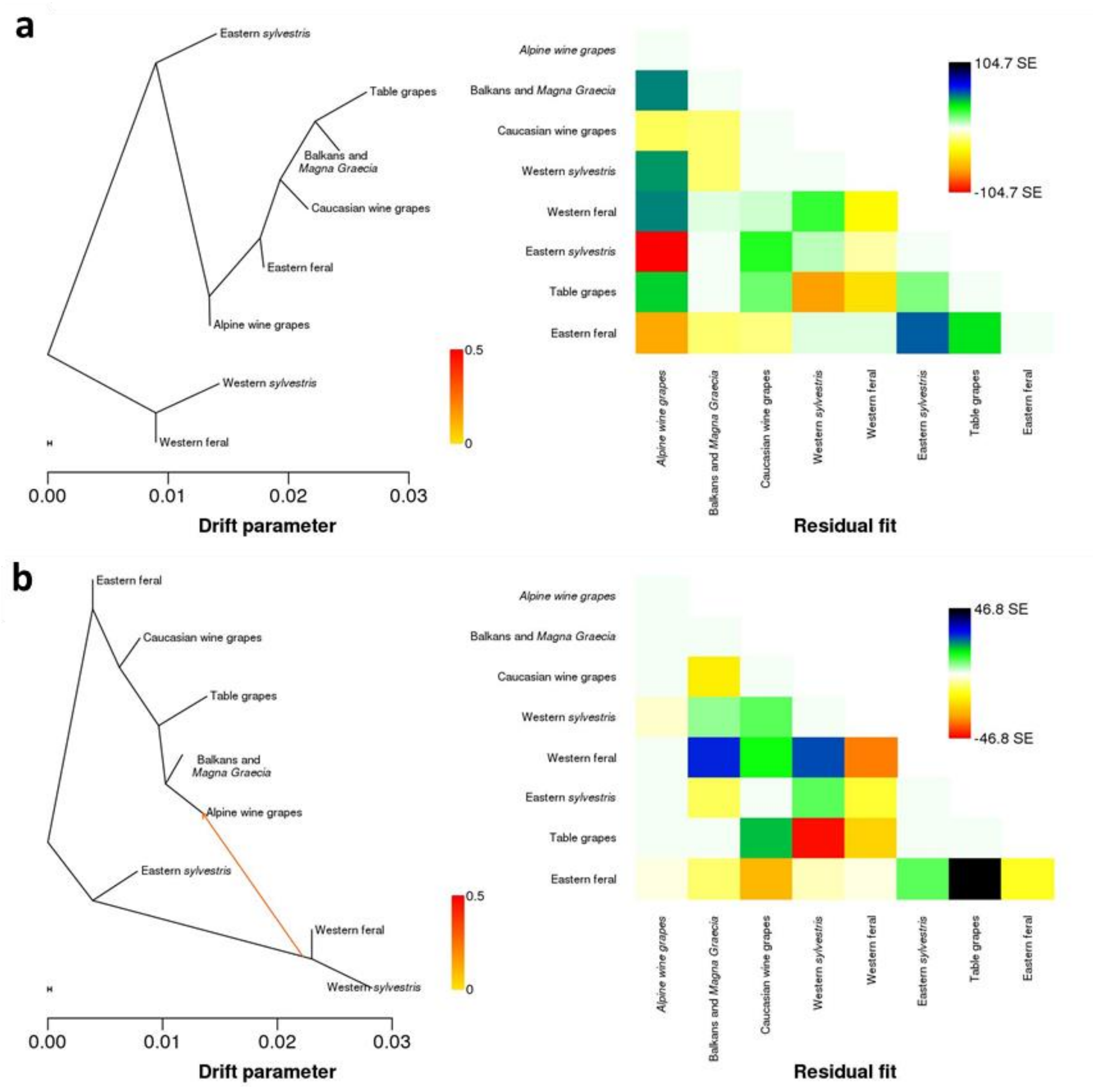

Supplementary Figure 13. Maximum likelihood bifurcating tree (a), with one migration event (b) and residual fit under the hypothesis of population structure shown in the main text. The color scale shows the migration weight. The scale bar shows ten times the average standard error of the estimated entries in the sample covariance matrix. Residuals above zero represent pairs of populations that are more closely related to each other in the data than they appear in the best–fit tree and are, therefore, candidates for admixture events. The heat map on the right shows residuals. Zero is represented by white color.

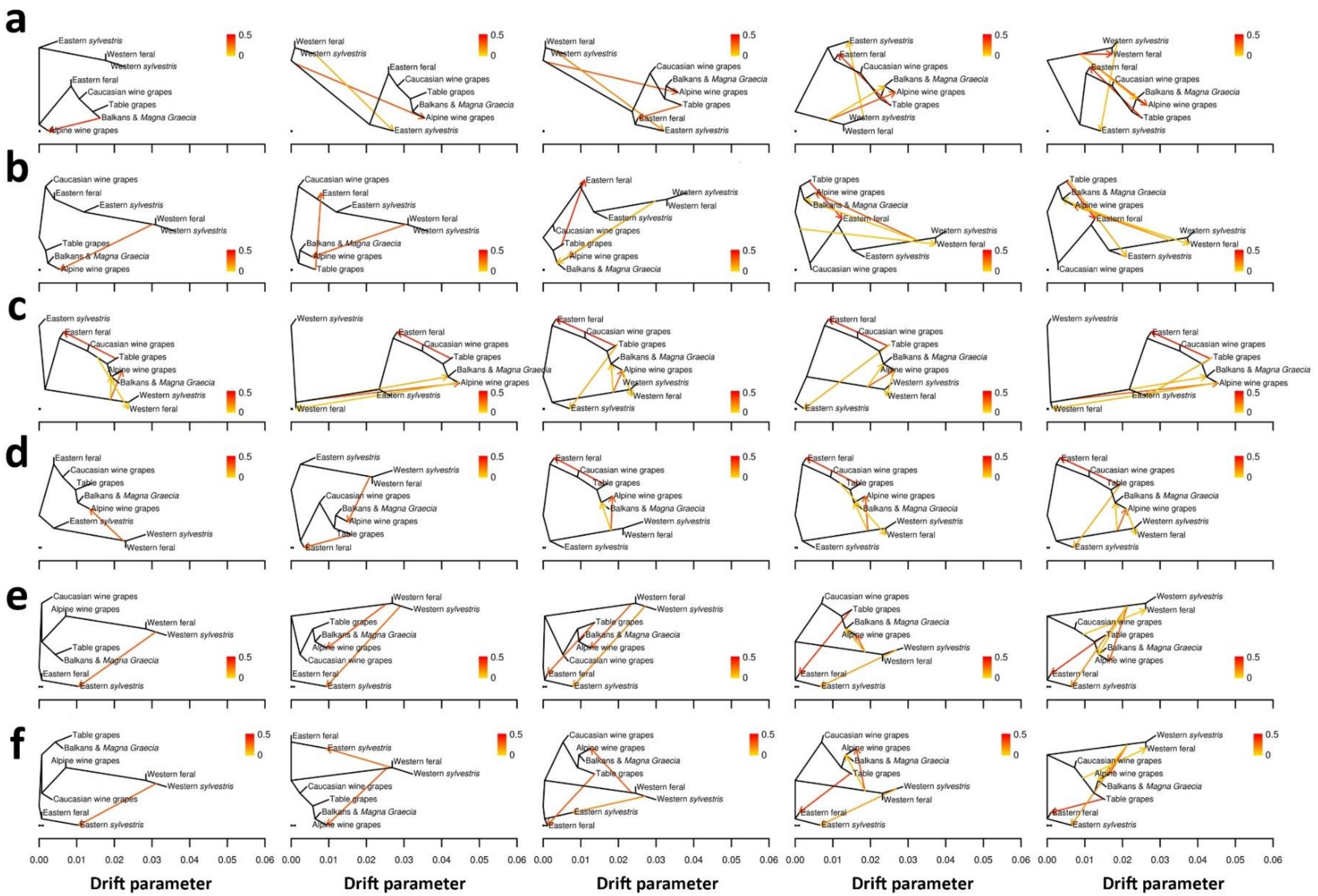

Supplementary Figure 14. Maximum likelihood trees with K = 4 ADMIXTURE groups of cultivated varieties and four groups of wild grapes with one (left) to five (right) migration events and variable windows sizes. a Window size = 100 SNPs. **b** Window size = 200 SNPs. **c** Window size = 300 SNPs. **d** Window size = 1,500 SNPs. **e** Window size = 5,000 SNPs. **f** Window size = 10,000 SNPs. The color scale shows the migration weight. The scale bar shows ten times the average standard error of the estimated entries in the sample covariance matrix. The *x*–axis scale is the same in all panels and plots. *X*–axis values are displayed only in (**f**).

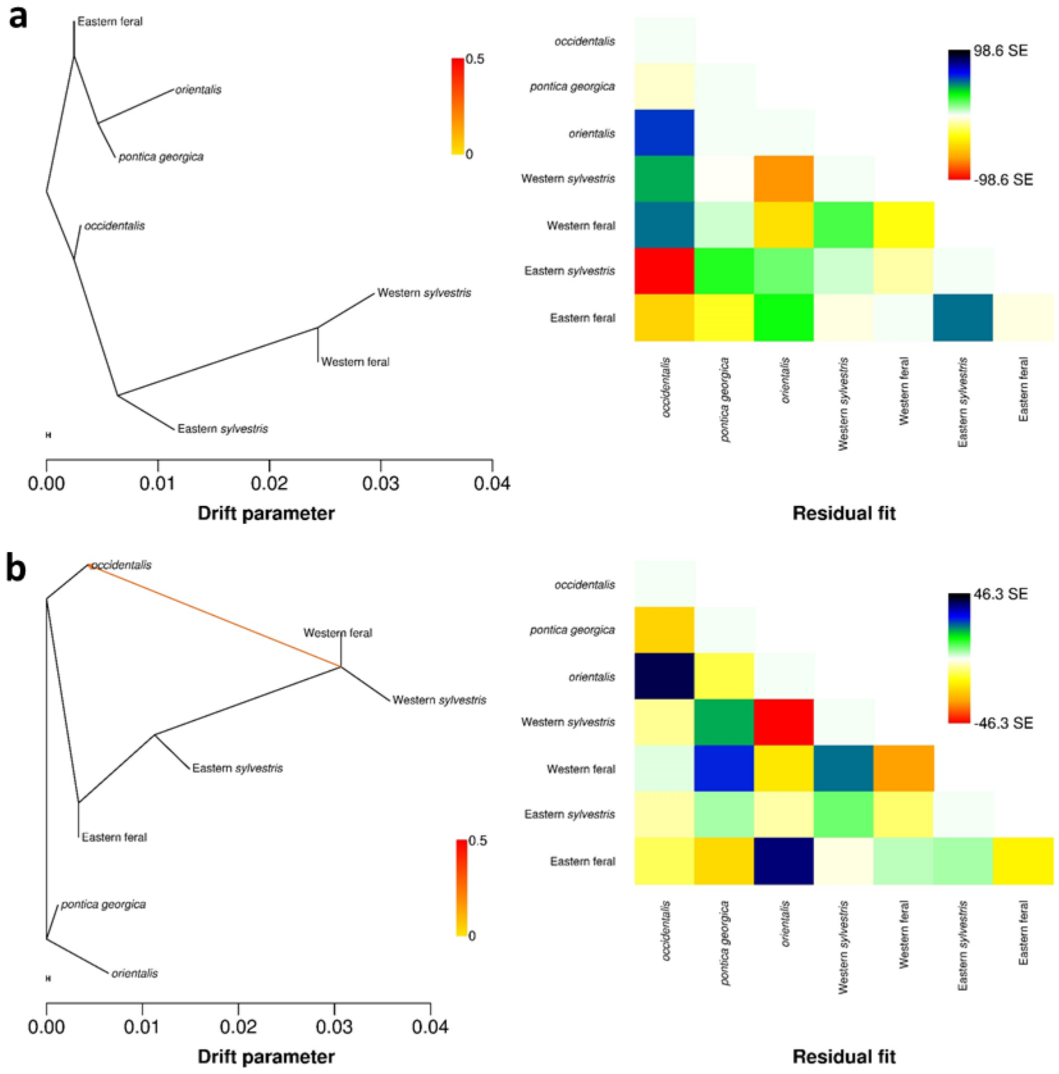

Supplementary Figure 15. Maximum likelihood bifurcating tree (a), with one migration event (b) and residual fit under a three–population scenario with (1) *pontica georgica*, (2) *orientalis*, (3) *occidentalis* ancestral populations and *pontica balcanica* discarded. The color scale shows the migration weight. The scale bar shows ten times the average standard error of the estimated entries in the sample covariance matrix. Residuals above zero represent pairs of populations that are more closely related to each other in the data than they appear in the best–fit tree and are, therefore, candidates for admixture events. The heat map on the right shows residuals. Zero is represented by white color.

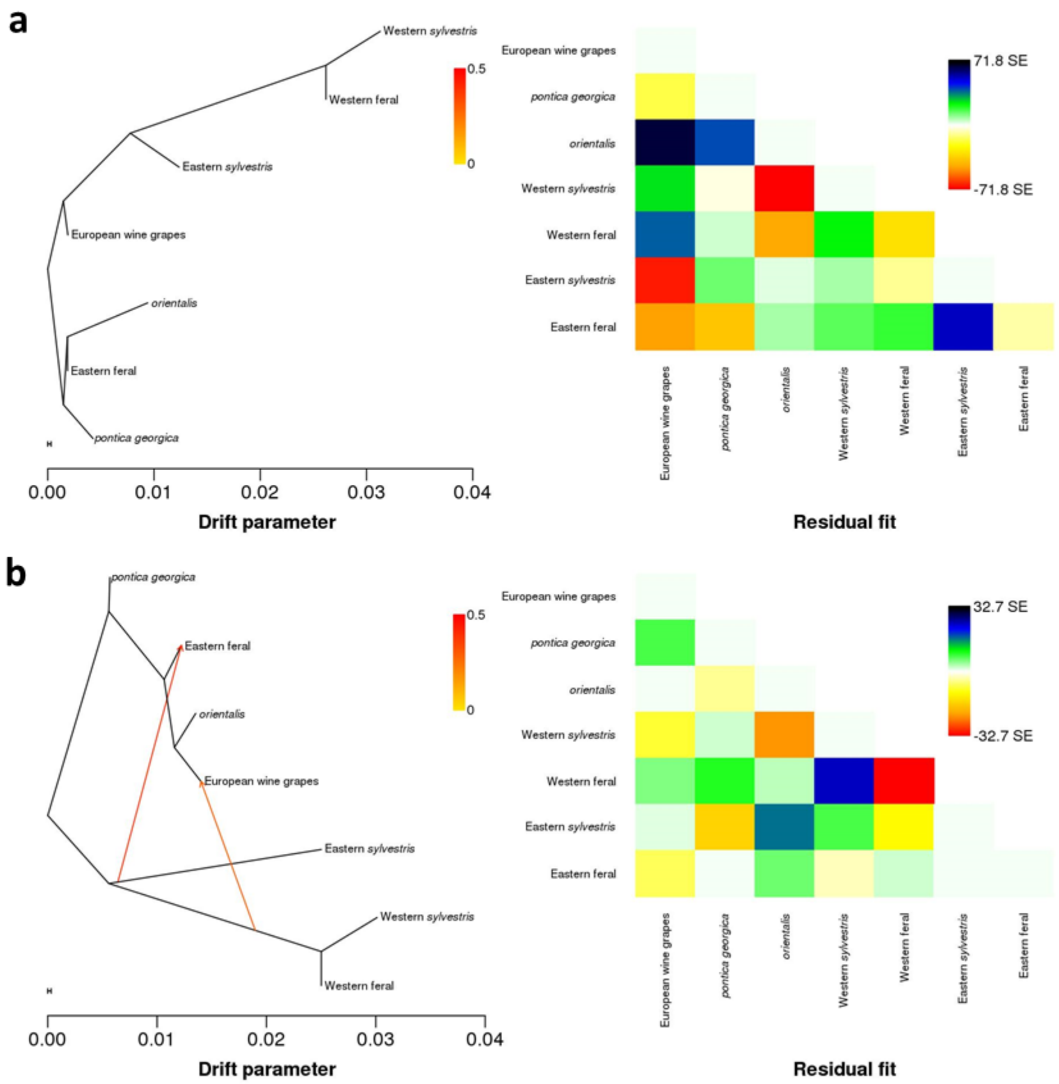

Supplementary Figure 16. Maximum likelihood bifurcating tree (a), with two migration events (b) and residual fit under a three–population scenario with (1) *pontica georgica*, (2) *orientalis*, and (3) an extended group of *occidentalis*. The color scale shows the migration weight. The scale bar shows ten times the average standard error of the estimated entries in the sample covariance matrix. Residuals above zero represent pairs of populations that are more closely related to each other in the data than they appear in the best–fit tree and are, therefore, candidates for admixture events. The heat map on the right shows residuals. Zero is represented by white color.

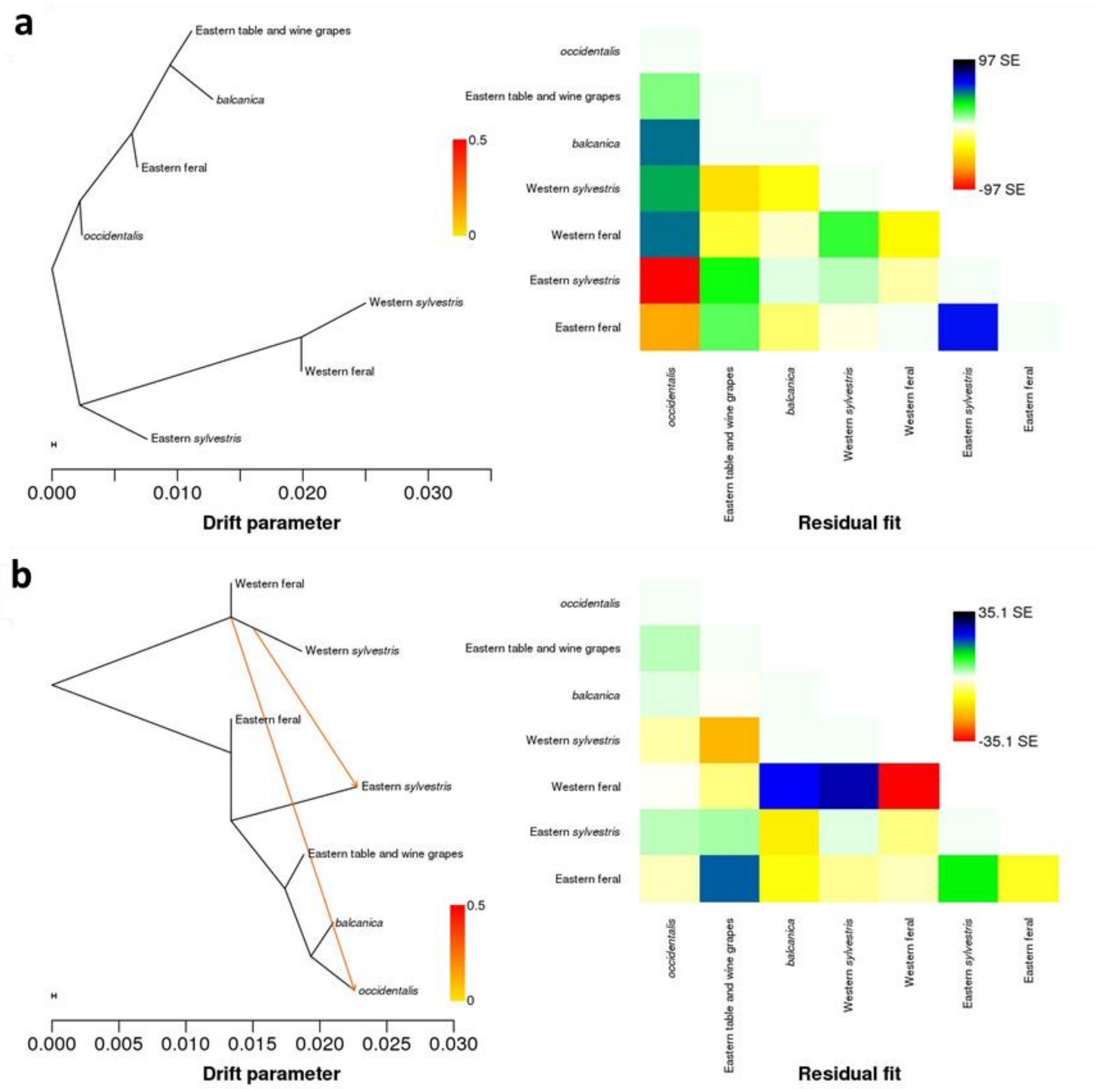

Supplementary Figure 17. Maximum likelihood bifurcating tree (a), with two migration event (b) and residual fit considering one single eastern ancestral population (including both *pontica georgica* and *orientalis*) of cultivated varieties and simulating a three–population scenario with (1) eastern diversity, (2) *balcanica* and (3) *occidentalis*. The color scale shows the migration weight. The scale bar shows ten times the average standard error of the estimated entries in the sample covariance matrix. Residuals above zero represent pairs of populations that are more closely related to each other in the data than they appear in the best–fit tree and are, therefore, candidates for admixture events. The heat map on the right shows residuals. Zero is represented by white color.

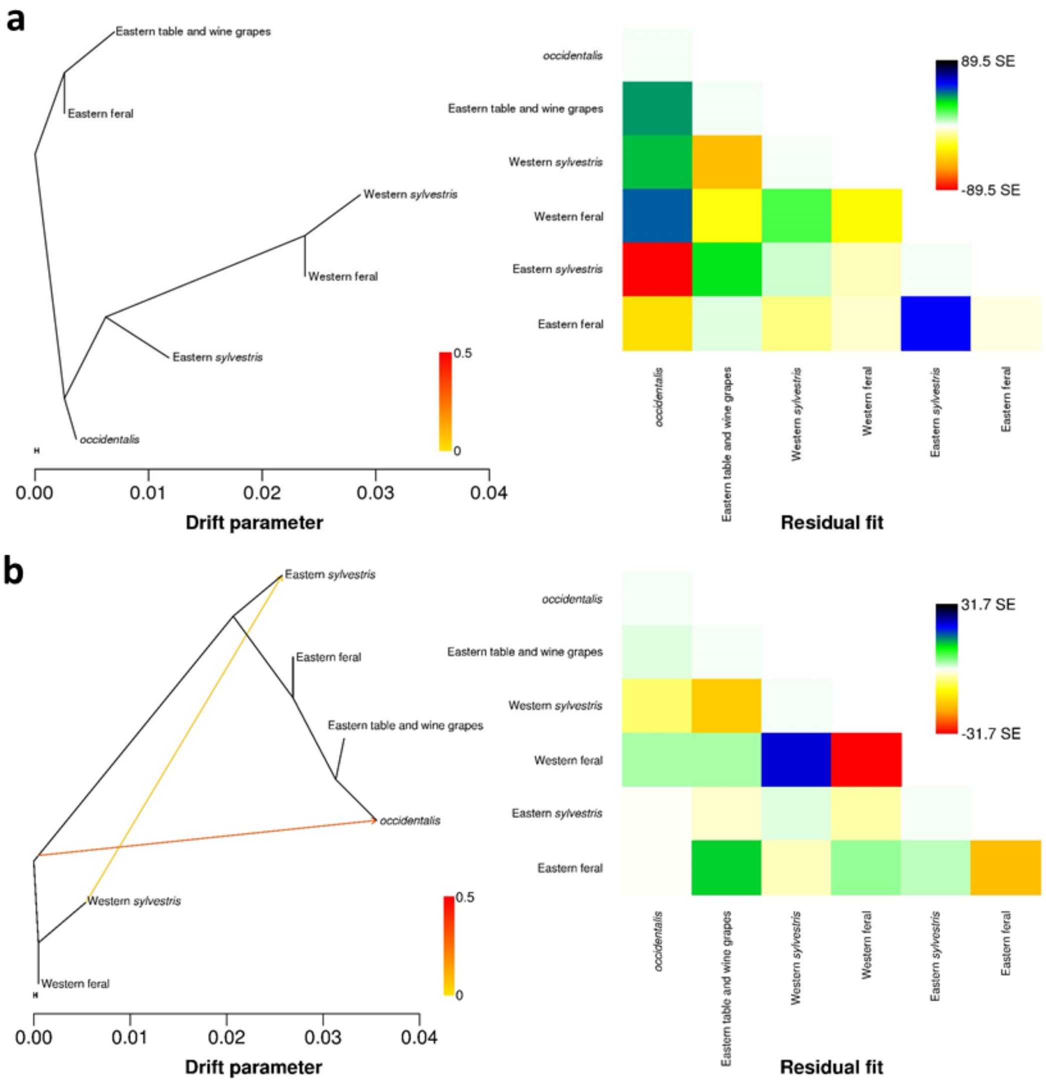

Supplementary Figure 18. Maximum likelihood bifurcating tree (a), with two migration events (b) and residual fit considering one single eastern ancestral population (including both *pontica georgica* and *orientalis*) of cultivated varieties and simulating a scenario with (1) eastern and (2) *occidentalis* ancestral populations. The color scale shows the migration weight. The scale bar shows ten times the average standard error of the estimated entries in the sample covariance matrix. Residuals above zero represent pairs of populations that are more closely related to each other in the data than they appear in the best–fit tree and are, therefore, candidates for admixture events. The heat map on the right shows residuals. Zero is represented by white color.

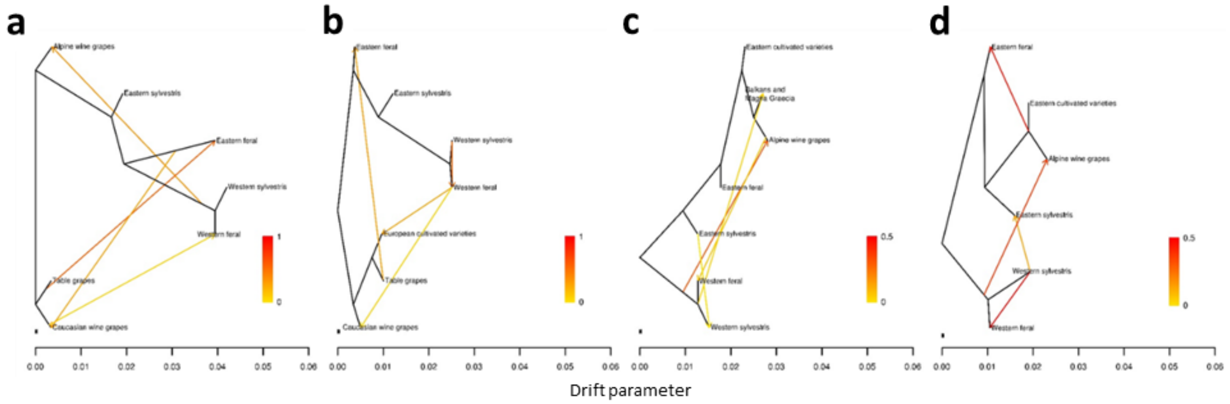

Supplementary Figure 19. Split and admixture events in populations of cultivated varieties defined by four alternative hypotheses of population structure (Supplementary Note 6), compared to the 4–population model shown in Fig. 1a and the same number of migration events. a 3–population model according to Negrul’s taxonomy treatment (*georgica* = Caucasian wine grapes, *orientalis* = Table grapes, *occidentalis* = Alpine wine grapes). **b** 3–population model (*georgica* = Caucasian wine grapes, *orientalis* = Table grapes, *occidentalis* and *balcanica* = European cultivated varieties). **c** 3–population model (*georgica* and *orientalis* = eastern cultivated varieties, *occidentalis* = Alpine wine grapes, *balcanica* = Balkans and *Magna Graecia*), according to K = 3 ADMIXTURE. **d** 2–population model (*georgica* and *orientalis* = eastern cultivated varieties, *occidentalis* = Alpine wine grapes, *balcanica* = Balkans and *Magna Graecia*), according to K = 2 ADMIXTURE. The color scale shows the migration weight. The scale bar shows ten times the average standard error of the estimated entries in the sample covariance matrix. The variance explained by the models is shown in Supplementary Fig. 20.

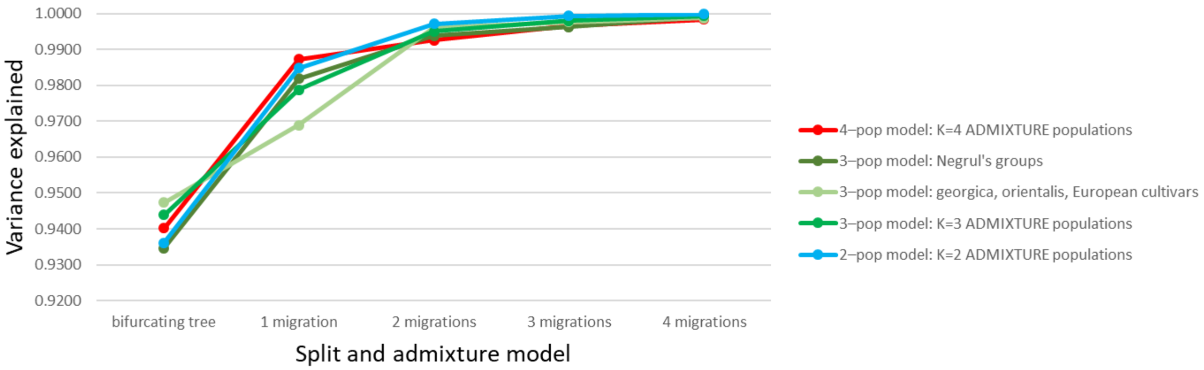

Supplementary Figure 20. Variance of relatedness among populations explained by the model with the simple bifurcating tree and with an increasing number of admixture events under alternative hypotheses of population structure. Variance is shown for the 4–population scenario presented in the main text (Fig. 1a) and for four alternative scenarios of number and type of ancestral populations discussed in Supplementary Note 6. The 4–population scenario explained the highest fraction of the variance in presence of one major admixture event.

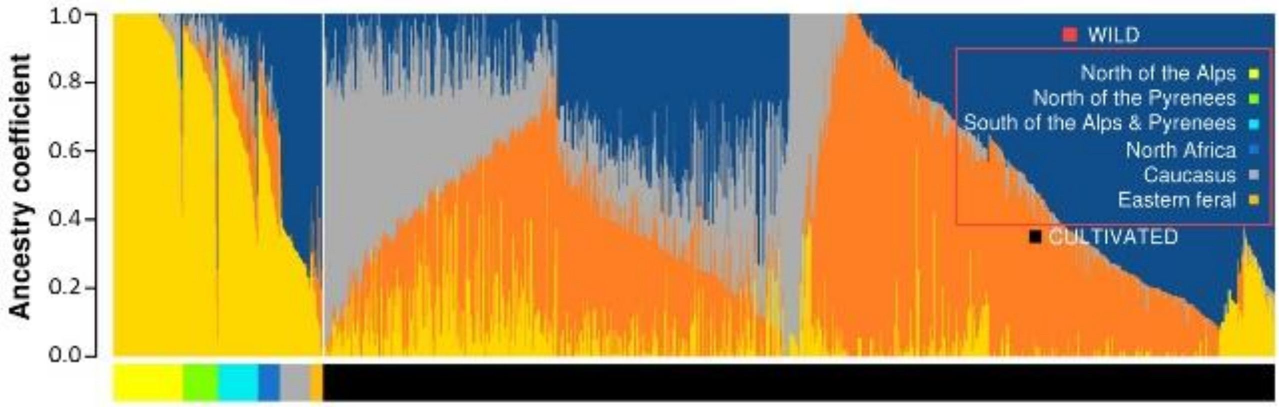

Supplementary Figure 21. Individual ancestry estimates in the diversity panel (*n* = 1,445) with K = 4 ancestry components: yellow, W1 ancestry; orange, C1 ancestry; blue, W2 ancestry; grey, C2 ancestry. SNP profiles in the wild compartment are ordered to the left–hand side of the distribution by broad geographic area of origin (North of the Alps including DEU and SVK; North of the Pyrenees standing for FRA; South of the Alps and Pyrenees including ITP and IBP; North Africa; Caucasus and Eastern Feral) and by the predominant ancestry component. SNP profiles in the cultivated compartment are ordered to the right–hand side of the distribution by the predominant ancestry component. Source data are provided as a Source Data file.

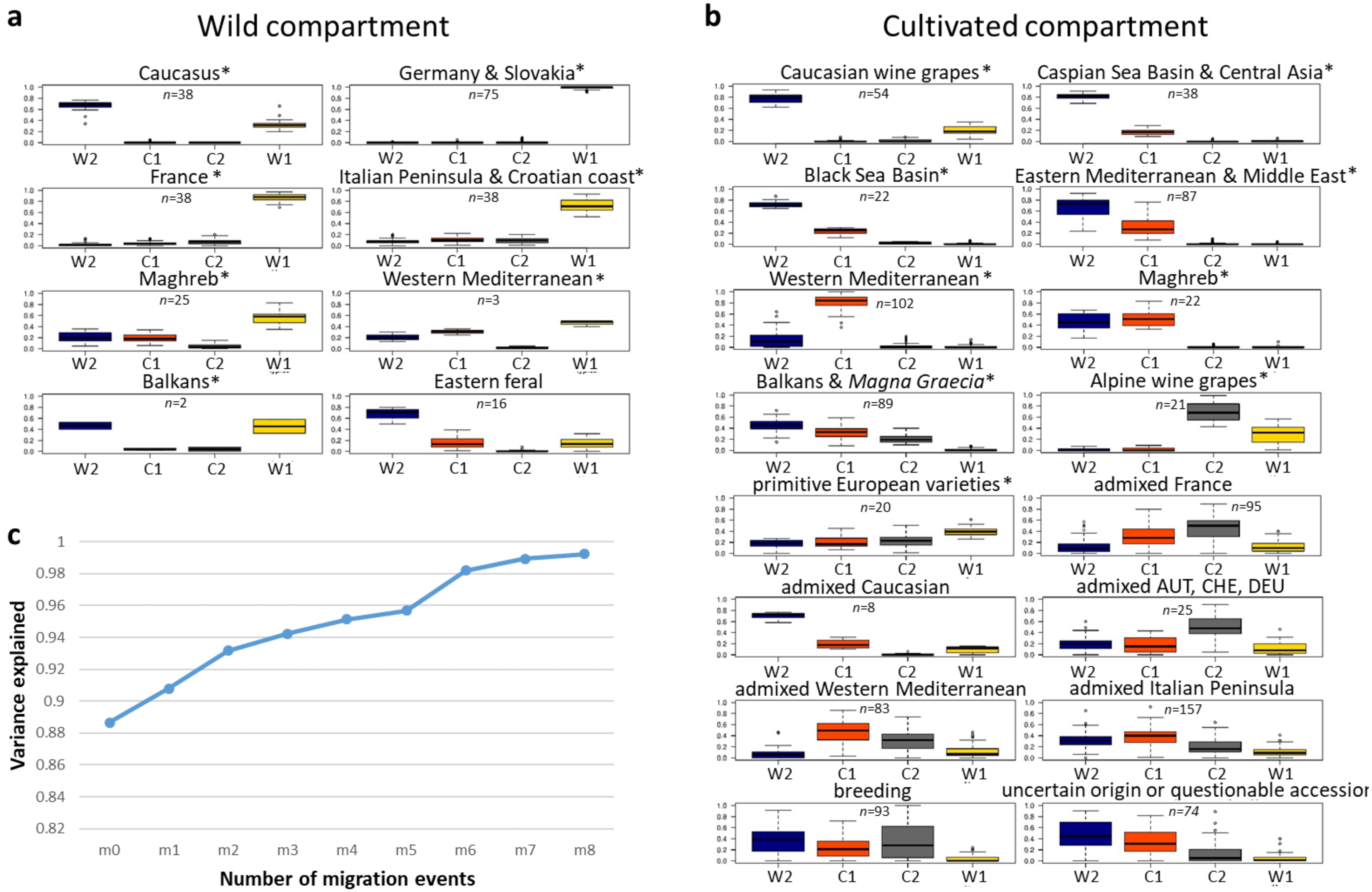

Supplementary Figure 22. Ancestry components in wild and cultivated accessions sorted by geographic area (a–b) and variance in the relatedness among groups explained by increasing numbers of migration events (c). Box plots indicate within–groups variation (**a–b**). Boxes indicate the first and third quartiles, the horizontal line within the boxes indicates the median and the

whiskers indicate ± 1.5 × interquartile range. The seven groups of wild accessions and nine well–differentiated groups of cultivated accessions that were used for TreeMix analysis in Fig. 1b and for (**c**) are marked with asterisks (**a–b**). The rise in explained variance that is caused in by the m1 and m2 migration events in (**c**) corresponds to the predicted admixture between Mediterranean lineages of *sylvestris* and introduced varieties most similar to those today grown in the Balkans and *Magna Graecia* (Fig. 1b). The m3 to m7 migration events correspond to admixture between ancestors of northern and southern populations of *sylvestris* (Fig. 1b) in areas that represented Mediterranean and Black Sea refugia for temperate trees in Europe[2]. The m8 migration that would predict admixture between northern populations of European *sylvestris* and Caucasian wine grapes is likely due to the high proportion of Caucasian *sylvestris* ancestry, which brings signatures of more ancient admixture among *sylvestris*, in Caucasian wine grapes. Source data are provided as a Source Data file.

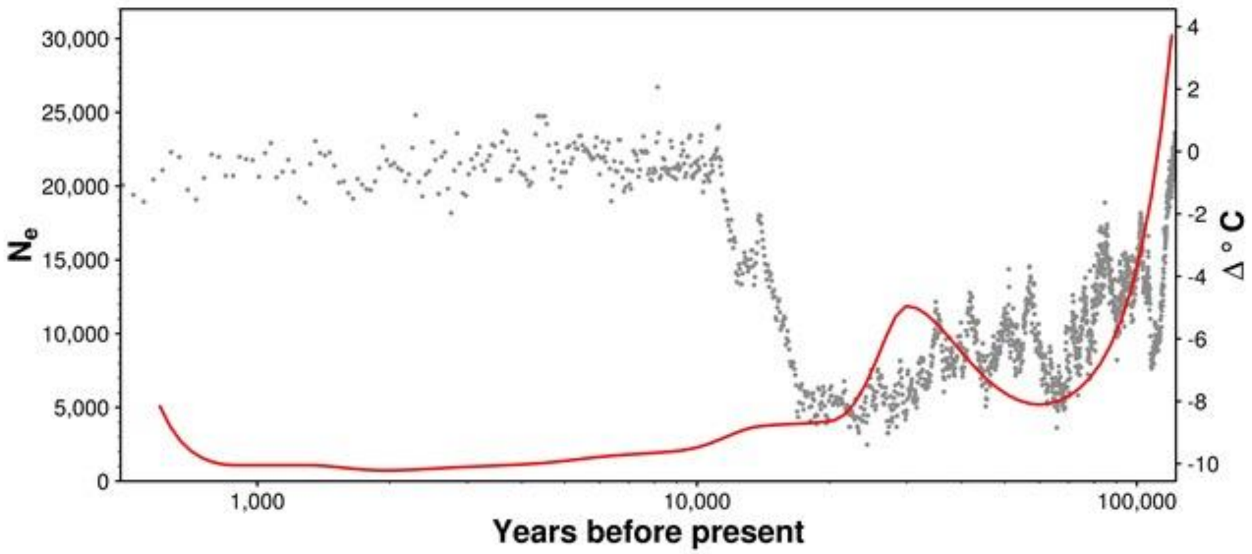

Supplementary Figure 23. Demography in the cultivated compartment. Effective population size (N_e, red line) is plotted over historical temperatures (grey dots) compared to present–day values[3]. The SMC++ software version 1.15.2[4] was used to infer demography. We used a set of unphased genotypes for intergenic mutations that occurred in the *sativa* lineage (see Supplementary Figs. 1 and 4 for the evolutionary age of mutations). The *Vcf2smc* command was used to convert diploid VCF files, with -m and -d options. The *estimate* command was used with a mutation rate of 2.5×10^{-8}, with an ending point (--tK) of 10,000 generations and --thinning option equal to 1000×log(n), where n is the haploid number for each population. The time scale was estimated assuming a generation time of 3 years.

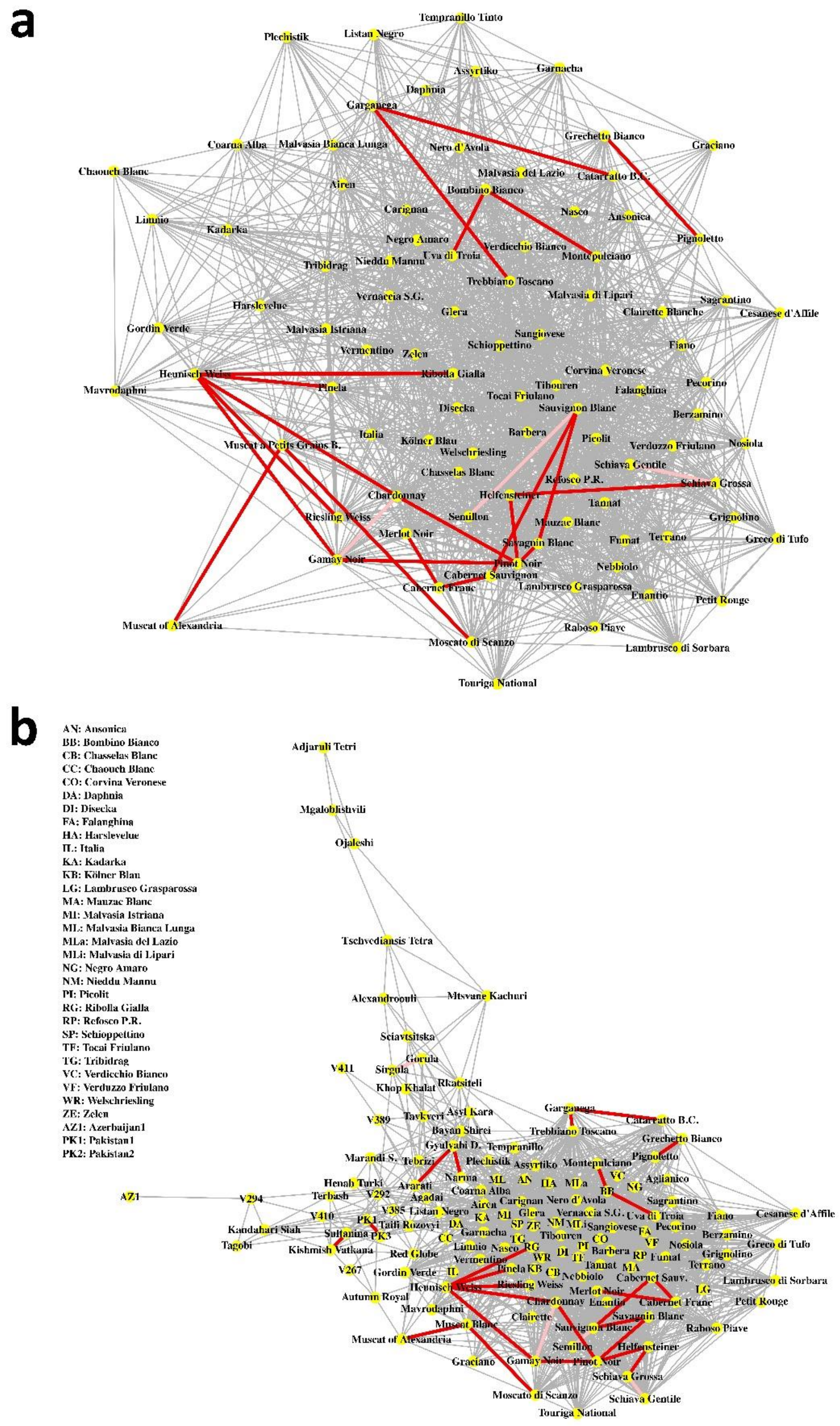

Supplementary Figure 24. Network of genealogical relationships in the WGS panel. Whole network (**a**) and magnification of the most highly interconnected part (**b**). Red connectors indicate parent–offspring relationships. Pink connectors indicate full–sibling relationships. Grey connectors indicate half–sibling, avuncular or grandparent–grandchild relationships.

Relationships were defined according to the distribution of IBD values and of IBD segment lengths shown in Supplementary Fig. 2. Adjacency matrices and network images were generated using the network package in R. Text searchable pdf files of the two panels are available at 10.6084/m9.figshare.16939465.

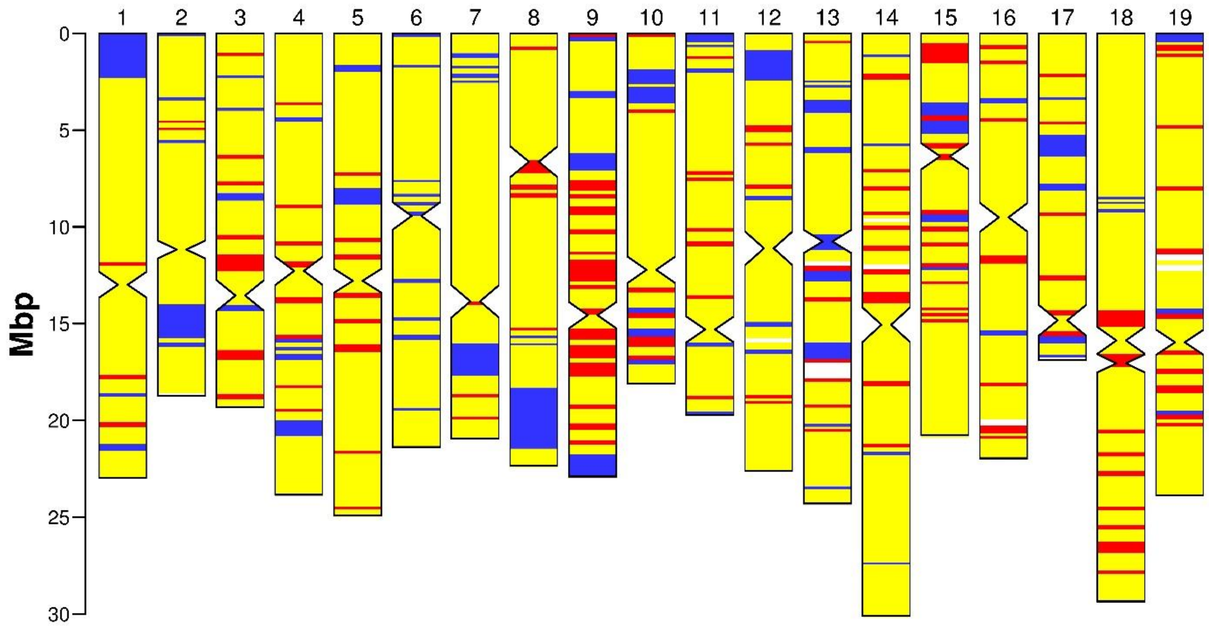

Supplementary Figure 25. Chromosomal patterns of haplotype sharing between Heunisch Weiss and Ribolla Gialla. Colors indicate the segments within each chromosome where Heunisch Weiss and Ribolla Gialla share two haplotypes (IBD=2, blue), one haplotype (IBD=1, yellow), or they appear unrelated (IBD=0, red). Each genomic window contains 100–Kb of non–repetitive DNA. Constricted regions indicate the location of centromeric repeats. Source data are provided as a Source Data file.

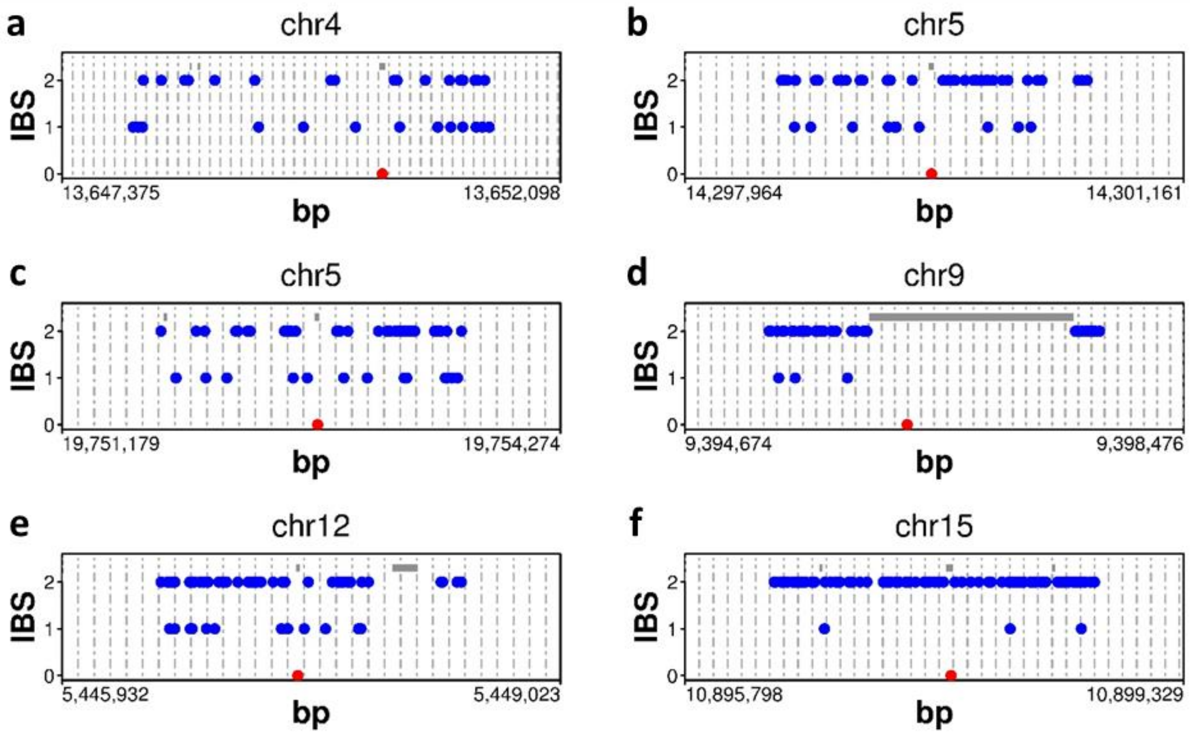

Supplementary Figure 26. IBS between Heunisch Weiss and Ribolla Gialla in six regions containing non–matching STR alleles according to De Lorenzis and coworkers[5]. STR loci VMC2B5 (**a**), VMC6E10 **(b**), VMC9B5 (**c**), VMC5C1 (**d**), VMC2H4 (**e**), VVIV67 (**f**). Blue dots indicate IBS for single nucleotide variant sites. Red dots indicate IBS for the STR loci according to De Lorenzis and coworkers[5]. Grey bars indicate repetitive DNA. Vertical dotted lines indicate 100 bp intervals.

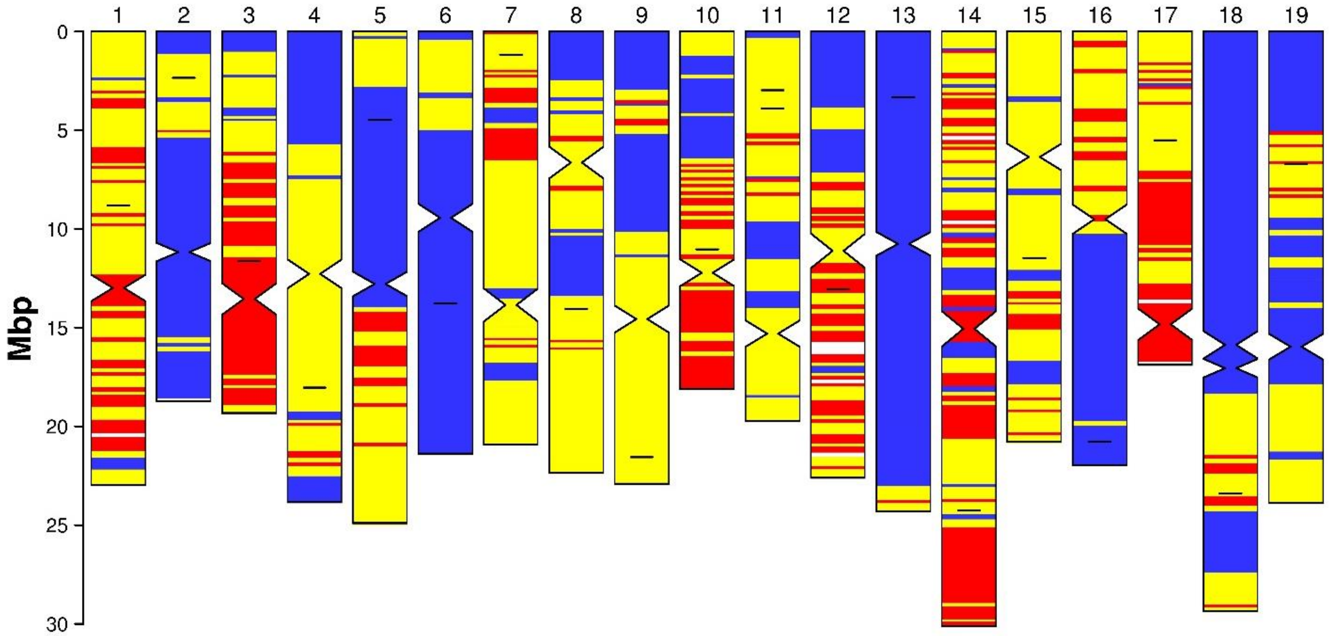

Supplementary Figure 27. Chromosomal patterns of IBD between Schiava Gentile and Schiava Grossa. Colors indicate the segments within each chromosome where Schiava Gentile and Schiava Grossa share two haplotypes (IBD=2, blue), one haplotype (IBD=1, yellow), or no haplotype (IBD=0, red). Each genomic window contains 100–Kb of non–repetitive DNA. Constricted regions indicate the location of centromeric repeats. Horizontal black ticks show the chromosomal location of 20 STRs analysed by Lacombe and coworkers[6]. Source data are provided as a Source Data file.

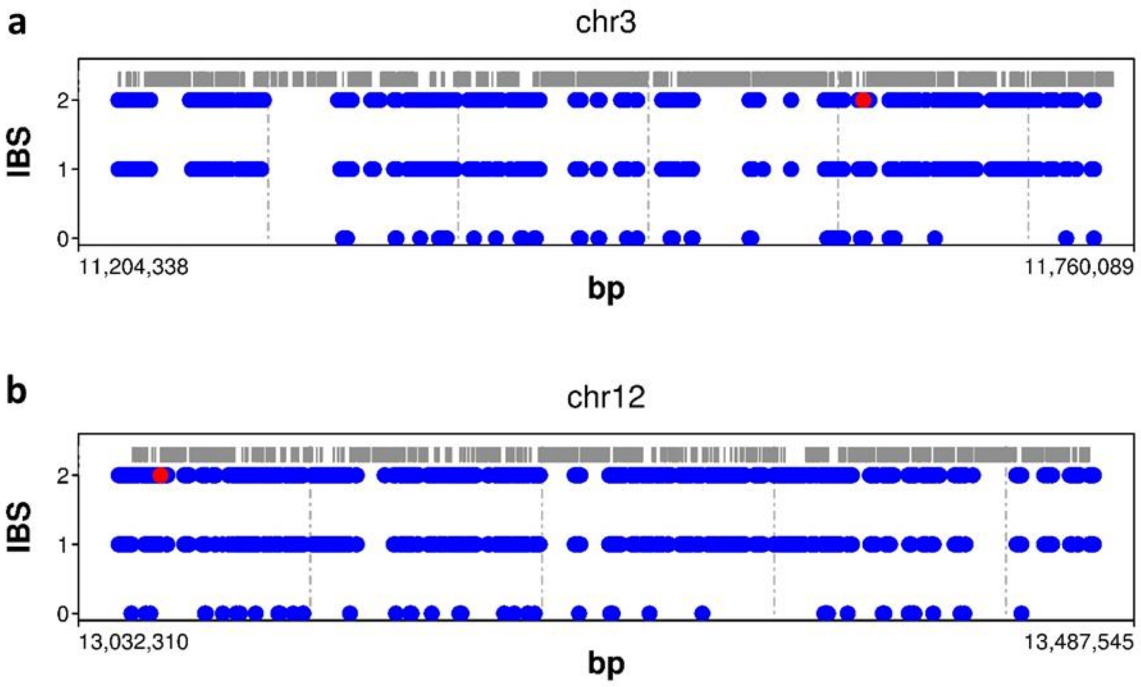

Supplementary Figure 28. IBS between Schiava Gentile and Schiava Grossa in two regions with STR homoplasy. STR loci VVMD28 (**a**) and VMC4F3–1 (**b**). Blue dots indicate IBS for single nucleotide variant sites. Red dots indicate IBS for STRs according to Lacombe and coworkers[6]. Grey bars indicate masked repetitive DNA. Grey dotted lines indicate 100 kb intervals.

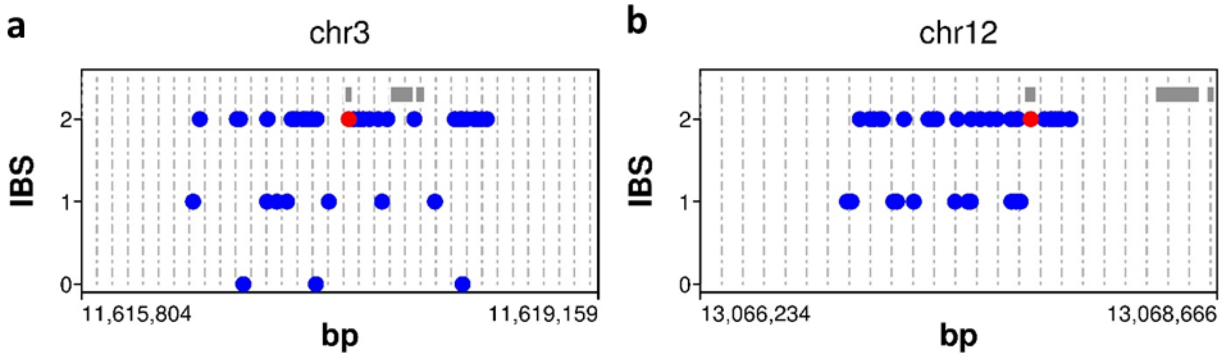

Supplementary Figure 29. IBS between Schiava Gentile and Schiava Grossa around homoplastic STRs. STR loci VVMD28 (**a**) and VMC4F3-1 (**b**). Blue dots indicate IBS for SNPs. Red dots indicate IBS for STRs according to Lacombe and coworkers[6]. Grey bars indicate masked repetitive DNA. Grey dotted lines indicate 100 bp intervals.

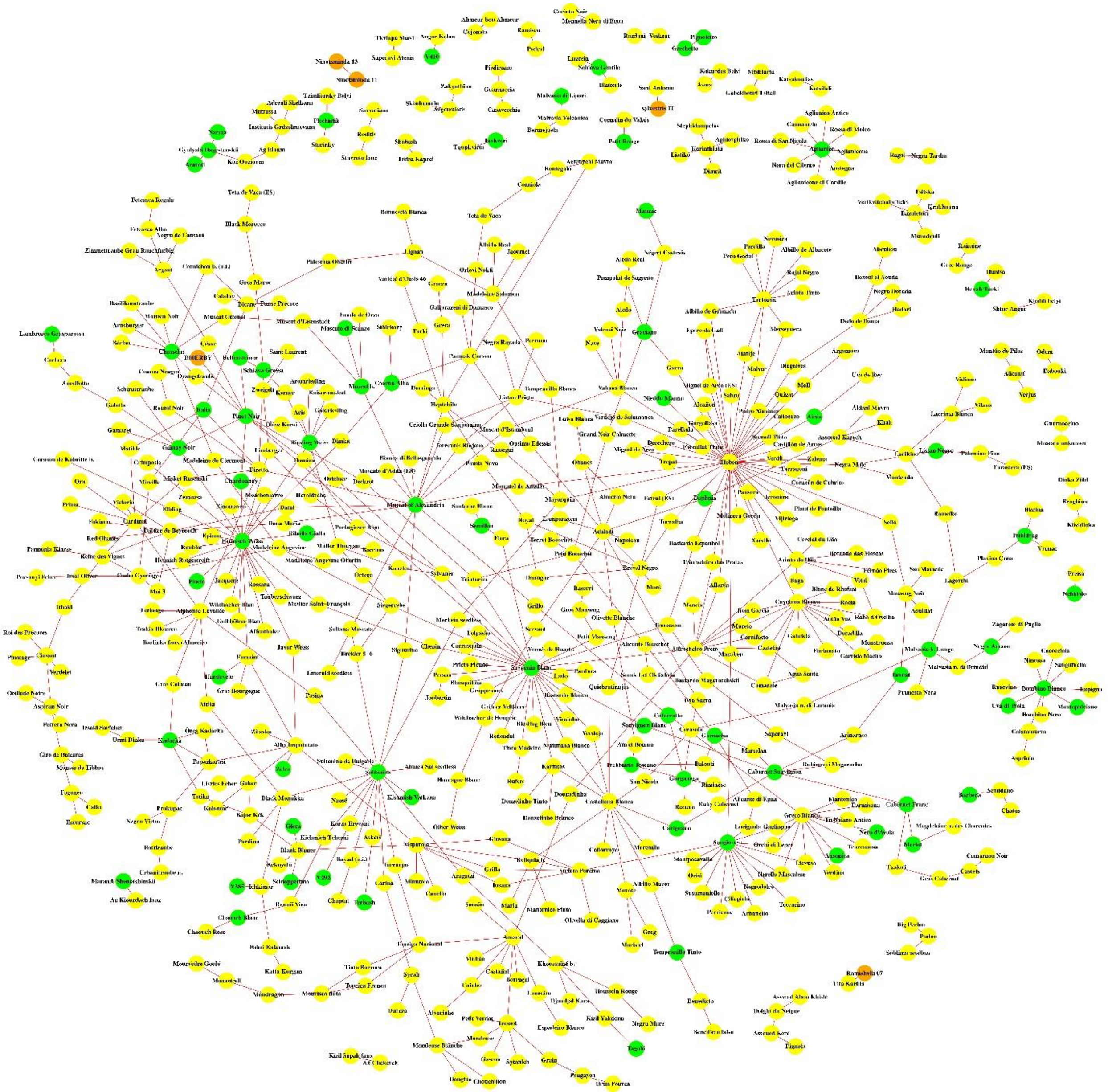

Supplementary Figure 30. Network of 657 parent–offspring relationships among 614 accessions in the diversity panel. Green vertices indicate accessions in common between the WGS and diversity panels. Orange vertices indicate feral grape accessions. Adjacency matrices and network images were generated using the network package in R. A text searchable pdf file of this graph is available at 10.6084/m9.figshare.16939465.

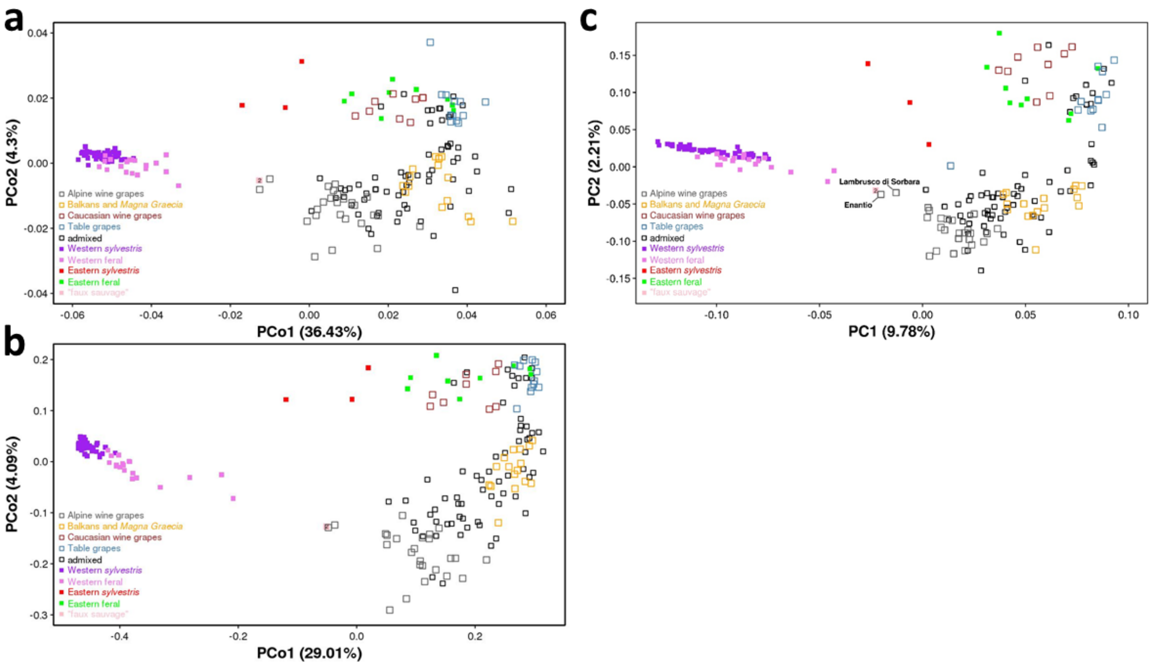

Supplementary Figure 31. Principal coordinate analysis (PCoA) based on SNPs (a–b) and principal component analysis (PCA) based on small indels (c) in the WGS panel. a PCoA based on a matrix of genotypic distance. **b** PCoA based on a matrix of haplotypic distance. **c** PCA based on small indels. The sample with uncertain assignment in the corresponding literature reports is reported as ‘faux sauvage’: 2, KE–06. Source data are provided as a Source Data file.

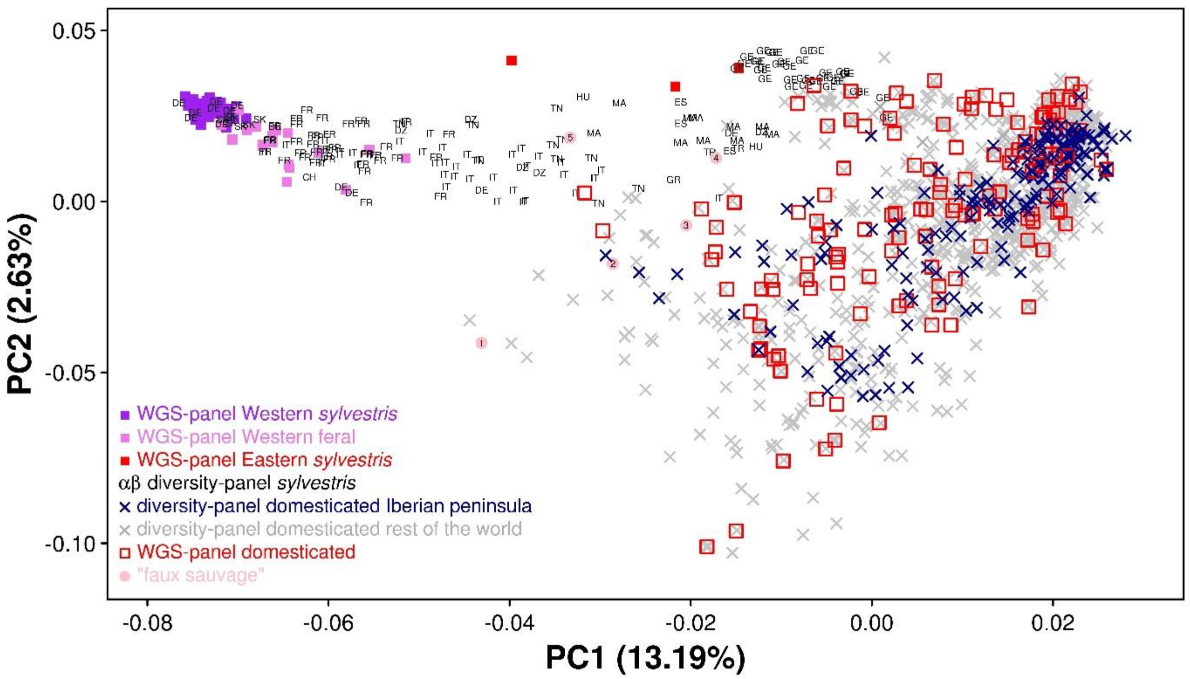

Supplementary Figure 32. Principal component analysis (PCA) in the WGS panel and in the diversity panel as in Fig. 3 illustrating the extent of genetic diversity in the Iberian cultivated germplasm captured by the WGS panel. The positions in the PCA space of 123 resequenced cultivated varieties (WGS panel, open red rectangles) and 182 Iberian cultivated varieties (blue crosses) present in the three largest European germplasm repositories (diversity panel, gray crosses) and subject to SNP–chip analysis are highlighted. Samples with uncertain assignment in their literature reports are reported as ‘faux sauvage’: 1, *sylvestris* FR B00ERBY; 2, KE–06; 3, Vigne sauvage faux ’Mouchouses 1’; 4, ‘Tighzirt 1’; 5, ‘Fethiye 58 64’. The 2–letter codes (αβ) indicate countries of origin: CH, Switzerland; DE, Germany; DZ, Algeria; ES, Spain; FR, France, GE, Georgia; GR, Greece; HU, Hungary; IT, Italy; MA, Morocco; SK, Slovakia; TN, Tunisia; TR, Turkey. The Iberian varieties that are more shifted towards lower PC1 values (< –0.02) are Castañal (B00ERD4), Alvarinho (B00ERD1), Sao Mamede (B00EQX8), Sousão (B00ERDA) with increasingly more negative PC1 values (–0.0215, –0.0235, –0.0257 and –0.0294, respectively). Source data are provided as a Source Data file.

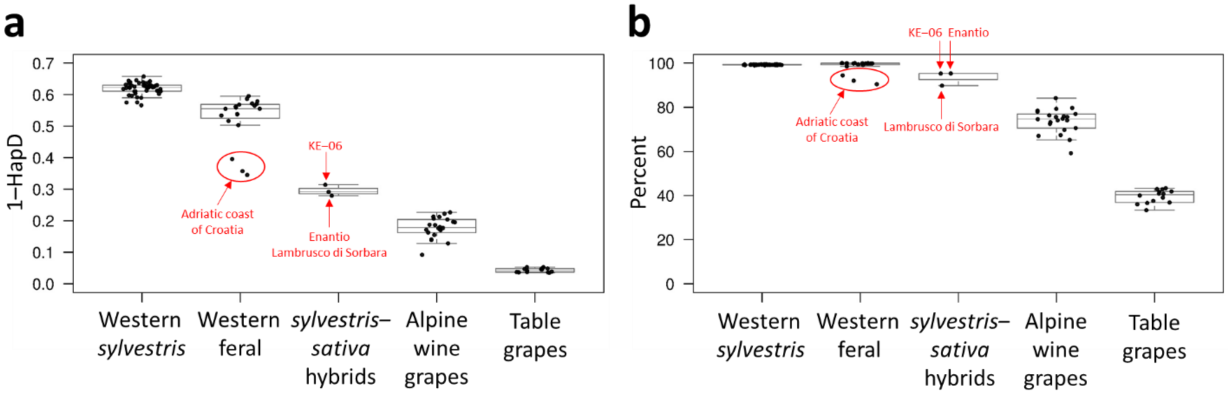

Supplementary Figure 33. Haplotype sharing between wild, feral and cultivated accessions, on one side, and the population of Western *sylvestris* on the other side. a Genome-wide estimation of average haplotype sharing with 45 accessions of Western *sylvestris* based on inverse values of haplotypic distance (1–hapD). HapD was calculated using the Equation **3**. **b** Percent of genomic windows showing identity–by–descent in one or both homologous chromosomes (IBD=1 or IBD=2) with at least one individual of the Western *sylvestris* population. In both panels, values for each individual of the Western *sylvestris* population (leftmost box) were calculated in comparisons with the remaining individuals of the same population. Alpine wine grapes and table grapes are defined as reported in the source data of Supplementary Fig. 10. Boxes indicate the first and third quartiles, the horizontal line within the boxes indicates the median and the whiskers indicate ± 1.5 × interquartile range. Source data are provided as a Source Data file.

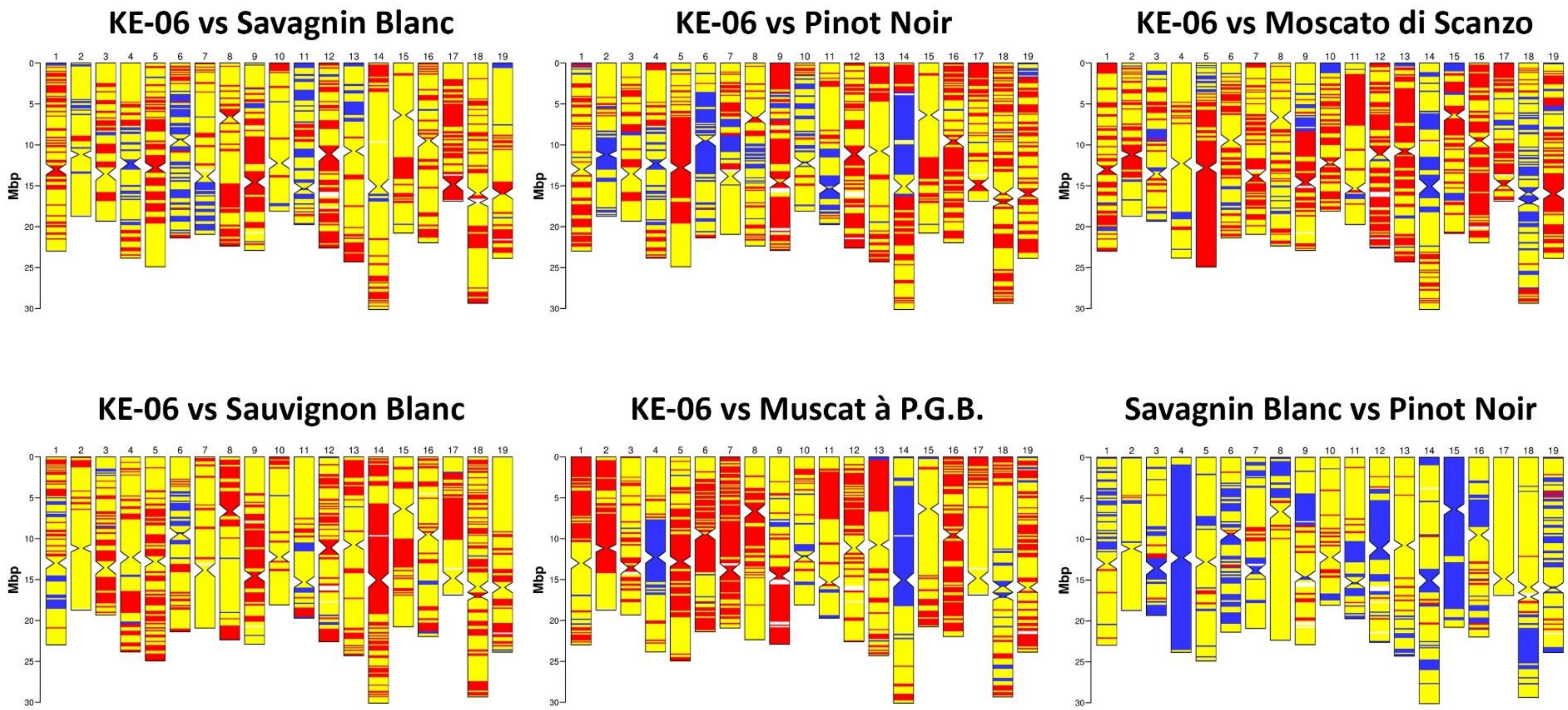

Supplementary Figure 34. Chromosomal patterns of IBD between the feral accession KE–06 and five cultivated varieties that showed the longest physical length of haplotype sharing. Haplotype sharing across the haploid set of 19 chromosomes. Colors indicate the segments within each chromosome where the pair shares two haplotypes (IBD=2, blue), one haplotype (IBD=1, yellow), or the members appear unrelated (IBD=0, red). Each genomic window contains 100–Kb of non–repetitive DNA. Constricted regions indicate the location of centromeric repeats. The comparison between Savagnin Blanc and Pinot Noir is reported as a control for a known parent–offspring relationship. Source data are provided as a Source Data file.

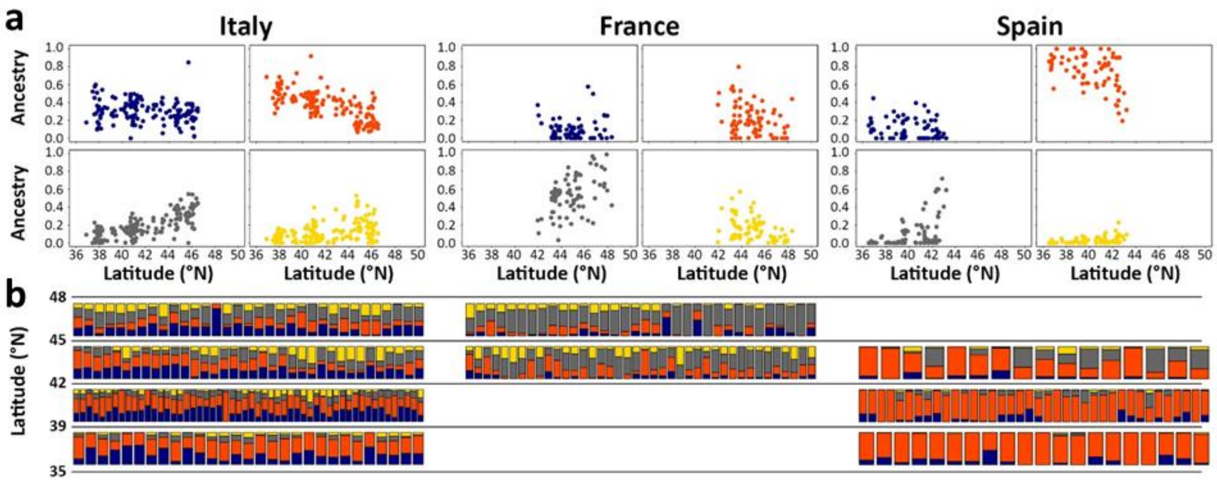

Supplementary Figure 35. Ancestry versus latitude. Correlation between ancestry components and latitude of the geographic location represented by either the most ancient known area of cultivation (for widely spread and so–called international varieties) or the most typical or renowned growing region at the present time (for locally grown varieties). Colors represent W2 ancestry (blue), C1 ancestry (orange), C2 ancestry (grey), and W1 ancestry (yellow). **a** Each ancestry component for a given variety is plotted separately in four ancestry plots. **b** Each variety is plotted as a histogram with stacked ancestry components. Varieties are grouped in four latitudinal ranges. Source data are provided as a Source Data file.

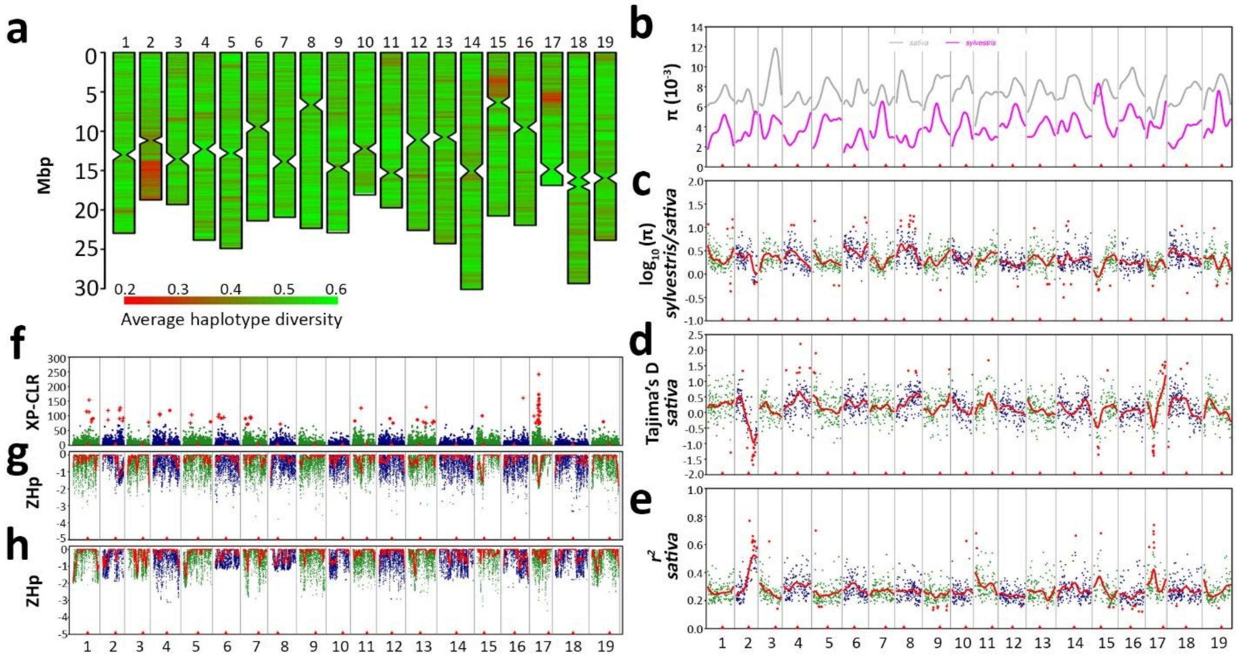

Supplementary Figure 36. Genetic variation and signals of selection across the grapevine genome. Chromosomal plots of haplotype diversity (**a**), nucleotide diversity (**b**), Tajima's *D* (**d**) and r^2 (**e**) in *sativa*. **c** Relative reduction of nucleotide diversity in *sativa* compared to *sylvestris*. Selective sweeps based on a XP–CLR test between *sativa* and *sylvestris* (**f**) and negative ZHp scores in *sativa* (**g**) and *sylvestris* (**h**). XP–CLR was calculated according to Chen and coworkers[7] in non–overlapping windows of 4 Kb using a population–scaled recombination rate of 2.5×10^{-7}. Top 0.1 % XP–CLR values in (**f**) are plotted as red dots and those also referring to windows containing more than twice the mean number of SNPs as red plus. ZHp was calculated in windows of 40 Kb with 25 % overlap using the equation proposed by Rubin and coworkers[8]. Red lines in **c**, **d**, **e**, **g** and **h** represent cubic smoothing splines of values. Red triangles indicate the location of centromeric repeats.

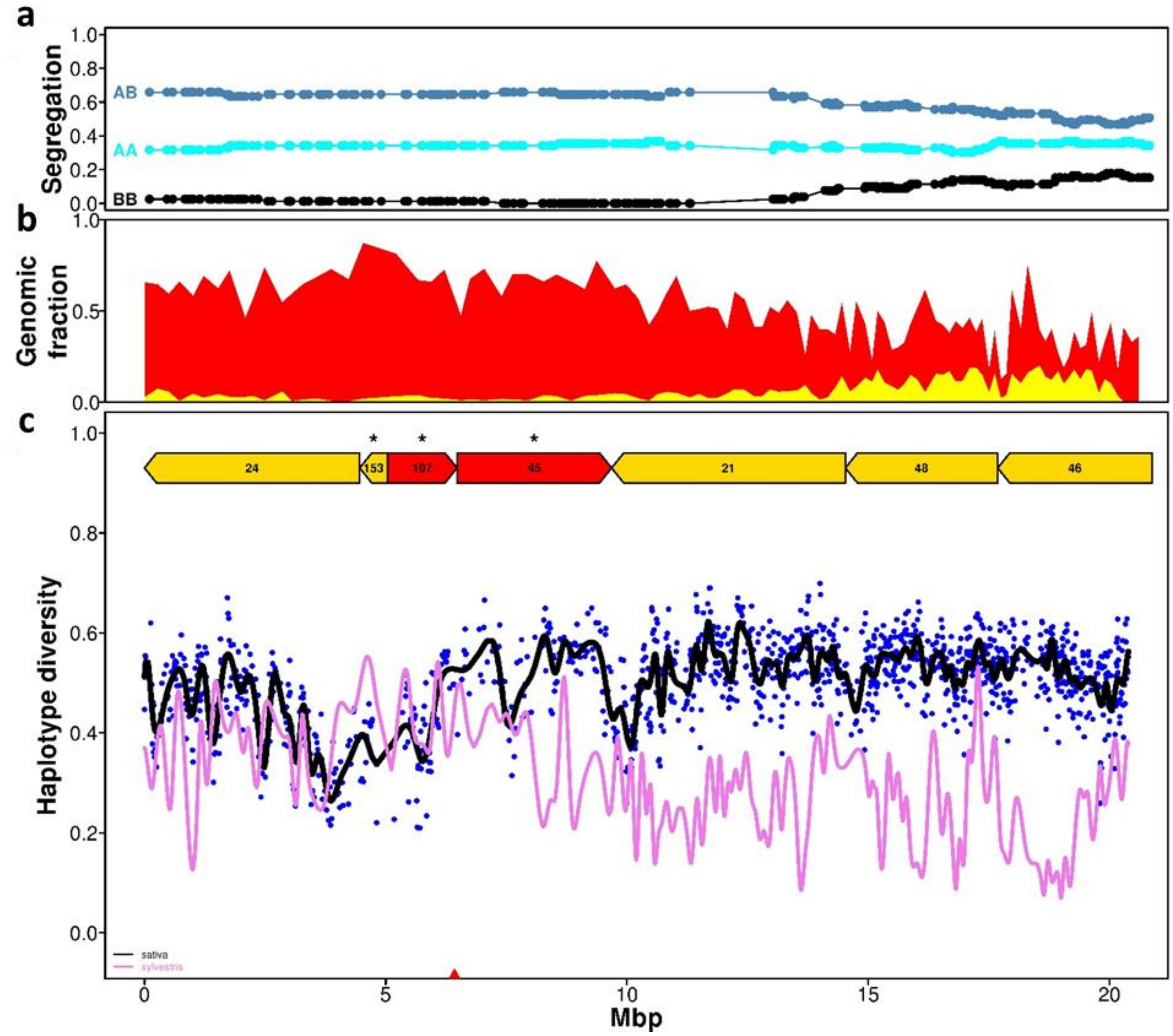

Supplementary Figure 37. Intrachromosomal pattern of haplotype diversity in *sativa* and *sylvestris* along chromosome 15 and segregation distortion in a selfed progeny of Pinot Noir. **a** Genotypic frequencies in a selfed progeny of Pinot Noir at 539 segregating sites. 'A' refers to the reference allele in Pinot Noir, 'B' to the alternate allele. **b** Genomic fractions corresponding to exons (yellow), introns and intergenic space (white), and repetitive DNA (red). **c** Haplotype diversity was calculated in blocks of five consecutive variant sites and plotted for *sativa* as the average of 50 consecutive blocks (blue dots). The black line represents a cubic smoothing spline of the data. The pink line represents a cubic smoothing spline of the data (not plotted) in *sylvestris*. The red triangle indicates the position of centromeric repeats. The diagrams above the plot represent sequence scaffolds. The asterisks indicate scaffolds that were anchored, reordered or oriented by evidence of genetic mapping in comparison to the chromosome pseudomolecule of the 12Xv0 version of the grapevine assembly. Orientation of sc_153 and sc_107 was supported by evidence of genome assemblies and PacBio long reads from genomic DNA of Merlot and Black Corinth[9]. Gold diagrams indicate scaffolds with (–) orientation. Red diagrams indicate scaffolds with (+) orientation.

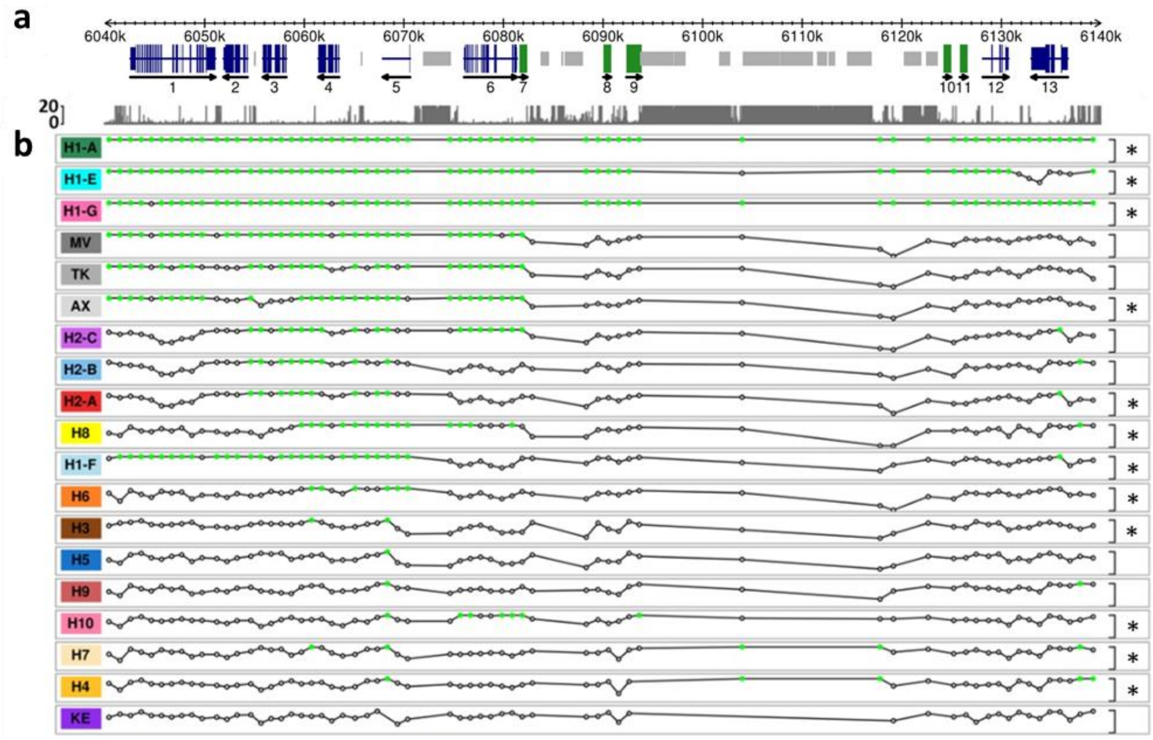

Supplementary Figure 38. Haplotypes identified in the selective sweep region on chromosome 17. a V2.1 gene models (exons in blue), manually curated gene predictions (green) in the isopiperitenol/carveol dehydrogenase gene cluster (gene IDs 7 →11), annotated transposable elements (light grey) and plot of 19–mer counts (dark grey). **b** Nucleotide identity with the reference haplotype (H1–A) in non–overlapping windows of 1 Kb of non–repetitive DNA. The interval depicted reports from 97 % (min) to 100 % (max, green dots) nucleotide identity. Asterisks indicate the haplotypes that were tested for allele–specific expression.

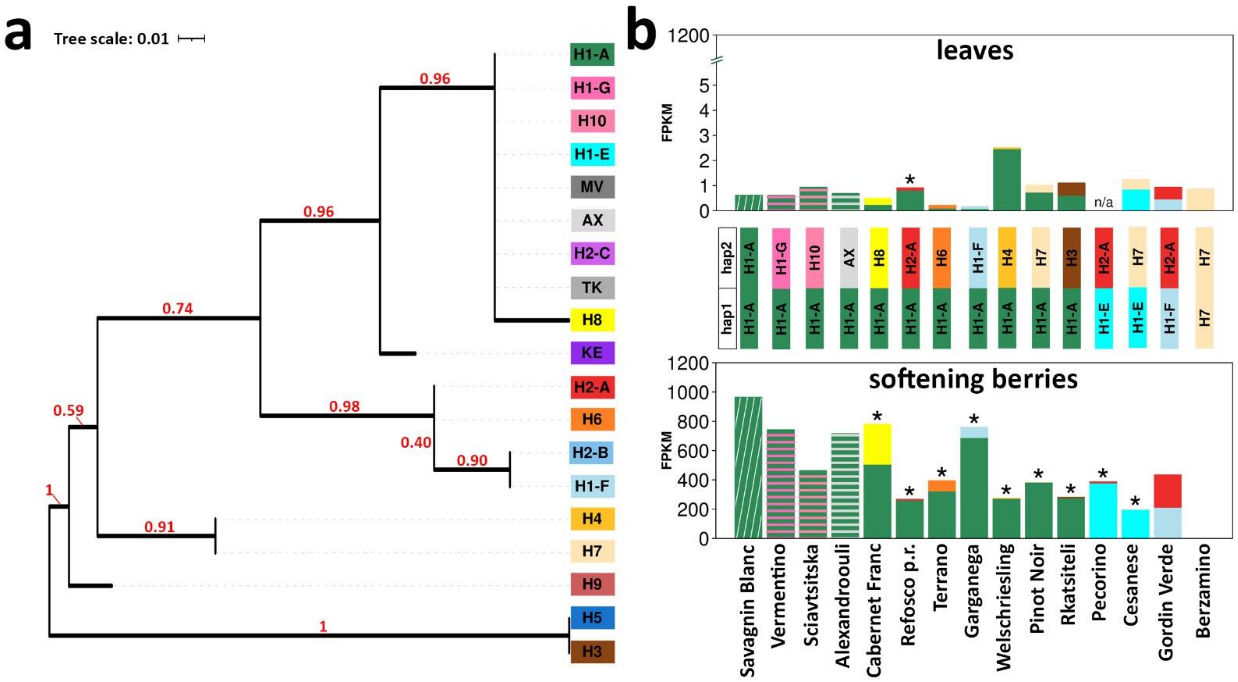

Supplementary Figure 39. Allele specific expression (ASE) of the isopiperitenol/carveol dehydrogenase *VIT_217s0000g05580*. a *VIT_217s0000g05580* gene phylogeny. Numbers indicate the proportion of bootstrap trees supporting that clade. **b** ASE of the isopiperitenol/carveol dehydrogenase *VIT_217s0000g05580* alleles in representative varieties of 15 haplotypic combinations, in softening berries (lower panel) and leaves (upper panel). The asterisks indicate statistically significant ASE levels (*p*–value <0.05) according to a Stouffer's meta–analysis with weight and direction effect using *n* = 2 biologically independent samples. Cumulative expression is reported for each haplotypic combination lacking exonic SNPs in *VIT_217s0000g05580* (H1–A/H1–G, H1–A/H10, H1–A/AX) and for a control variety homozygous for the H1–A haplotype. Gene expression for three haplotype combinations (H1–A/H10, H1–A/H6, H1–A/H4) was quantified in leaves of three different representative varieties (Tschvediansis Tetra, Picolit, Lambrusco Grasparossa) with the same genotype with respect to those used for berry gene expression. Source data of gene expression are provided as a Source Data file.

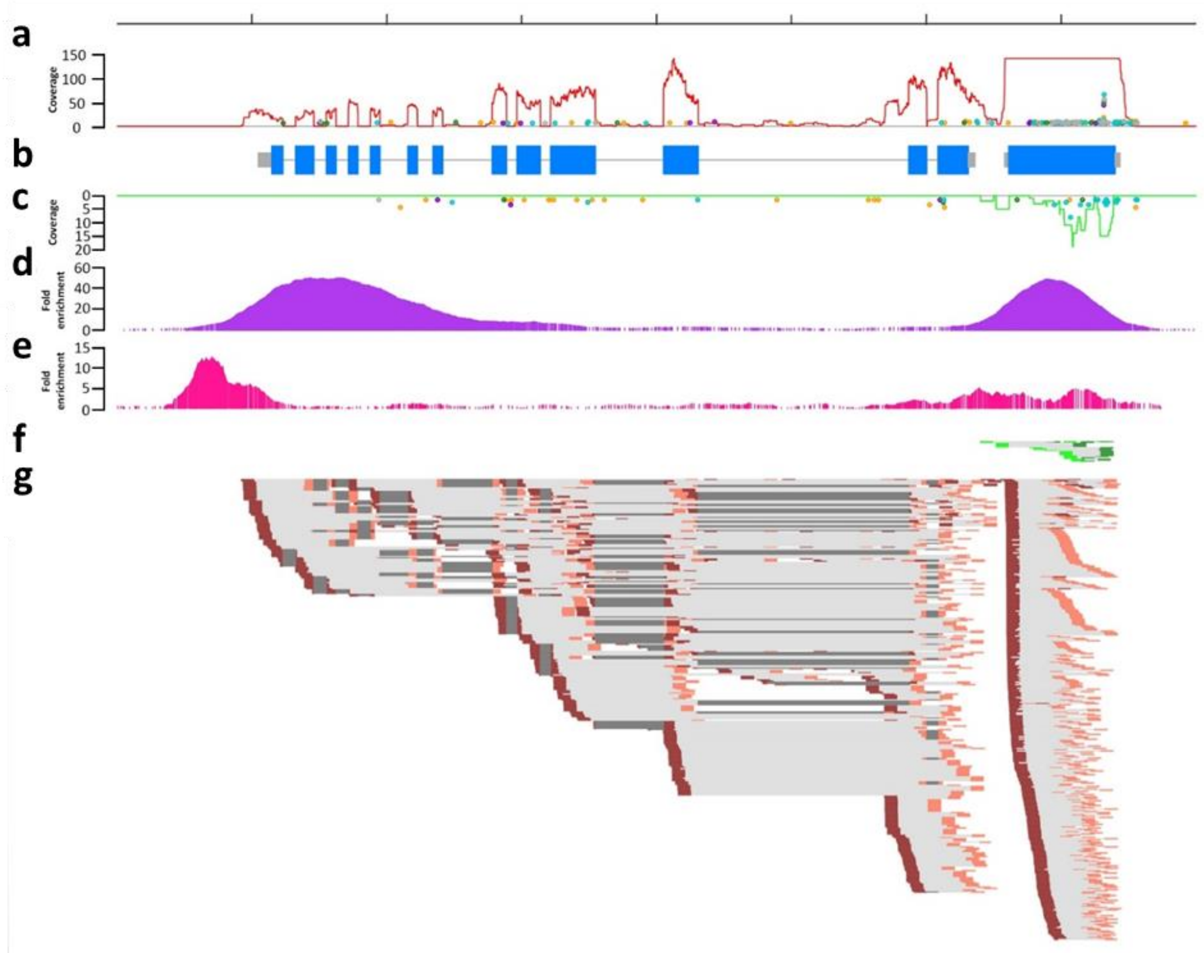

Supplementary Figure 40. Gene view of mRNA and small RNA coverage of the head–to–tail gene pair *VIT_217s0000g05570–VIT_217s0000g05580* in Cabernet Franc leaves and in Cabernet Sauvignon berries, respectively. Coverage of mRNA transcribed from the positive and the negative strand is reported, respectively, in (**a**) and (**c**), with respect to the intron–exon structure that is depicted in (**b**). Coverage values greater than 150 were levelled to 150. **b** Blue boxes indicate CDS, grey boxes indicate UTRs, grey lines indicate introns. Read alignments on the negative strand (green paired–end reads) and the positive strand (red paired–end reads) are reported in (**f**) and (**g**), respectively. For each RNA fragment, R1 reads are dark colored, R2 reads are light colored. Light grey connectors connect paired–end reads. Dark grey connectors connect split reads. Reads counts of small RNAs on the positive and the negative strand are reported, respectively, in (**a**) and (**c**) as colored dots. The color of the dot indicates reads size (20 bp, grey; 21 bp, cyan; 22 bp, green; 23 bp, purple; 24 bp, orange). Small RNA data were obtained from the website https://mpss.danforthcenter.org/[10] and correspond to GEO Accession Numbers GSM2279692–9739. **d** and **e** H3K4me3 and ATAC signals, respectively, that are derived from data deposited under the BioProject number PRJNA643441[11]. Ticks on the ruler indicate 1 Kb intervals in the region chr17:6,075,000..6,083,000.

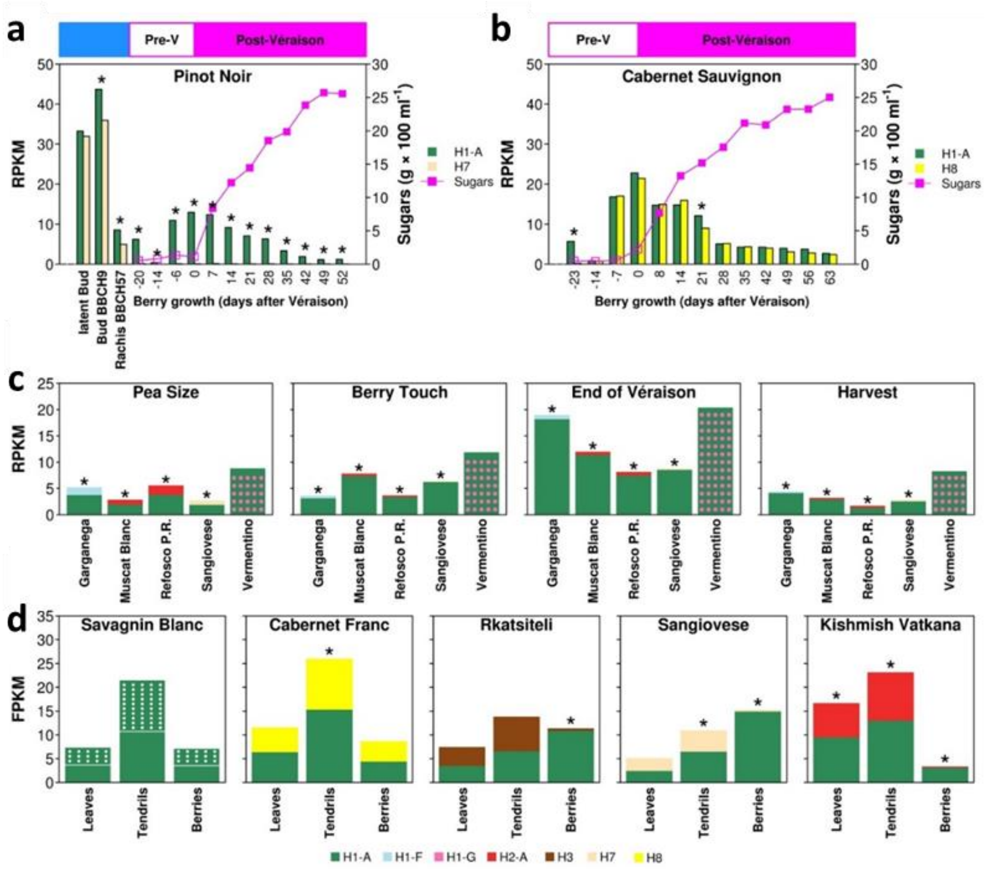

Supplementary Figure 41. Allele Specific Expression (ASE) of the LRR receptor kinase *VIT_217s0000g05570* during berry growth and ripening, and in other organs. ASE of *VIT_217s0000g05570* alleles in the common haplotype H1–A and: **a**–**b** in two alternative haplotypes (H7 and H8) in the heterozygous varieties Pinot Noir (**a**) and Cabernet Sauvignon (**b**), at three stages of inflorescence development within latent and bursting buds and just prior to anthesis in Pinot Noir[12] as well as the progression of berry growth and ripening in both Pinot Noir and Cabernet Sauvignon, as illustrated by the curve of sugars concentration (shown on the secondary *y*–axis) at Pre–Veraison (empty squares) and Post–Veraison (full squares) stages[13]; **c** in three alternative haplotypes (H1–F, H2–A and H7) in berries at four developmental stages in the heterozygous varieties Garganega, Muscat à Petits Grains Blancs, Refosco dal Peduncolo Rosso, Sangiovese and Vermentino[14] (the latter carrying the H1–G haplotype that lacks exonic SNPs in *VIT_217s0000g05570* for ASE quantification); **d** and in four alternative haplotypes (H2–A, H3, H7 and H8) in three organs (leaf, tendril and berry) of the heterozygous varieties Kishmish Vatkana, Rkatsiteli, Sangiovese and Cabernet Franc and in the control homozygous variety Savagnin Blanc (H1–A/ H1–A). The asterisks indicate statistically significant ASE levels (p–value <0.05) according to a Stouffer's meta–analysis with weight and direction effect using n = 3 biologically independent samples in **a**–**c** and n = 2 biologically independent samples in **d**. Plant material shown in **d** was collected following the same sampling scheme for field plot replication as described for berry sampling in the main text. Sampling of this plant material was performed on the same day for all organs (leaves, tendrils, and berries at *véraison*) and varieties. Raw reads are deposited under the BioProject number PRJNA373967. Source data are provided as a Source Data file.

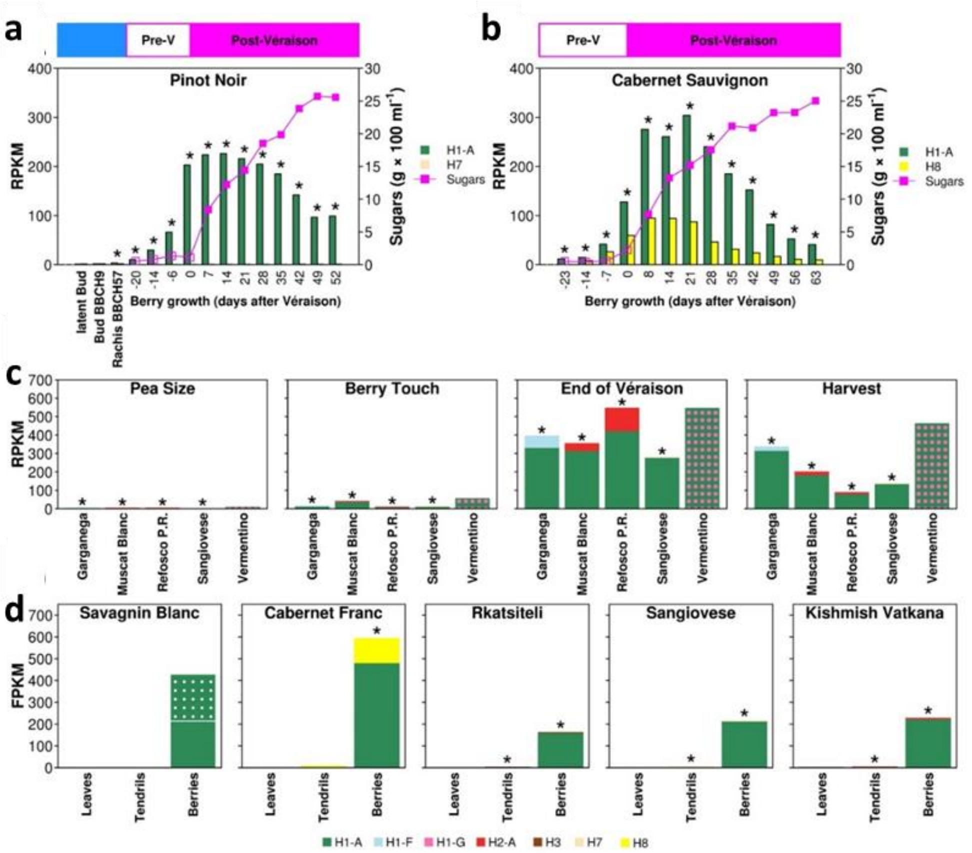

Supplementary Figure 42. Allele Specific Expression (ASE) of the isopiperitenol/carveol dehydrogenase *VIT_217s0000g05580* during berry growth and ripening, and in other organs. ASE of *VIT_217s0000g05580* alleles in the common haplotype H1–A and: **a–b** in two alternative haplotypes (H7 and H8) in the heterozygous varieties Pinot Noir (**a**) and Cabernet Sauvignon (**b**), at three stages of inflorescence development within latent and bursting buds and just prior to anthesis in Pinot Noir[12] as well as the progression of berry growth and ripening in both Pinot Noir and Cabernet Sauvignon, as illustrated by the curve of sugars concentration (shown on the secondary y–axis) at Pre–Veraison (empty squares) and Post–Veraison (full squares) stages[13]; **c** in three alternative haplotypes (H1–F, H2–A and H7) in berries at four developmental stages in the heterozygous varieties Garganega, Muscat à Petits Grains Blancs, Refosco dal Peduncolo Rosso, Sangiovese and Vermentino[14] (the latter carrying the H1–G haplotype that lacks exonic SNPs in *VIT_217s0000g05570* for ASE quantification); **d** and in four alternative haplotypes (H2–A, H3, H7 and H8) in three organs (leaf, tendril and berry) of the heterozygous varieties Kishmish Vatkana, Rkatsiteli, Sangiovese and Cabernet Franc and in the control homozygous variety Savagnin Blanc (H1–A/ H1–A). The asterisks indicate statistically significant ASE levels (p–value <0.05) according to a Stouffer's meta–analysis with weight and direction effect using $n = 3$ biologically independent samples in **a–c** and $n = 2$ biologically independent samples in **d**. Plant material shown in **d** was collected following the same sampling scheme for field plot replication as described for berry sampling in the main text. Sampling of this plant material was performed on the same day for all organs (leaves, tendrils, and berries at *véraison*) and varieties. Raw reads are deposited under the BioProject number PRJNA373967. Source data are provided as a Source Data file.

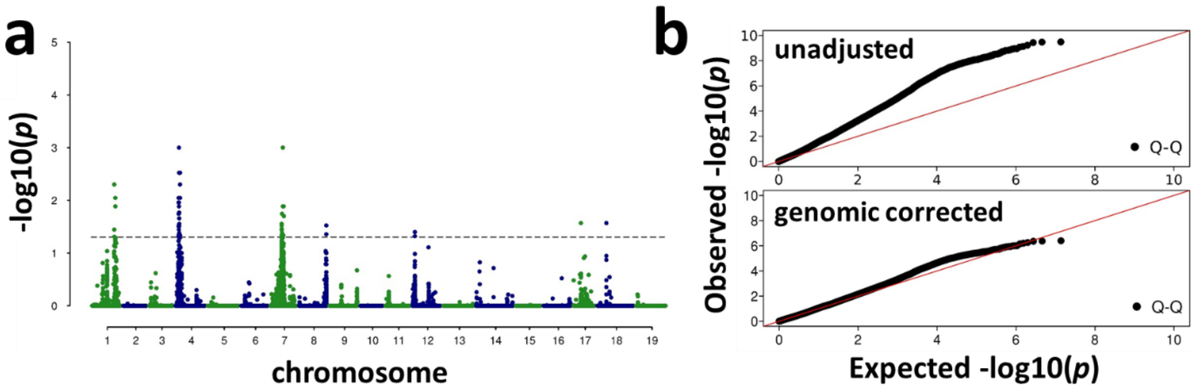

Supplementary Figure 43. Manhattan and quantile–quantile (Q–Q) plots of a genome–wide association study (GWAS) of seed–to–berry ratio at the onset of berry ripening using PLINK. A total of 88 accessions were used (Supplementary Data 3), excluding Sultanina and Kishmish Vatkana, which carry only remains of undeveloped seeds due to stenospermocarpy—a trait that is chiefly due to a missense mutation in the MADS−Box gene VviAGL11 at the *SEED DEVELOPMENT INHIBITOR* (*SDI*) locus on chromosome 18[15]. **a** Manhattan plot. The horizontal dashed line indicates the threshold for statistical significance that was set at $-\log_{10}(\alpha)$, with $\alpha = 0.05$. **b** Q–Q plots of unadjusted and genomic control corrected data.

Supplementary Table 1. Three–population test with WGS data.

Admixed population	Ancestral populations		f_3	standard error	Z–score
Alpine wine grapes	Western sylvestris	Table grapes	-0.00407463	9.46E-05	-43.0768
Alpine wine grapes	Western feral	Table grapes	-0.00361943	8.57E-05	-42.2506
Western feral	Balkans and Magna Graecia	Western sylvestris	-0.00219031	6.07E-05	-36.0691
Western feral	Western sylvestris	Table grapes	-0.00216117	6.35E-05	-34.0589
Alpine wine grapes	Balkans and Magna Graecia	Western sylvestris	-0.00307836	9.14E-05	-33.677
Western feral	Alpine wine grapes	Western sylvestris	-0.00170597	5.15E-05	-33.112
Alpine wine grapes	Balkans and Magna Graecia	Western feral	-0.00259401	8.33E-05	-31.1378
Western feral	Caucasian wine grapes	Western sylvestris	-0.00161129	5.81E-05	-27.7225
Western feral	Western sylvestris	Eastern feral	-0.00157558	5.70E-05	-27.6624
Eastern feral	Eastern sylvestris	Table grapes	-0.00219012	0.000119507	-18.3262
Eastern feral	Western sylvestris	Table grapes	-0.00200506	0.000120881	-16.5871
Western feral	Western sylvestris	Eastern sylvestris	-0.000892464	6.74E-05	-13.2495
Eastern feral	Western feral	Table grapes	-0.00141947	0.000115135	-12.3287
Eastern feral	Balkans and Magna Graecia	Eastern sylvestris	-0.000815345	0.000122314	-6.666
Alpine wine grapes	Caucasian wine grapes	Western feral	-7.48E-06	9.71E-05	-0.077

Reported are the f_3 statistics (only negative values), f_3 standard error and Z–scores for all combinations of four groups of cultivated varieties and four groups of wild grapes shown in Fig. 1a of the main text. Due to the computational burden of the three–population test, we pruned the complete SNP dataset for linkage disequilibrium prior to analysis. Pruning was performed in sliding windows of 50 SNPs with a 10SNP overlap and removing SNPs with r^2>0.2. The LD–pruned dataset consisted of 1,548,295 SNPs.

Supplementary Table 2. Representativeness by country of the accessions used for the analyses of geographic distribution of ancestry components and association with climate variables.

Country	Alpha-3 code	Number of 'prime' varieties, by country, in 2016*	Number of varieties by country analysed for distribution of ancestry components and association with climate variables
Armenia	ARM	2	3
Austria	AUT	35	6
Bulgaria	BGR	18	1
Switzerland	CHE	222	8
Cyprus	CYP	2	2
Czech Republic	CZE	14	1
Germany	DEU	103	5
Algeria	DZA	7	4
Spain	ESP	162	87
France	FRA	266	73
Georgia	GEO	21	56
Greece	GRC	54	35
Croatia	HRV	9	5
Hungary	HUN	157	6
Israel	ISR	16	9
Italy	ITA	393	148
Kazakhstan	KAZ	14	-
Lebanon	LBN	5	7
Luxembourg	LUX	11	-
Moldova	MDA	87	4
North Macedonia	MKD	19	2
Morocco	MAR	15	12
Portugal	PRT	253	30
Romania	ROU	102	14
Russia	RUS	55	9
Slovakia	SVK	8	-
Serbia	SRB	31	2
Slovenia	SVN	34	3
Tunisia	TUN	15	7
Turkey	TUR	35	7
Ukraine	UKR	13	7
Afghanistan	AFG	-	4
Azerbaijan	AZE	-	10
Bosnia and Herzegovina	BIH	-	3
Iran	IRN	-	6
State of Palestine	PSE	-	1
Syrian Arab Republic	SYR	-	7
Tajikistan	TJK	-	3
Turkmenistan	TKM	-	4
Uzbekistan	UZB	-	14
	**all**	**2178**	**605**

*according to Anderson and Nelgen[16] and definitions therein. See Source Data file for further details on the variety list by country and their geographic locations.

Supplementary Table 3. GLM of climatic variables explaining the geographic distribution of four ancestry components in the cultivated compartment and correlation between the seven bioclimatic variables incorporated in the GLM and other bioclimatic variables.

Variables	W2 ancestry		C1 ancestry		C2 ancestry		W1 ancestry	
	Sign of coefficient	P	Sign of coefficient	P	Sign of coefficient	P	Sign of coefficient	P
Ancestry	-	**1.19E-08**	+	**1.36E-17**	+	**4.94E-12**	-	0.899885
Annual Mean Temperature	+	0.366351	+	**2.39E-15**	-	**1.84E-26**	+	0.139557
Isothermality (BIO2/BIO7) (* 100)	-	**3.12E-09**	-	0.043596	+	**2.93E-19**	+	0.33021
Temperature Annual Range (BIO5-BIO6)	+	**1.22E-19**	+	0.065614	-	**2.13E-32**	-	0.022053
Mean Temperature of Wettest Quarter	+	0.017952	+	0.292498	-	0.057371	-	**3.23E-05**
Precipitation of Wettest Quarter	+	**7.14E-25**	-	**1.54E-07**	-	**1.03E-07**	-	**1.76E-04**
Seasonal Precipitation	-	**2.95E-10**	-	**4.34E-04**	+	**1.9E-06**	+	**6.07E-36**
Seasonal Potential Evapotranspiration	+	0.39712	-	**3.51E-15**	+	**3.34E-15**	+	0.937383
Variance explained	0.52		0.48		0.47		0.41	

GLM variable	Positive correlation	Negative correlation
Annual Mean Temperature	Min Temperature of Coldest Month	Precipitation of Driest Month
	Mean Temperature of Driest Quarter	Precipitation of Warmest Quarter
	Mean Temperature of Warmest Quarter	Maximum daylength
	Mean Temperature of Coldest Quarter	Average daylength
	Precipitation Seasonality (Coefficient of Variation)	Standard deviation daylength
	Mean T° coldest month	
	Minimum daylength	
	Annual Potential Evapotranspiration	
	Winkler index	
Isothermality (BIO2/BIO7) (* 100)	-	-
Temperature Annual Range (BIO5-BIO6)	Mean Diurnal Range (Mean of monthly (max temp - min temp))	
	Temperature Seasonality (standard deviation *100)	
Mean Temperature of Wettest Quarter	-	-
Precipitation of Wettest Quarter	Annual Precipitation	
	Precipitation of Wettest Month	
	Precipitation of Coldest Quarter	
Seasonal Precipitation	Mean Temperature of Driest Quarter	Precipitation Seasonality (Coefficient of Variation)
	Annual Precipitation	Minimum daylength
	Precipitation of Driest Month	Seasonal Climatic water deficit
	Precipitation of Driest Quarter	Climatic water deficit seasonal sum
	Precipitation of Warmest Quarter	
	Maximum daylength	
	Average daylength	
	Standard deviation daylength	
Seasonal Potential Evapotranspiration	Winkler index	
	Mean Temperature of Warmest Quarter	
	Annual Potential Evapotranspiration	

Significant values (*p*–value <0.05) are highlighted in bold.

Supplementary Table 4. Variance of ancestry components in 605 varieties explained by seven climatic variables at the most traditional site of cultivation or under simulations that systematically displaced their geographic locations.

Ancestry component	Traditional sites of cultivation	Latitudinal displacement from traditional sites of cultivation						Longitudinal displacement from traditional sites of cultivation					
		Northward shift			Southward shift			Eastward shift			Westward shift		
		Lat +20 Km Lon +0 Km	Lat +50 Km Lon +0 Km	Lat +100 Km Lon +0 Km	Lat -20 Km Lon +0 Km	Lat -50 Km Lon +0 Km	Lat -100 Km Lon +0 Km	Lat +0 Km Lon +20 Km	Lat +0 Km Lon +50 Km	Lat +0 Km Lon +100 Km	Lat +0 Km Lon -20 Km	Lat +0 Km Lon -50 Km	Lat +0 Km Lon -100 Km
W2	0.52	0.46	0.45	0.51	0.46	0.48	0.45	0.48	0.46	0.43	0.50	0.46	0.42
C1	0.48	0.41	0.37	0.33	0.42	0.43	0.36	0.41	0.40	0.35	0.45	0.41	0.39
C2	0.47	0.28	0.24	0.29	0.31	0.36	0.42	0.35	0.35	0.35	0.33	0.32	0.32
W1	0.41	0.36	0.33	0.29	0.36	0.33	0.27	0.34	0.29	0.24	0.39	0.35	0.36
*	0	58	93	126	87	145	192	64	110	138	78	143	219

* number of cases in which the shift in geographic coordinates would have displaced the site offshore. The original site has been maintained in those cases.

Supplementary Note 1. Source of archived sequence reads

Of the 123 cultivated varieties analyzed in this study, 110 were newly sequenced. Among the rest, five were sequenced by Zhou and coworkers[17], one by Mercenaro and coworkers[18], three by Cardone and coworkers[19], one each by Carbonell–Bejerano and coworkers[20], Da Silva and coworkers[21], Di Genova and coworkers[22] and Liang and coworkers[23]. Of the 8 *Vitis* species analyzed in this study, two were newly sequenced. The rest was sequenced by Liang and coworkers[23], Ma and coworkers[24], and Girollet and coworkers[25]. Raw reads of two accessions of *Muscadinia rotundifolia* were obtained from Zhou and coworkers[17] and Liang and coworkers[23].

Supplementary Note 2. Classification of feral and wild *V. vinifera* accessions

We used 81 accessions that do not belong to the cultivated compartment, for which an *a priori* assignment either to the group of feral grapes and or to the wild compartment (*sylvestris*) is questionable if solely based on metadata or on the observation of phenotypic traits. We thus classified these accessions based on a model–based clustering approach resulting into the assignment reported in Fig. 1a of the main text. Of these, 48 appeared to be *bona fide* wild grapes according to *a posteriori* assignment: one accession of presumed eastern origin (V395 from this study), two accessions from the Caucasus (sample names armenia and georgia from Zhou and coworkers[17], hereafter eastern *sylvestris*), and 45 accessions from Germany[23] (hereafter western *sylvestris*, with > 0.99 *sylvestris* var. *typica* ancestry). The remaining 33 accessions were grapevines with uncertain traits of Central Asian and Caucasian (13) or European (20) origin, hereafter conservatively referred to as eastern feral and western feral, respectively, all showing < 0.99 *sylvestris* var. *typica* ancestry). Among them, 11 were sequenced in this study, including three accessions from the Dalmatian coast of Croatia[26], and the others were previously classified as *sylvestris* by Zhou and coworkers[17] and Liang and coworkers[23].

Supplementary Note 3. Analysis of Mendelian inconsistencies

Part of the homozygous Mendelian inconsistencies were not due to genotyping errors but they were generated in presence of hemizygous DNA. We estimated from genuine parent–offspring relationships that deletions in the transmitted parental chromosome have caused genomic windows of IBD=0 to appear in the offspring across a 6.3 % on average of the genome length (range of variation 1.5–11.6), with an even distribution across each genome (Supplementary Fig. 2). Part of the heterozygous Mendelian inconsistencies were instead due to somatic mutations that occurred during the clonal propagation of the variety. We called one non–parental SNP every 265.2 Kb in an offspring of a validated trio (Cabernet Sauvignon = Cabernet Franc × Sauvignon Blanc, Supplementary Fig. 3a). The distribution of read count ratios between derived and ancestral alleles of non–parental SNPs was right–skewed (mode = 0.2625 ± 0.0125) to values lower than 0.5 compared to germline SNPs with a mode = 0.495 ± 0.005 (Supplementary Fig. 3b), a situation that shows up with post–zygotic mutations in leaf DNA extracts from periclinal chimeras[27]. The reduced coverage of derived alleles in non–parental SNPs is

compatible with the assumption that the mutations occurred in the shoot apical meristem and remain confined into the cell layer in which they originated and they are diluted in the DNA extracted from chimerical leaves.

Supplementary Note 4. Mutation direction, site frequency spectrum, the strength and direction of selective pressure in cultivated varieties by mutation age and mutation type

For a subset of polymorphic sites in *sativa* that were informative in *M. rotundifolia* we determined the mutation direction and sorted the mutations by evolutionary age and by type. We identified 1,674,287 transitions and 1,067,466 transversions, with *ti/tv* 1.28 in exons, 1.27 in introns and 1.59 in the intergenic space. A:T→T:A changes were the more frequent transversions. C:G→G:C changes were the less frequent transversions (Supplementary Fig. 1l).

Out of all polymorphic sites that were informative in *M. rotundifolia*, 996,451 SNPs are recent and lineage–specific in *sativa* ($_{\text{sat}}$SNPs, Supplementary Fig. 4). They are highly skewed towards low derived allele frequencies (DAF, Supplementary Fig. 1b). Only 0.002 % of $_{\text{sat}}$SNPs are fixed. Another 1,128,867 (19.1 %) SNPs are species–specific in *V. vinifera* and predated domestication ($_{\text{Vv}}$SNPs). $_{\text{Vv}}$SNPs showed a less skewed DAF spectrum (Supplementary Fig. 1b), 1.9 % of them are fixed in *sativa* whereas 96.3 % are still polymorphic in both *sativa* and *sylvestris*. We also found 328,638 (5.5 %) more ancient SNPs that occurred before the split between European and Asian grapes, 18.9 % of which are fixed in *sativa*, whereas 73.3 % trans–specific remained polymorphic in both lineages (ts$_{\text{Vv/As}}$SNPs). Another 543,438 (9.2 %) SNPs are more ancient because they predated the split between American and Eurasian grapes, 31.7 % of which are fixed in *sativa*, 6.1 %, 15.8 %, 12.8 % (ts$_{\text{Vv/As}}$SNPs, ts$_{\text{Vv/Am}}$SNPs, and ts$_{\text{As/Am}}$SNPs, respectively) are trans–specific in Eurasian or in intercontinental comparisons (Supplementary Fig. 4).

Trans–specific SNPs and *vinifera* SNPs that predate domestication in the *vinifera* lineage showed largely positive values of Tajima's D in all genomic contexts of *sativa* (Supplementary Fig. 1c). Recent mutations in *sativa* ($_{\text{sat}}$SNPs) showed negative Tajima's D in all genomic contexts. Stop gain, splicing site, and deleterious non–synonymous mutations showed only slightly lower values compared to intergenic, intronic, synonymous, and tolerated non–synonymous mutations.

Nonsynonymous mutations were identified with ANNOVAR[28] and their functional effects were predicted with The Protein Variation Effect Analyzer software (PROVEAN version 1.1.5[29]). We used the default threshold of significant score < –2.5 for calling deleterious mutations. Cultivated varieties have a genetic load of recent deleterious—and most likely recessive—mutations (Supplementary Fig. 1d–e) that are largely maintained in the heterozygous state in single individuals within the population (Supplementary Fig. 1e). Mutations that occurred after domestication are present at highest frequencies in table grapes—the most ancient group of domesticated grapes—and at lowest frequencies in Alpine wine grapes—the most recently formed group *via* gene flow from *sylvestris*. About a half of the deleterious mutations that originated in the *vinifera* lineage before domestication (Supplementary Fig. 1f–g) became fixed in the isolated populations of *sylvestris* while only one third or one fourth of them became fixed in different groups of cultivated varieties. More ancient deleterious tsSNPs were driven towards

fixation at similar levels in *sylvestris* and *vinifera*. The set of most ancient tsSNPs showed more variation in their number per individual within groups—especially in Mediterranean grapes wine grapes—than among groups of cultivated grapes, possibly suggesting that ancient balanced polymorphisms may have provided the reservoir of genetic variation for recent differentiation.

Supplementary Note 5. Split and admixture events under the hypothesis of population structure shown in the main text

The unrooted maximum likelihood tree before adding any migration event explained 94.01 % of the variance of relatedness between populations and showed residuals above zero that strongly indicate admixture between *occidentalis*, on the one side, and western *sylvestris*, western feral grapes, *balcanica* and *orientalis*, on the other side (Supplementary Fig. 13a). Moderately positive residuals also suggest as admixture among *orientalis*, *georgica* and eastern *sylvestris* as well as between *orientalis* and eastern feral grapes.

With one migration event (Supplementary Fig. 13b), the maximum likelihood tree showed migration from a progenitor population of modern western *sylvestris* accessions into *occidentalis* (variance of relatedness between populations explained by this model = 98.73 %). Residual fit indicate that the origin of *occidentalis* is best explained by this model, without further admixture involving *occidentalis*. Residuals above zero still indicate further admixture in the West between *balcanica* and western feral grapes (Supplementary Fig. 13b).

Supplementary Note 6. Split and admixture events under four alternative hypotheses of population structure

In order to test the impact of the K choice in population structure analysis on the grouping of cultivated varieties later used for inferring splits and admixture between ancestral populations, we run TreeMix analysis and generated ABBA–BABA statistics for western *sylvestris* introgression under the following alternative hypotheses:

Three–population scenario with (1) pontica georgica, (2) orientalis, (3) occidentalis ancestral populations and pontica balcanica discarded, according to Negrul's taxonomy treatment

The unrooted ML tree before adding any migration event showed residuals above zero that strongly indicate admixture between *occidentalis*, one the one side, and western *sylvestris*, western feral grapes and *orientalis* table grapes, on the other side (Supplementary Fig. 15a). With one migration event, the ML tree showed migration from a progenitor population of western *sylvestris* into *occidentalis* (Supplementary Fig. 15b). The residuals also suggest admixture with *orientalis* table grapes to explain the origin of *occidentalis*.

Three–population scenario with (1) pontica georgica, (2) orientalis, and (3) an extended group of occidentalis. The occidentalis group encompasses in this simulation most of the European diversity (i.e. including pontica balcanica)

The unrooted ML tree before adding any migration event showed residuals above zero that strongly indicate admixture between European wine grapes, one the one side, and western *sylvestris*, western feral grapes and *orientalis* table grapes, on the other side (Supplementary Fig. 16a). The ML tree with one migration event suggested migration from eastern *sylvestris* into the domesticated lineage generating eastern feral grapes. The second migration event from a progenitor population of western *sylvestris* into the domesticated lineage explained the origin of European wine grapes (Supplementary Fig. 16b).

Considering one single eastern ancestral population (including both pontica georgica and orientalis) of cultivated varieties and simulating a three–population scenario with (1) eastern diversity, (2) balcanica and (3) occidentalis, according to the K = 3 ADMIXTURE grouping

The unrooted ML tree before adding any migration event showed residuals above zero that strongly indicate admixture between *occidentalis*, one the one side, and western *sylvestris*, western feral, *balcanica* and to a lesser extent the whole eastern cultivated germplasm, on the other side (Supplementary Fig. 17a). The ML tree with one migration event suggested migration from progenitors of western *sylvestris* into the lineage of eastern *sylvestris*. With the second migration event, the ML tree showed admixture between progenitors of western *sylvestris* and the domesticate lineages, generating *occidentalis* (Supplementary Fig. 17b)

Considering one single eastern ancestral population (including both pontica georgica and orientalis) of cultivated varieties and simulating a scenario with (1) eastern and (2) occidentalis ancestral populations, according to the K = 2 ADMIXTURE grouping

The unrooted maximum likelihood tree before adding any migration event showed residuals above zero that strongly indicate admixture between *occidentalis*, one the one side, and western *sylvestris*, western feral grapes and eastern cultivated germplasm, on the other side (Supplementary Fig. 18a). The ML tree with one migration event suggested ancient admixture between populations in *sylvestris*. The second migration event from a progenitor population of western *sylvestris* into the domesticated lineage explained the origin of *occidentalis* (Supplementary Fig. 18b).

Supplementary Note 7. Revised first–degree relationships using WGS data

The assessment of the degree of consanguinity based on the aggregate length and the distribution of IBD=0, IBD=1 and IBD=2 windows across the genome allowed us to resolved first–degree relationships with an unprecedented level of accuracy. We provide here the examples of two opposite cases of one genuine parent–offspring relationship (Heunisch Weiss and Ribolla Gialla)

that was previously mistaken for a full–sibling relationships and one genuine full–sibling relationship (Schiava Gentile and Schiava Grossa) that was previously mistaken for a parent–offspring relationship.

Heunisch Weiss and Ribolla Gialla

Heunisch Weiss and Ribolla Gialla showed an aggregate length of IBD=0 segments amounting to 32.6 Mb (7.7 % of the genome length, Supplementary Fig. 25), which is within the range of the IBD=0 experimental error observed in known parent–offspring pairs (Supplementary Fig. 2). Tracts with IBD=0 between Heunisch Weiss and Ribolla Gialla were small and evenly distributed across the chromosomes. In several cases, they were associated with single genomics windows in pericentromeric regions, where tracts of hemizygous DNA are more likely to occur and to cause fictitious variants sites with IBS=0 (Supplementary Fig. 2). We did not find a single tract of extended IBD=0, which is otherwise expected to occur in full–sibling relationships. Nor we found tracts of extended IBD=2, which also occur in full–sibling relationships. The aggregate length of IDB=2 segments amounted to 16.3 % of the genome length. The large majority of genomic windows (76 %) was in an IBD=1 condition.

In grapevine literature reports, Heunisch Weiss and Ribolla Gialla were profiled at 58 short tandem repeats (STRs) and assigned a full–sibling relationship, because they did not display matching alleles[5] at six of these STRs. These Mendelian inconsistencies led the authors to exclude the possibility of a parent–offspring relationship. We extracted nucleotide variant sites flanking the positions of the non–matching STRs reported by[5] and plotted the IBS condition between Heunisch Weiss and Ribolla Gialla across these sites. In all six cases, the non–matching STR alleles are located within DNA tracts with at least one shared haplotype based on SNP profiles (Supplementary Fig. 26). The non–matching STR alleles originated at hypervariable sites within otherwise identical haplotypes.

Our conclusions contrast with[5] and agree with[30], providing stronger support to the recently proposed hypothesis of a parent–offspring relationships between Heunisch Weiss and Ribolla Gialla. While the discrimination between the two options of first–degree relationship was based on cumulative probabilities of IBD allele sharing per locus in the analysis of Crespan and coworkers[30], we provided here more robust evidence based on haplotype sharing.

Schiava Gentile and Schiava Grossa

Schiava Gentile and Schiava Grossa showed an aggregate length of IBD=0 segments amounting to 54.7 Mb (13 % of the genome length), which fall short of the range of the IBD=0 experimental error observed in known parent–offspring pairs (Supplementary Fig. 2). At least four extended tracts with IBD=0 between Schiava Gentile and Schiava Grossa on chromosomes 3, 10, 14 and 17 are incompatible with the Mendelian inheritance expected in a parent–offspring relationship (Supplementary Fig. 27). The aggregate length of IBD=2 segments amounted to 40 % of the genome length. The aggregate length of IBD=1 segments amounted to 47 % of the genome

length. Extended chromosome tracts in an IBD=2 condition were interspersed with extended chromosome tracts in an IBD=1 condition.

While excluding beyond doubt a parent–offspring relationship between Schiava Gentile and Schiava Grossa, the data on individual and aggregate length of shared haplotypes is most likely compatible with the condition that shows up in the comparison between full–siblings.

In previous literature reports, Schiava Gentile and Schiava Grossa showed one or two matching alleles at all 20 STRs tested by Lacombe and coworkers[6], which led those authors to assign them a parent–offspring relationship. Supplementary Fig. 27 shows that 18 out of the 20 STRs tested by Lacombe and coworkers[6] are, indeed, located in 100–Kb windows of non–repetitive DNA with IBD=1 or IBD=2. Only two STRs VVMD28 (chr3) and VMC4F3–1 (chr12) are located in 100–Kb windows of non–repetitive DNA with IBD=0. We extracted SNPs around the STR and plotted IBS between Schiava Gentile and Schiava Grossa (Supplementary Figs. 28–29). SNPs flanking the STR revealed that both VVMD28 and VMC4F3–1 are located within chromosome segments that are not identical by descent (Supplementary Fig. 28). However, the VVMD28 locus is located within a smaller interval with either reduced diversity or in an IBD=1 condition between Schiava Gentile and Schiava Grossa (Supplementary Fig. 29). The VMC4F3–1 locus is actually located in region in an IBD=0 condition between Schiava Gentile and Schiava Grossa but the authors unfortunately detected size homoplasy in amplicons containing the STR. Lacombe and coworkers[6] used all care in the design of a marker panel that aimed at analyzing at least one STR per chromosome. Unfortunately, the combination of random sampling and homoplasy did not allow the authors to spot any of the large block of IBD=0 between Schiava Gentile and Schiava Grossa that are present on chromosomes 3, 10, 14 and 17, which would have disclosed Mendelian inconsistencies incompatible with a parent–offspring relationship.

Supplementary Note 8. Principal component analysis (PCA) and principal coordinate analysis (PCoA)

The first component in PCA with individual SNPs (Fig. 3 of the main text) and in PCoA with matrices of genotypic and haplotypic distances (Supplementary Fig. 31a–b) reflected an east–to–west gradient of genetic diversity from positive to negative PC and PCoA values in the wild and in cultivated compartments. However, the diversity in the cultivated germplasm along the first component spanned only approximately half of diversity in the wild and corresponded only to that part of *sylvestris* diversity of eastern origin in PCA and PCoA based on the matrix of genotypic distance. This span was estimated to roughly one third of the *sylvestris* diversity using a PCoA based on a matrix of haplotypic distance.

The second component in PCA and PCoA reflected a gradient of domestication.

Table grapes overlapped with feral forms of eastern origin on the PCA and PCoA bi–dimensional spaces, suggesting that this group of cultivated grapes is closest to wild or para–domesticated germplasm from the same geographical area. It is possible that some feral forms from the Caspian Sea Basin, such as those analysed in this paper, may include relicts of *sylvestris*

var. *aberrans* populations because one representative of them (V297) carries a unique and highly divergent *vinifera* chlorotype not found so far in *sylvestris* var. *typica*[31]. Caucasian wine grapes are located in the PCA and PCoA planes midway between table grapes and eastern *sylvestris*. PCA of the diversity panel (Fig. 3) showed that part of the Caucasian wine grapes—especially primitive varieties in western Georgia—is contiguous to Georgian *sylvestris*. According to a model–based clustering approach (Fig. 1), we found the highest eastern wild ancestry proportion in Adjaruli Tetri, Mgaloblishvili and Ojaleshi that are cultivated in temperate and humid western Georgia as well as in the feral grape accessions V267, V411, V278, V389. Traditional wine grapes from eastern Georgia (i.e. Rkatsiteli) and dual–use grapes from Azerbaijan and Dagestan (i.e. Bayan Shirei and Asyl Kara), collectively representing germplasm from arid regions of the Caspian Sea shores, are more shifted from Georgian *sylvestris* towards table grapes. Western wine grapes showed increasingly more negative PC1 values, similar to those observed in *sylvestris* populations from eastern Europe, Southern Balkans, Northern Africa and the Italian peninsula but not as negative as those observed in *sylvestris* populations from Western Europe.

PCA was also performed in the WGS panel using small Indels, which were identified with GATK using gapped read alignments. Using Indels, we explained a slightly lower percentage of the variance with the first two PCA components (Supplementary Fig. 31c), compared to the PCA with SNPs (Fig. 3a), but we obtained an identical distribution of the accessions in the bidimensional space.

Supplementary Method 1. *In silico* SNP validation

Estimation of genotype error rate in the presence of a reference haplotype

The strain PN40024, which was used for the assembly of the reference genome[32], was derived from selfing of Helfensteiner, which is an offspring of Pinot Noir and Schiava Grossa. We expected large blocks of the reference sequence to be identical to one haplotype present in Pinot Noir in heterozygous or homozygous states. We also expected IDB=0 between PN40024 and Pinot Noir for a substantial fraction of the genome, corresponding to the part of the haploid genome that Schiava Grossa has donated to Helfensteiner, not shared with Pinot Noir and retained in PN40024. Based on values of $IBSR_H$ (Equation **1**) and genD (Equation **2**) we identified windows of haplotype sharing (IBD=1 or IBD=2) across the genome. The diploid genome of Pinot Noir has the reference haplotype (PN40024) along 53.2 % of the haploid genome length. Along 4.8 % of the haploid genome length, Pinot Noir is homozygous for the reference haplotype of PN40024. Along 46.2 % of the haploid genome length, Pinot Noir does not have the reference haplotype. For the remainder 0.6 % of the haploid genome length, we could not determine the level of haplotype sharing due to low coverage.

We used more stringent criteria for selecting regions of haplotype sharing to be used for SNP validation. Only stretches of ≥ 3 consecutive windows with IBD=1 or IBD=2 were considered. In such stretches, the initial and the final window were not used for computation. A total of 175.1 Mb, containing 82.5 Mb mappable sites, were finally available for the validation in regions of with IBD=1. We called a total of 539,361 variant sites between Pinot Noir and PN40024 in regions with IBD=1. In Pinot Noir, 539,274 genotype calls were heterozygous (99.98%) and 87 (0.02%) were homozygous for alternative alleles. This rate corresponded to one false homozygous SNP call every 0.9 Mb. A total of 5.3 Mb in the reference sequence, containing 2.3 Mb mappable sites, was finally available for the validation in regions of with IBD=2. We called 214 variant sites between Pinot Noir and PN40024 in regions with IBD=2. In Pinot Noir, 211 genotype calls were heterozygous (98.7 %) and 3 genotype calls were homozygous alternative. This rate corresponded to one false heterozygous genotype call every 9.81 Kb and one false homozygous genotype call every 752.7 Kb.

Estimation of genotype error rate between individuals

Pinot Noir and Savagnin Blanc have a parent–offspring relationship. Across the genome, they share either one or two haplotypes. Windows with IBD=1 amounted to 286.4 Mb and contained 120.6 M mappable sites. In these regions, a total of 1,216,604 SNPs were called in Pinot Noir and/or Savagnin Blanc with respect to the reference genome. Genotype calls in Pinot Noir and Savagnin Blanc were compatible with sharing one allele in 99.4 % of variant sites. Pinot Noir and Savagnin Blanc were called homozygous for alternative alleles at one site every 15.8 Kb. Windows with IBD=2 amounted to 116.6 Mb. The longest segments of adjacent windows with IDB=2 are located on chromosome 4 and chromosome 15, amounting to 22.7 Mb and 17.2 Mb, respectively. On chromosome 4, 76,422 SNPs were called against the reference genome. Genotype calls in Pinot Noir and Savagnin Blanc were concordant for 99 % variant sites. Out of 9.5 M mappable sites, we called one wrong genotype in either Pinot Noir or Savagnin Blanc

every 12.1 Kb. Among the discordant variant sites, 611 genotypic calls were AA:AB, where A stands for the reference allele, 169 genotypic calls were BB:AB, and 3 genotypic calls were AA:BB. On chromosome 15, 37,350 SNPs were called against the reference genome. The genotype of Pinot Noir and Savagnin Blanc was concordant for 98.4 % variant sites. Out of 5.5 M mappable sites, we called one wrong genotype in either Pinot Noir or Savagnin Blanc every 9.4 Kb. Among the discordant variant sites, 519 genotypic calls were AA:AB, where A stands for the reference allele, and 65 genotypic calls were BB:AB. We did not call AA:BB genotypes at any site. Overall, genotype error rate between Pinot Noir and Savagnin Blanc was estimated at one false genotype call every 15.1 kb. We also identified 86 windows with IBD=0 amounting to 17.5 Mb. We hypothesize that these windows contain hemizygous DNA. In the windows in which the parent–offspring pair shares by descent the haplotype carrying the deletion, we miscall A0┃B0 genotypes as false AA┃BB genotypes, mistaking IBD=1 for IDB=0.

Supplementary Method 2. Experimental SNP validation

We experimentally validated a set of predicted SNPs in Sangiovese. Sequencing data were obtained via targeted resequencing of 736 regions enriched using the Single Primer Enrichment Technology, SPET (NuGEN, San Carlos, CA, USA). We generated 250–bp paired–end SPET reads with an Illumina Hiseq sequencer. Raw reads are archived under the BioProject number PRJNA373967. Genotypes were called from SPET reads using GATK. After masking for SPET probes, repetitive DNA and the final 10 bp of the target region, we retained 66,456 sites that concomitantly had ≥ 100X coverage of SPET reads and informative genotype calls from whole genome sequencing (WGS). Genotype calls in 543 variant sites from Sangiovese WGS data were validated by SPET resequencing with a rate of 98.2 %. As for false negatives, 72 variant sites were called from Sangiovese SPET resequencing data and were missing from the final list of Sangiovese WGS variant sites. Of these, 35 variant sites were called from Sangiovese WGS data, but they were filtered because of triallelic patterns in the population or because the site was informative in < 50 % of the analyzed accessions. Six more sites were not called in Sangiovese from WGS data, they were called in other accessions from WGS data, but they were filtered for the same reasons as above. Twenty–one sites were called in Sangiovese from SPET resequencing data, but they were not called in any accession from WGS data. These sites are preferentially located at the borders of the regions captured by SPET, more likely representing false positives in SPET resequencing calls rather than false negatives in WGS calls. Ten sites were not called in Sangiovese from WGS data, they were called in other accessions from WGS data, passing all filters and providing a reliable estimation of our false negative calls from a single accession.

Supplementary Method 3. Library preparation and WGS sequencing

Genomic DNA was extracted from subapical leaves and sheared by sonication. Paired–end libraries were generated from genomic DNA, according to the standard Illumina paired–end sample preparation guide (Illumina Inc., San Diego, CA, USA) with slight modifications. Sonicated DNA was treated with T4 DNA polymerase and Klenow enzyme. The 3′ ends of DNA

fragment were A–tailed and ligated to Illumina adaptors. The libraries were immobilized onto Illumina flow–cells via the Illumina Cluster Generation Station (cBot) and sequenced by synthesis using Illumina Genome Analyzer II, HiSeq2000, and HiSeq2500 equipment. Sequencing cycles ranges from 101 to 133. The CASAVA 1.8.2 version of the Illumina pipeline was used to process raw reads.

Supplementary Method 4. Trimming, filtering, alignment

Raw sequences were trimmed for quality. Contaminant reads, including organelle sequences, were filtered using erne–filter version 1.2 and adapters were removed with cutadapt version 1.1[33]. Short reads sequences were then mapped using the software package BWA version 0.7.5a[34] with the default settings (seed length 32, mismatch penalty 3, gap open penalty 11, gap extension penalty 4). The GATK RealignerTargetCreator command was used to identify intervals spanning indels for local realignment. Local realignment was performed using IndelRealigner with default settings. The output of the aligner in Sequence Alignment/Map (SAM) format was sorted and transformed to Binary Alignment/Map (BAM) file through the software package SAMtools version 0.1.18[35]. PCR duplicates were removed with samtools rmdup command and uniquely aligned reads were selected for further analyses. The mean coverage of each individual was calculated by dividing the total number of uniquely aligned bases by the number of covered positions. The physical coverage was computed as above, but considering the insert size information, including thus the bases not sequenced, but comprised between the two sequenced reads.

Supplementary Method 5. ddRAD sequencing of segregating progeny and genetic maps construction

Two S1 families, consisting of 85 and 79 offspring each, were generated by self–pollination of the highly heterozygous cultivated varieties Schiava Grossa and Pinot Noir, respectively. Schiava Grossa and Pinot Noir are the grandparents of PN40024—the line previously used for assembling the grapevine reference genome. Leaves or cotyledons were harvested from seedlings within two months after germination for DNA extraction. Parents and seedlings were genotyped by double digest Restriction Site Associated DNA Sequencing (ddRAD–seq). Libraries were generated using the procedure described by[36], using the restriction enzymes SphI–HF and MboI restriction enzymes (New England Biolabs, Ipswich, MA). Digested DNA was purified wuth Agencourt AMPure XP beads (Beckman Coulter, Brea, CA) and adapter–ligated fragments in the size range between 300 and 450 bp were recovered from 1.5% agarose gel after electrophoresis separation out using MinElute Gel Extraction Kit (Qiagen, Hilden, DE). Libraries were sequenced on an Illumina HiSeq2500. Raw reads were deposited under the BioProject number PRJNA373967. Trimming, aligning and SNP calling were carried out using the Stacks software package[37]. Reads were aligned with the scaffold sequences of the *Vitis vinifera* 12Xv0 genome assembly (GCA_000003745.2) and ddRAD markers were named after the scaffold number and the variant position in the scaffold. Genetic maps were generated with Lep-MAP2[38]

using markers that were informative in > 50% of the progeny. Linkage groups were generated with a LOD of 15. Marker order was generated after four rounds of ordering.

Variant sites in non–repetitive DNA regions (12,690 and 7,457 in Pinot Noir and Schiava Grossa, respectively) were used during the first round of grouping, which included running the JoinSingles module for adding singular markers to the established linkage groups, and ordering. The output of the first round was used for (1) removing unassigned markers as well as markers assigned to LGs but associated with a genotype error rate estimate >0.1, (2) orienting scaffolds by comparing relative (within–scaffold) coordinates and cM of the markers, and (3) correcting residual genotyping errors with the SMOOTH software[39]. Variant sites in repetitive DNA regions (6,895 and 4,079 in Pinot Noir and Schiava Grossa, respectively) were added to the filtered genotypic matrices resulting from the first round. Grouping, ordering, filtering and error correction in the second round were conducted with the same parameters as in the first round, except for the removal of markers with genotype error rate estimates that were more stringent (>0.09) for variant sites residing in non–repetitive DNA regions and highly stringent (>0.01) for variant sites residing in repetitive DNA regions. Grouping and ordering in the third round were conducted with the same parameters as in previous rounds, resulting into 22 LGs in each map and containing 16,358 markers with a total length of 1,383.28 cM in Pinot Noir as well as 10,179 markers with a total length of 1,139.51 cM in Schiava Grossa. A final fourth round of grouping and ordering was run after the removal of markers from the ends of LGs (if missing data were >10 %) and using a LOD of 10.

Supplementary Method 6. Genome segmentation and IBD detection in extended haplotypes

Pairwise IBD was estimated in each genome window of 100 Kb non–repetitive DNA, based on thresholds of identity–by–state ratio ($IBSR_H$, see equation 1) and genotypic distance (genD, see equation 2). We introduced a modification in the computation of IBSR and genD proposed by[40] to cope with hemizygous DNA, which represents a significant fraction of diploid grapevines.

We first defined the following genotypic table, where A is the reference allele and B is the alternate allele:

Site	Individual 1	Individual 2
0	AA	AA
	BB	BB
IBS=2	AB	AB
IBS=1	AB	AA or BB
	AA or BB	AB
IBS=0	AA	BB
	BB	AA

In each window, identity–by–state ratio ($IBSR_H$) was calculated using the following Equation **1** for all informative sites:

$$IBS{=}2+IBS{=}1 IBS{=}2+IBS{=}1+IBS{=}0 \qquad (\mathbf{1})$$

$IBSR_H$ is modified from the identity–by–state ratio (IBSR) originally used by Wu *et al.* [40]. $IBSR_H$ takes into account the fraction of IBS contributed by AB│A0 and AB│B0 variant sites in segments of hemizygous DNA.

Genotypic distance (genD) was calculated using the following Equation **2**:

$$((IBS{=}1 * 0.5) + IBS{=}0)IBS{=}0 + IBS{=}1 + IBS{=}2 + 0 \qquad (\mathbf{2})$$

Haplotypic distance (hapD) was calculated based on aggregate length of IBD=0, IBD=1 and IBD=2 windows using the following Equation **3**:

$$((IBD{=}1 * 0.5) + IBD{=}0)IBD{=}0 + IBD{=}1 + IBD{=}2 \qquad (\mathbf{3})$$

Supplementary references

1. Wan, Y. *et al.* A phylogenetic analysis of the grape genus (*Vitis* L.) reveals broad reticulation and concurrent diversification during neogene and quaternary climate change. *BMC Evol. Biol.* **13**, 141 (2013).
2. Svenning, J.-C., Normand, S. & Kageyama, M. Glacial refugia of temperate trees in Europe: insights from species distribution modelling. *J. Ecol.* **96**, 1117–1127 (2008).
3. Petit, J. R. *et al.* Climate and atmospheric history of the past 420,000 years from the Vostok ice core, Antarctica. *Nature* **399**, 429–436 (1999).
4. Terhorst, J., Kamm, J. A. & Song, Y. S. Robust and scalable inference of population history from hundreds of unphased whole genomes. *Nat. Genet.* **49**, 303–309 (2017).
5. De Lorenzis, G., Imazio, S., Brancadoro, L., Failla, O. & Scienza, A. Evidence for a sympatric origin of Ribolla gialla, Gonais blanc and Schiava cultivars (*V. vinifera* L.). *South African J. Enol. Vitic.* **35**, 149–156 (2014).
6. Lacombe, T. *et al.* Large-scale parentage analysis in an extended set of grapevine cultivars (*Vitis vinifera* L.). *Theor. Appl. Genet.* **126**, 401–414 (2013).
7. Chen, H., Patterson, N. & Reich, D. Population differentiation as a test for selective sweeps. *Genome Res.* **20**, 393–402 (2010).
8. Rubin, C. J. *et al.* Whole-genome resequencing reveals loci under selection during chicken domestication. *Nature* **464**, 587–591 (2010).
9. Massonnet, M. *et al.* The genetic basis of sex determination in grapes. *Nat. Commun.* **11**, (2020).
10. Nakano, M. *et al.* Next-generation sequence databases: RNA and genomic informatics resources for plants. *Plant Physiol.* **182**, 136–146 (2020).
11. Schwope, R. *et al.* Open chromatin in grapevine marks candidate CREs and with other chromatin features correlates with gene expression. *Plant J.* **107**, 1631–1647 (2021).
12. Rossmann, S. *et al.* Mutations in the miR396 binding site of the growth‐regulating factor gene *VvGRF4* modulate inflorescence architecture in grapevine. *Plant J.* **101**, 1234–1248 (2020).
13. Fasoli, M. *et al.* Timing and order of the molecular events marking the onset of berry ripening in grapevine. *Plant Physiol.* **178**, 1187–1206 (2018).
14. Massonnet, M. *et al.* Ripening transcriptomic program in red and white grapevine varieties correlates with berry skin anthocyanin accumulation. *Plant Physiol.* **174**, 2376–2396 (2017).
15. Royo, C. *et al.* The major origin of seedless grapes is associated with a missense mutation in the MADS-Box gene VviAGL11. *Plant Physiol.* **177**, 1234–1253 (2018).
16. Anderson, K. & Nalgen, S. *Which winegrape varieties are grown where ?* (University of Adelaide Press, 2020).

17. Zhou, Y., Massonnet, M., Sanjak, J. S., Cantu, D. & Gaut, B. S. Evolutionary genomics of grape (*Vitis vinifera* ssp. *vinifera*) domestication. *Proc. Natl. Acad. Sci. U. S. A.* **114**, 11715–11720 (2017).

18. Mercenaro, L., Nieddu, G., Porceddu, A., Pezzotti, M. & Camiolo, S. Sequence polymorphisms and structural variations among four grapevine (*Vitis vinifera* L.) cultivars representing sardinian agriculture. *Front. Plant Sci.* **8**, (2017).

19. Cardone, M. F. *et al.* Inter-varietal structural variation in grapevine genomes. *Plant J.* **88**, 648–661 (2016).

20. Carbonell-Bejerano, P. *et al.* Catastrophic unbalanced genome rearrangements cause somatic loss of berry color in grapevine. *Plant Physiol.* **175**, 786–801 (2017).

21. Da Silva, C. *et al.* The high polyphenol content of grapevine cultivar Tannat berries is conferred primarily by genes that are not shared with the reference genome. *Plant Cell* **25**, 4777–4788 (2013).

22. Di Genova, A. *et al.* Whole genome comparison between table and wine grapes reveals a comprehensive catalog of structural variants. *BMC Plant Biol.* **14**, 7 (2014).

23. Liang, Z. *et al.* Whole-genome resequencing of 472 *Vitis* accessions for grapevine diversity and demographic history analyses. *Nat. Commun.* **10**, 1190 (2019).

24. Ma, Z. Y. *et al.* Phylogenomics, biogeography, and adaptive radiation of grapes. *Mol. Phylogenet. Evol.* **129**, 258–267 (2018).

25. Girollet, N. *et al. De novo* phased assembly of the *Vitis riparia* grape genome. *Sci. data* **6**, 127 (2019).

26. Zdunić, G. *et al.* Genetic diversity of wild grapevine [*Vitis vinifera* L. subsp. *sylvestris* (Gmel.) Hegi] in the Eastern Adriatic Region. *Am. J. Enol. Vitic.* **68**, 252–257 (2017).

27. Marroni, F. *et al.* Reduction of heterozygosity (ROH) as a method to detect mosaic structural variation. *Plant Biotechnol. J.* **15**, 791–793 (2017).

28. Wang, K., Li, M. & Hakonarson, H. ANNOVAR: functional annotation of genetic variants from high-throughput sequencing data. *Nucleic Acids Res.* **38**, e164 (2010).

29. Choi, Y. & Chan, A. P. PROVEAN web server: A tool to predict the functional effect of amino acid substitutions and indels. *Bioinformatics* **31**, 2745–2747 (2015).

30. Crespan, M. *et al.* Unraveling the genetic origin of 'Glera', 'Ribolla Gialla' and other autochthonous grapevine varieties from Friuli Venezia Giulia (northeastern Italy). *Sci. Rep.* **10**, (2020).

31. Wales, N. *et al.* The limits and potential of paleogenomic techniques for reconstructing grapevine domestication. *J. Archaeol. Sci.* **72**, 57–70 (2016).

32. Jaillon, O. *et al.* The grapevine genome sequence suggests ancestral hexaploidization in major angiosperm phyla. *Nature* **449**, 463–7 (2007).

33. Martin, M. Cutadapt removes adapter sequences from high-throughput sequencing reads.

EMBnet J **17**, 10–12 (2011).

34. Li, H. & Durbin, R. Fast and accurate short read alignment with Burrows-Wheeler transform. *Bioinformatics* **25**, 1754–60 (2009).

35. Li, H. *et al.* The Sequence Alignment/Map format and SAMtools. *Bioinformatics* **25**, 2078–2079 (2009).

36. Peterson, B. K., Weber, J. N., Kay, E. H., Fisher, H. S. & Hoekstra, H. E. Double digest RADseq: An inexpensive method for *de novo* SNP discovery and genotyping in model and non-model species. *PLoS One* **7**, e37135 (2012).

37. Catchen, J., Hohenlohe, P. A., Bassham, S., Amores, A. & Cresko, W. A. Stacks: an analysis tool set for population genomics. *Mol. Ecol.* **22**, 3124–3140 (2013).

38. Rastas, P., Calboli, F. C. F., Guo, B., Shikano, T. & Merilä, J. Construction of ultradense linkage maps with Lep-MAP2: Stickleback F2 recombinant crosses as an example. *Genome Biol. Evol.* **8**, 78–93 (2016).

39. Van, H. *et al.* SMOOTH: a statistical method for successful removal of genotyping errors from high-density genetic linkage data. *Theor Appl Genet* **112**: 187-194 (2005)

40. Wu, G. A. *et al.* Sequencing of diverse mandarin, pummelo and orange genomes reveals complex history of admixture during citrus domestication. *Nat. Biotechnol.* **32**, 656–62 (2014).
