# Supplementary material for: The genomes of 204 Vitis vinifera accessions reveal the origin of European wine grapes
Source: Nat Commun. 2021 Dec 21;12:7240. doi: 10.1038/s41467-021-27487-y (PMC8692429; doi:10.1038/s41467-021-27487-y)
Supplement: Supplementary file 3 — Description of Additional Supplementary Files [file 41467_2021_27487_MOESM3_ESM.pdf]

Description of Additional Supplementary Files

File Name: Supplementary Data 1
Description: Metadata of WGS accessions.

File Name: Supplementary Data 2
Description: Three–population test with hybridization–based genotyping data in the diversity panel. Reported are the f3 statistics (only negative values), f3 standard error and Z–scores for all combinations of four groups shown in Fig. 1b of the main text. Groups of cultivated varieties are bold faced.

File Name: Supplementary Data 3
Description: Phenotypic data for berry–related traits. Berries were collected in the germplasm repositories of the University of Udine (Udine, 46.03 N, 13.23 E), Kmetijsko Gozdarski Zavod Nova Gorica (Vrhpolje, 45.86 N, 13.96 E) and VCR Research Center (Rauscedo, 46.06 N, 12.84 E), during the season 2016. Values represent the average of three replicates. Soluble sugar concentration was estimated using a hand refractometer with automatic temperature compensation. Sultanina and Kishmish vatkana carried only remains of the undeveloped seeds due to stenospermocarpy.
