# Supplementary material for: The genomes of 204 Vitis vinifera accessions reveal the origin of European wine grapes
Source: Nat Commun. 2021 Dec 21;12:7240. doi: 10.1038/s41467-021-27487-y (PMC8692429; doi:10.1038/s41467-021-27487-y)
Supplement: Supplementary file 7 — Reporting Summary [file 41467_2021_27487_MOESM7_ESM.pdf]

Corresponding author(s): Michele Morgante, Gabriele Di Gaspero

Last updated by author(s): Nov 11, 2021

Reporting Summary

Nature Portfolio wishes to improve the reproducibility of the work that we publish. This form provides structure for consistency and transparency in reporting. For further information on Nature Portfolio policies, see our Editorial Policies and the Editorial Policy Checklist.

Statistics

For all statistical analyses, confirm that the following items are present in the figure legend, table legend, main text, or Methods section.

n/a	Confirmed	
☐	☒	The exact sample size (*n*) for each experimental group/condition, given as a discrete number and unit of measurement
☐	☒	A statement on whether measurements were taken from distinct samples or whether the same sample was measured repeatedly
☐	☒	The statistical test(s) used AND whether they are one- or two-sided<br>*Only common tests should be described solely by name; describe more complex techniques in the Methods section.*
☒	☐	A description of all covariates tested
☒	☐	A description of any assumptions or corrections, such as tests of normality and adjustment for multiple comparisons
☐	☒	A full description of the statistical parameters including central tendency (e.g. means) or other basic estimates (e.g. regression coefficient) AND variation (e.g. standard deviation) or associated estimates of uncertainty (e.g. confidence intervals)
☐	☒	For null hypothesis testing, the test statistic (e.g. *F*, *t*, *r*) with confidence intervals, effect sizes, degrees of freedom and *P* value noted<br>*Give P values as exact values whenever suitable.*
☒	☐	For Bayesian analysis, information on the choice of priors and Markov chain Monte Carlo settings
☒	☐	For hierarchical and complex designs, identification of the appropriate level for tests and full reporting of outcomes
☐	☒	Estimates of effect sizes (e.g. Cohen's *d*, Pearson's *r*), indicating how they were calculated

Our web collection on statistics for biologists contains articles on many of the points above.

Software and code

Policy information about availability of computer code

Data collection	No software was used for data collection.
Data analysis	The CASAVA 1.8.2 version of the Illumina pipeline was used to process raw reads. Contaminant reads, including organelle sequences, were filtered using erne–filter version 1.2 and adapters were removed with cutadapt version 1.1. Short reads sequences were then mapped using the software package BWA version 0.7.5a. Raw variants were called using the UnifiedGenotyper tool in GATK version 3.3–0. The output of the aligner in Sequence Alignment/Map (SAM) format was sorted and transformed to Binary Alignment/Map (BAM) file through the software package SAMtools version 0.1.18. PCR duplicates were removed with samtools rmdup command and uniquely aligned reads were selected for further analyses. Repetitive DNA included high copy number sequences reconstructed by ReAS, transposable elements annotated in Repbase, and microsatellites ± 10 bp identified using the software Sputnik. RNA reads were aligned using GSNAP version 2019-03-04 and allele–specific RNA reads were counted with ALLIM version 1.1. Allele–specific expression levels were then determined by a STAR version 2.6 alignment of the same RNA reads against the reference genome. Gene phylogeny was inferred from maximum likelihood trees based on haplotype Muscle alignments curated by Gblocks. Trees were drawn with TreeDyn version 198.3. Population structure was obtained using ADMIXTURE version 1.23. Genetic ancestry was calculated using TreeMix version 1.12. Geographical maps of spatial population genetic structure were drawn using tess3r package in R. Trimming, aligning and SNP calling of GBS reads were carried out using the Stacks software package version 1.35. Genotyping errors were removed with the SMOOTH software. Genetic maps were generated with Lep–MAP2 version 0.2. GWAS and IBD estimates based on IBS at invididual variant sites were performed using PLINK version 1.07. The SMC++ software version 1.15.24 was used to infer demography. Graphs of genealogical relationships were drawn using the network package in R. Computation for the ABBA–BABA test was performed using the script ABBABABAwindows.py from the github platform (https://github.com/simonhmartin/genomics_general). Spearman correlation and GLM were computed using the lm function in R version 3.6.1.

For manuscripts utilizing custom algorithms or software that are central to the research but not yet described in published literature, software must be made available to editors and reviewers. We strongly encourage code deposition in a community repository (e.g. GitHub). See the Nature Portfolio guidelines for submitting code & software for further information.

Data

Policy information about availability of data

All manuscripts must include a data availability statement. This statement should provide the following information, where applicable:

- Accession codes, unique identifiers, or web links for publicly available datasets
- A description of any restrictions on data availability
- For clinical datasets or third party data, please ensure that the statement adheres to our policy

The DNA and RNA data generated in this study have been deposited in Sequence Read Archive (SRA) under the BioProject numbers PRJNA373967 (https://www.ncbi.nlm.nih.gov/bioproject/PRJNA373967), PRJNA390884 (https://www.ncbi.nlm.nih.gov/bioproject/PRJNA390884), PRJNA385116 (https://www.ncbi.nlm.nih.gov/bioproject/PRJNA385116), PRJNA321480 (https://www.ncbi.nlm.nih.gov/bioproject/PRJNA321480). The sequences of the reference genome used in this study are accessible under the BioProject number PRJEA18785 (https://www.ncbi.nlm.nih.gov/bioproject/PRJEA18785/). The phenotypic data generated in this study are provided as a Supplementary Data file. Source data are provided with this paper.

Field-specific reporting

Please select the one below that is the best fit for your research. If you are not sure, read the appropriate sections before making your selection.

☐ Life sciences ☐ Behavioural & social sciences ☒ Ecological, evolutionary & environmental sciences

For a reference copy of the document with all sections, see nature.com/documents/nr-reporting-summary-flat.pdf

Ecological, evolutionary & environmental sciences study design

All studies must disclose on these points even when the disclosure is negative.

Study description	We analysed WGS-derived short-read DNA sequences, using a reference-based alignment strategy, from a sample of 204 Vitis vinifera accessions (WGS panel) that captured the genetic diversity in the species, based on existing public information on the characterisation of grapevine germplasm repositories with SNP chips (diversity panel). DNA analyses were aimed at describing intraspecific genetic diversity and population structure as well as at characterizing genomic regions that showed low levels of nucleotide and haplotype diversity as a possible consequence of artificial selection. To finely characterize the genomic region that showed the lowest level of diversity, we generated RNA short reads and phenotypic data to investigate the impact of low diversity in the region on gene expression and berry-related traits.
Research sample	The research sample for WGS is a group of cultivated grapevine varieties that is compared to undomesticated accessions. The sample is meant to represent all major groups of wine grapes that provided the foundation of the global wine industry and to include controls from table grapes and undomesticated grapes. The rationale for this sample choice is functional to the aims of the study described in the panel above (which require that the analysed set captures most of the intraspecific variation in genetic diversity) and to address the evolutionary questions about the relationships between natural populations and botanical groups of cultivated varieties (which requires genetic variation within groups to be well represented). The study is also extended to a larger diversity panel (n = 1,445) using data from existing data sets cited in Figure 3 and reported in detail in Source Data. These datasets refer to grapevine accessions held in the largest European germplasm repositories of INRA Domaine de Vassal (France), IMIDRA Finca El Encin, Madrid and ICVV, Logroño (Spain) and JKI Geilweilerhof, Siebeldingen (Germany) [references in main text: Laucou et al 2018, Marrano et al 2018, De Lorenzis et al 2019, Ramos-Madrigal et al 2019] as well as to further germplasm from the South Caucasus that is not comprehesively represented in European gene banks [references in main text: De Lorenzis et al 2015]. These datasets represent the state-of-art for the known genetic variation in the species V. vinifera. All accessions therein are characterized at a common set of variant sites that could compared with WGS data. The rationales for including these datasets in the present study are: 1) to have a highly reliable representation and validation of the fraction of diversity captured by the WGS panel compared to the known diversity; 2) to extend analyses of population genetics for parameters that are first accurately defined in the WGS panel using millions of unbiased SNPs to the analysis of the most ample genetic datasets available from literature and public repository, though genotyped at a subset of variant sites.
Sampling strategy	Sample-size calculation was not predetermined. For evolutionary analyses based on whole-genome sequencing, the sample included 204 accessions that well represent the known diversity in the species. This sample size is sufficient to capture the known genetic diversity. We performed an a posteriori validation by comparing this sample (n = 204) with a larger diversity panel (n = 1,445) using GBS data from existing data sets. For RNA analysis and the characterization of the selective sweep on chromosome 17, we used a sub-set of accessions that carry in different diploid combinations the haplotypes that had been identified in the whole n = 204 sample.
Data collection	Berries of 88 varieties were sampled at same developmental stage on different dates (Supplementary Data 2), from two replicated field plots. From each plot two batches of asynchronous berries were collected over the same bunches, one composed of hard berries (target developmental stage: 5.2 °Brix), the other composed of soft berries (target developmental stage: 6.4 °Brix), both sorted by firmness to the touch. The accuracy of berry sorting was validated by subsampling from each batch random subsets of berries for destructive measurements, e.g. soluble solids concentration (Figure 6), berry weight, number of seeds per berry, seed fresh weight, and derived parameters (Supplementary Data 2). Data were collected by Gabriele Di Gaspero. Data of soluble solids concentration were visually read using hand-held refractometer with automatic temperature compensation (ATC-1, ATAGO CO., LTD, Saitama, Japan). All data mentioned here were recorded in excel spreadsheets.
Timing and spatial scale	Data collection started in 2016 on day of the year 204 (22 July 2016 Gregorian Date) and it was completed by day of the year 236 (23

August 2016 Gregorian Date). Each accession was sampled at a single time-point, corresponding to the exact day when hard and soft berries coexisted on the same bunches. The day of collection of each accession (when this condition occurred) is reported in Supplementary Data 2 along with soluble solids concentration data that account for the occurrence of this condition. The rationale for this timing in sampling was the necessity to collect berries from different accessions with different phenology at a synchronized developmental stage. The spatial scale from which the samples were taken is a geographical line spanning approximately 90 Km. The exact location of the three sampling sites along this line is 46.06 N, 12.84 E; 46.03 N, 13.23 E; 45.86 N, 13.96 E.

Data exclusions

One sample from the n = 204 set was excluded from certain analyses and this was stated in that part of the text.

"We first used a model–based clustering approach using whole genome sequence data of 203 accessions of vinifera (after removing accession KE–06 from this specific analysis, following the classification of this individual as a feral escapee done by Liang and coworkers)"

For genome–wide association analysis (GWAS) of the seed–to–berry ratio (reported in Supplementary Figure 20 for explaining the phenotypic effect of the selective sweep on chromosome 17), the accessions Sultanina and Kishmish Vatkana, which carried only remains of the undeveloped seeds due to stenospermocarpy–a trait controlled by an independent locus on chromosome 18–were excluded.

Reproducibility

Reproducibility of the results regarding the genetic structure of the grapevine germplasm, the relationships between populations and the origin of European wine grapes was obtained by testing alternative assumptions and different hypothetical evolutionary scenarios as reported in Supplementary Note 5 and Supplementary Figures S13–20.

Randomization

Population genetic analyses were conducted without a priori allocation of samples into groups, with one exception explained below. Grouping of samples for generating figures and for calculating parameters within populations was solely based on the information of DNA variation data, with an agnostic approach with regard to other assumptions, which we considered necessary for an unbiased approach. An exception to this rule was only done for generating Figure 1b, with the following rationale. A priori geographic clustering was used only for producing for the TreeMix analysis on the extended germplasm of the diversity panel. Individual varieties were assigned to a country of origin based on information reported in the Vitis International Variety Catalogue database or to the earlier or most renowned growing area. Countries were grouped into broad geographical areas that have homogeneous within–areas and differentiated between–areas climate conditions, following the Köppen-Geiger climate classification map. All groups were defined in the main article and the complete composition of all groups is reported in Source Data.

Blinding

Berries used for generating phenotypic data were sampled blind regardless their size, shape, color, position on the bunch, position on the vine.

Did the study involve field work? ☒ Yes ☐ No

Field work, collection and transport

Field conditions

Phenotypic data on berry parameters were generated from berries collected on plants grown in three germplasm repositories, University of Udine (Udine, Italy, 46.03 N, 13.23 E), Kmetijsko Gozdarski Zavod Nova Gorica (Vrhpolje, Slovenia, 45.86 N, 13.96 E) and VCR Research Center (Rauscedo, Italy, 46.06 N, 12.84 E), during the season 2016. Monthly averages of daily average temperature (°C) and total precipitation (mm) scored during the growing season 2016 at the closest meteorological stations were:

Udine: Apr, 13.7°C, 66.3 mm; May, 16.4°C, 238.3 mm; Jun, 21.2°C, 122.4 mm; Jul, 24.3°C, 92.3 mm; Aug, 22.4°C, 68.7 mm; Sep 20.6° C, 113.9 mm.

Rauscedo: Apr, 14.0, 66.1 mm; May, 16.5°C, 175.3 mm; Jun, 20.9°C, 112.7 mm; Jul, 23.6°C, 69.0 mm; Aug, 21.7°C, 163.8 mm; Sep 20.0°C, 68.9 mm.

Vrhpolje: Apr, 13.1°C, 73.4 mm; May 16.0°C, 123.4 mm; Jun, 20.7°C, 162.8 mm; Jul, 23.9°C, 67.6 mm; Aug, 22.6°C, 74.9 mm; Sep 20.0°C, 108.2 mm.

Location

Udine, Italy, 46.03 N, 13.23 E 88 m.a.s.l.
Vrhpolje, Slovenia, 45.86 N, 13.96 E, 136 m.a.s.l.
Rauscedo, Italy, 46.06 N, 12.84 E, 130 m.a.s.l.

Access & import/export

No natural habitats have been accessed for sampling

Disturbance

Leaf and berry samples for DNA and RNA sequencing were collected from plant stocks held by germplasm repositories.

Reporting for specific materials, systems and methods

We require information from authors about some types of materials, experimental systems and methods used in many studies. Here, indicate whether each material, system or method listed is relevant to your study. If you are not sure if a list item applies to your research, read the appropriate section before selecting a response.

Materials & experimental systems

n/a	Involved in the study
☒	☐ Antibodies
☒	☐ Eukaryotic cell lines
☒	☐ Palaeontology and archaeology
☒	☐ Animals and other organisms
☒	☐ Human research participants
☒	☐ Clinical data
☒	☐ Dual use research of concern

Methods

n/a	Involved in the study
☒	☐ ChIP-seq
☒	☐ Flow cytometry
☒	☐ MRI-based neuroimaging
